# Genomic Signatures of North American Soybean Improvement Inform Diversity Enrichment Strategies and Clarify the Impact of Hybridization

**DOI:** 10.1534/g3.116.029215

**Published:** 2016-07-07

**Authors:** Justin N. Vaughn, Zenglu Li

**Affiliations:** * Center for Applied Genetic Technologies, University of Georgia, Athens, Georgia 30602; † Department of Crop and Soil Science, University of Georgia, Athens, Georgia 30602

**Keywords:** selection on standing variation, haplotype frequencies, detecting selection, maturity groups

## Abstract

Crop improvement represents a long-running experiment in artificial selection on a complex trait, namely yield. How such selection relates to natural populations is unclear, but the analysis of domesticated populations could offer insights into the relative role of selection, drift, and recombination in all species facing major shifts in selective regimes. Because of the extreme autogamy exhibited by soybean (*Glycine max*), many “immortalized” genotypes of elite varieties spanning the last century have been preserved and characterized using ∼50,000 single nucleotide polymorphic (SNP) markers. Also due to autogamy, the history of North American soybean breeding can be roughly divided into pre- and posthybridization eras, allowing for direct interrogation of the role of recombination in improvement and selection. Here, we report on genome-wide characterization of the structure and history of North American soybean populations and the signature of selection in these populations. Supporting previous work, we find that maturity defines population structure. Though the diversity of North American ancestors is comparable to available landraces, prehybridization line selections resulted in a clonal structure that dominated early breeding and explains many of the reductions in diversity found in the initial generations of soybean hybridization. The rate of allele frequency change does not deviate sharply from neutral expectation, yet some regions bare hallmarks of strong selection, suggesting a highly variable range of selection strengths biased toward weak effects. We also discuss the importance of haplotypes as units of analysis when complex traits fall under novel selection regimes.

The study of crop domestication inspired many of the earliest insights in evolutionary biology (Ross-Ibarra *et al.* 2007). Domestication loci have historically been detected through the phenotypic characterization of crosses between wild and domesticated species (Doebley *et al.* 2006). The transition from a wild species to a domesticated one can involve selection on rare or even naturally deleterious alleles such as nonshattering fruit (Meyer and Purugganan 2013), although long-term improvement likely depends on genetic variants at intermediate frequencies in the progenitor population (Beissinger *et al.* 2014). With the advent of genome-wide genotyping, researchers began to compare the genetic diversity of particular genomic regions within wild progenitor species with the diversity in their cultivated relatives, under the assumption that strong artificial selection would have fixed both the causal variants and the neutral polymorphisms surrounding those variants in the domesticate (Morrell *et al.* 2012), a process often referred to as a “selective sweep” (Maynard-Smith and Haigh 1974). Thus, without knowing the domestication phenotype, a list of domestication loci could be generated (Zhao *et al.* 2015; Zhou *et al.* 2015). Correlations between domestication analysis and genome-wide association studies are beginning to reveal the phenotypic consequences of these loci (Wen *et al.* 2015; Zhou *et al.* 2015). Even without knowing the mode of action underlying an allele’s benefit, identification of such loci will also likely enhance the efficacy of crop breeding and genomic selection (Morrell *et al.* 2012).

The Huang-Huai Valley in China appears to be the center of origin of domestication for soybean (Han *et al.* 2016). This domestication event led to the creation of thousands of landraces, defined generally as unimproved varieties with nonvining growth form, reduced seed-shattering, and lighter seed coat. Starting in 1765, a small subset of these landraces was gradually introduced into North America (Hymowitz and Harlan 1983). Because soybean is highly autogamous, its improvement has occurred in two major phases; line selection, which was limited by the availability of imported landraces, followed modern selection based on controlled crosses (Carter *et al.* 2004a). There is no explicit record of the breadth of soybean germplasm that was available to early farmers in North America, but it appears that a substantial amount of the genetic diversity found in landraces was present in known North American soybean ancestors (Hyten *et al.* 2006). In spite of this diversity, it is known through pedigree analysis that only a few of these ancestors contributed substantially to the breeding programs and thus to modern elite germplasm (Gizlice *et al.* 1994). This biased contribution could have multiple causes, but the most obvious is that these lines were the dominant soybean varieties in production. This dominance was, in turn, the result of more than a century of line selection that, in effect, filtered the landrace germplasm into a handful of geographically structured colonies (Carter *et al.* 2004a). One aim of this research was to determine the degree to which this earlier colony structure manifested itself after the first breeding programs were established and controlled crosses became routine, referred below as the “posthybridization era.”

The early phase of soybean development entailed the selection of lines that were optimal for particular growing regions. Desirable traits were likely to be quite similar in the prehybridization and posthybridization eras [although initially introduced as a forage crop, soybean was harvested primarily for its seed beginning in the 1860s (Probst and Judd 1973)]. Yet, given a wild outcrossing rate of <1%, alleles favored by selection would rarely be able to recombine with beneficial alleles present in other introduced lines during the prehybridization phase (Carter *et al.* 2004a). Once crossing facilitated this recombination, the haplotypes containing the combination of beneficial alleles might displace the original haplotypes, thereby reducing diversity across that region of the genome. An additional aim of this research was to identify the ancestral alleles that, in part, were responsible for the early success of their donors in the prehybridization era and to evaluate the change in allele frequencies, diversity levels, and haplotype structure after hybridization became common practice.

Two recent studies have compared elite soybean cultivars from North America with a sample of globally distributed landraces (Wen *et al.* 2015; Zhou *et al.* 2015). In both of these studies, the elite lines used are primarily grown in the Midwest and Northern US. Both studies identified regions that either exhibit reduced diversity or aberrant population differentiation relative to the genome-wide average. Zhou *et al.* (2015) also characterized F_st_ values between US and Canadian varieties relative to four landrace subpopulations, noting that sharp distinctions in allele frequencies for known genes, such as E1, a maturity gene, and the T locus, which controls tawny *vs.* gray pubescence. Zhou *et al.* (2015) also noted that many of these genomic regions, though very likely domestication or improvement loci, were not detected based on landrace-by-elite comparisons. Instead, detection required more detailed analyses across subpopulations. It also remains difficult to differentiate which regions identified in these studies were the result of selection or were the result of the narrow founding bottleneck that occurred for North American subpopulations.

Nearly all crops have undergone a population bottleneck and consequent loss of genetic diversity relative to their wild progenitors (Kovach and McCouch 2008). In soybean, genetic diversity across global germplasm is effectively half that of its wild progenitor (Hyten *et al.* 2006; Lam *et al.* 2010). This bottleneck can be a consequence of the fact that only a small family or, in the extreme case, a single individual, possess the desired features for agricultural production. Such scenarios establish genome-wide linkage disequilibrium (LD) between neutral alleles and those alleles underlying desirable traits, and this LD will substantially complicate the interpretation of loci identified by solely comparing pre- with postbottleneck populations. Still, knowledge of these patterns will be important for informing diversity enrichment strategies, and careful characterization of the postfounding population structure should allow us to more effectively differentiate reductions in diversity related to the bottleneck from those related to selection that occurred later, during modern North American improvement.

Many statistics designed to identify selection serve as a proxy for the rate of allele frequency change. For example, extended linkage disequilibrium (Sabeti *et al.* 2002) is based on finding genomic regions in which selection has outpaced recombination’s ability to statistically decouple alleles at different loci. While this method and its variants are very powerful, the ability to monitor allele frequency changes through time still offers the most direct measure of selection, particularly on the time-scale of contemporary crop improvement (Bollback *et al.* 2008; Feder *et al.* 2014). Indeed, detection of selection using time-serial allele frequency changes should be more sensitive than other established methods to alleles segregating at high and intermediate frequencies prior to the application of artificial selection. Because soybean lines are effectively “immortalized genotypes,” many lines sampling the last century of soybean breeding have been genotyped using SoySNP50K Infinium Chips (Song *et al.* 2013), thus creating an experimental system that facilitates time-serial analysis.

Researchers in maize have recently begun to explore the genome-wide effects of long-term selection in experimental populations. Generally, these experiments are based on selection for phenotypes that are components of yield such as seed-size (Hirsch *et al.* 2014) and ear-number (Beissinger *et al.* 2014). These studies used pooled samples of progenitor and postselection populations and scanned the genome for extreme levels of population differentiation, or F_st_. Generally, these studies found that selection on standing variation was much more common than selection on new mutations and that selected alleles rarely reached fixation, potentially because the time-scale of the analysis was too short or that many of the favorable alleles exhibit some degree of dominance. The dominant mode of selection in agricultural populations remains an open question with many implications for crop improvement (van Heerwaarden *et al.* 2012). The final aim of this work was to use soybean improvement over the last century to help more fully define modes of artificial selection, and to provide strategies for further genetic gains in soybean yield.

## Materials and Methods

### Genotypic data

Genotyping of the USDA Soybean Germplasm Collection, where all lines in this study have been deposited, was previously performed using the SoySNP50K iSelect BeadChips (Song *et al.* 2013). The data were accessed from http://soybase.org/data_distribution/soybase_soy50K_snp_all_cultivars_and_snps.gz on February 6, 2014. Data were processed as described previously (Vaughn *et al.* 2014). Physical distances described in this manuscript are based on genome assembly version Glyma.Wm82.a1 (Gmax1.01) (Schmutz *et al.* 2010); the distances are slightly shifted in version Glyma.Wm82.a2 (Gmax2.0). All marker information is given in Supplemental Material, File S4.

### Population analysis

**Figure 1:**
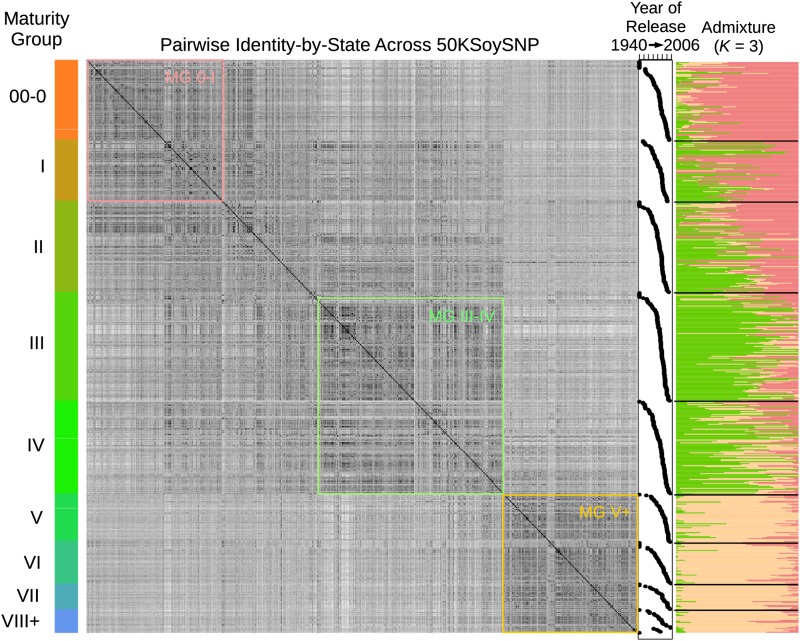
Population structure of modern public soybean varieties. Rows in the symmetrical IBS matrix are sorted by maturity group (right panel) and then by date-of-release as indicated in adjacent xy plots. Population assignments and admixture are shown in the far-right panel given a model of three populations (see *Material and Methods*). Colored boxes within the matrix indicate how the total set of accessions was divided into populations for further analysis. SNP, single nucleotide polymorphism.

Identity-by-state (IBS) matrices were generated using the command-line implementation of TASSEL 4.0 (-distanceMatrix) (Bradbury *et al.* 2007). Population structure was assessed using ADMIXTURE (version linux-1.23) with *K* = 3 (Alexander *et al.* 2009) [*K* was set to 3 based on the pattern observed when ordering lines based on maturity (Figure 1)].

### Fractional contribution by haplotype sharing

Because of the very low heterozygosity levels in soybean, each marker was determined to be most closely related to one of 29 major North American ancestors based on the ancestor with which it had the longest match length to the left and right of each marker. These 29 ancestors were chosen based on their percent contribution as assessed previously using pedigree analysis (Gizlice *et al.* 1994), although some first progeny were used when an original ancestor was not available. The longest matching ancestor haplotype was required to be >5 markers longer than the second longest haplotype match. A threshold of 5 was chosen after simulations indicated that between 1 and 8 markers were sufficient to identify identical-by-descent regions from the correct grandparents of recombinant inbred lines simulated using random sets of genotypes from the 29 North American ancestors (data not shown). More stringent length thresholds (>8) began to sharply increase the number of markers within a line that were deemed ambiguous. The threshold was held constant across the genome since the physical positions of markers on the SoySNP50K chip were chosen to roughly approximate genetic distance: ∼0.1 cM per marker (Song *et al.* 2013). Heterozygous markers, which only occur ∼14 times per genotype on average, were not penalized as they are generally the result of technical genotyping errors [roughly 40% of heterozygotes are likely true homozygotes (Ben Stewart-Brown, personal communication)], or heterogeneity at a few loci within a single line. In such cases, the burden of differentiating haplotypes is passed to down/upstream markers. Each member of each population, MG 0-I, MG III-IV, or MG V+ (as described in *Results*), was assessed, and the number of markers traced to a particular ancestor was tallied for every ancestor and divided by the total markers. Markers for which the longest haplotype could not be determined or was ambiguous were not counted in the genome-wide average. Scripts used to perform key aspects of this analysis, among others associated with this study, are available as in File S3.

### Diversity analysis

TASSEL 5.0 was used to calculate average pairwise difference, π, between haplotype windows for a given set of lines. A sliding window that was 50 markers wide was moved in 10 marker increments across the length of each chromosome. TASSEL options were as follows: -diversity, -diversitySlidingWin, -diversitySlidingWinStep 10, and -diversitySlidingWinSize 50. The units of π are the number of pairwise differences per marker averaged across the sliding window. The π measure for all 29 North American ancestors, π*_a_*, was compared with π for lines released prior to the 1970s within each population, π*_x_*, and log_2_(π*_x_* / π*_a_*) value was used to characterize changes in diversity. By normalizing by π*_a_*, this statistic allows comparison across loci of various starting diversity levels. The log_2_ transformation is an extension of that rationale in that when two separate loci start at the same diversity level, a reduction to 25% the original diversity, for example, should appear twice as substantial as a reduction to 50%.

### Effective population size, N_e_, based on temporal fluctuations in allele frequency

For each population, we grouped varieties released from the 1940s, 1950s, and 1960s and used these allele frequencies for generation 0. Only loci with major alleles that started at a frequency between 0.5 and 0.6 were used for the initial timepoints. This frequency filtering was done in order to avoid analyzing loci with alleles that commonly reappear after fixation as a result of statistical sampling. Varieties released in each additional decade (the 1970s, 1980s, 1990s, and 2000s) were grouped separately, and we assessed allele frequencies within these decades, treating them as sequential samples. The effective population size was estimated for each set of temporal data points using the R package NB (Hui and Burt 2015) with the following parameters: “alleles=rep(2,*x*), sample.interval=c(0,15,10,10,10), profile.likelihood=FALSE, and bound=c(20,1000),” where *x* is the number of loci that had an initial allele frequency between 0.5 and 0.6. The generational intervals (“sample.interval”) were selected based on the effective number of generations we assumed to have occurred during each decade or combination of decades and the average midpoint of the years of release within each group.

### Power analysis to detect selection

We evaluated the statistical power of four possible measures of selection based on the set of simulations defined in Table 4. For each simulation, the population was held constant at 200 and allele frequencies were assessed at the generations used above: 0, 15, 25, 35, and 45. We assessed power using both the true population frequency and the frequency as estimated given the sample depth typical of our study (Table 4). The power is reported as the percentage of selected loci that surpass the threshold giving a 5% false positive rate when both selected and neutral loci are combined in equal parts (neutral distributions were taken from the simulation with the same initial allele frequency of the beneficial allele under selection). Additionally, when assessing power, absolute values of all measures were used because we will not know the relationship between the polarity of an allele’s selection coefficient and its initial frequency in real data. Linkage disequilibrium between loci was not considered.

For F_st_ measures and linear regression, only the timepoints up to and including the first timepoint at which an allele reached fixation (<5% or >95%) were used. The slope of the linear regression was estimated using the Perl module Statistics::LineFit, reported as Δf. The logistic regression coefficient (reported as “logistic β” in Table 4), was calculated, using the R command “glm(formula = y ~ x, data = d, family = binomial),” where *y* is the allele frequency at decade-by-decade time times used above, *x* is time, and *d* represents the dataframe. F_st_ was calculated as the probability that two alleles sampled from a single timepoint are identical relative to the probability that two alleles sampled from the combined first and last timepoints are identical (Hamilton 2011). The WFABC algorithm was used to directly estimate the selection coefficient (Foll *et al.* 2015). Default parameters were used for wfabc_1 and wfabc_2, except -ploidy was set to 1 because all lines are recombinant inbred lines. To avoid computational instability in the WFABC algorithm, N was held fixed at 200 - the actual size in the simulation - when initial frequency was 0.8 and true selection coefficient was 0.1.

### Scans for selection

We used the N_e_ estimates in Table 3 for each population and simulated a distribution of Δf expected for a set of neutral alleles (Figure S3). Sequential timepoint samples were used as for N_e_ estimation above, except that all loci with an initial major allele frequency <0.95 were included, not just those between 0.5 and 0.6. The starting frequency distribution of simulated, neutral alleles was designed to reflect that of the actual markers in each population.

Thresholds for putatively selected regions were determined based on the shape and variance of total distributions of Δf and log_2_(π*_x_* / π*_a_*). See Table 5 and Figure 4 for population-specific thresholds.

### Haplotype spectra analysis

All possible haplotypes within a 50 marker window were identified and their frequencies assessed across each chromosome in 10 marker increments as done for the diversity analysis above. Each unique haplotype in the pre-1970s sample was assigned a color in order of highest to lowest frequency (Figure 5). Haplotypes in later samples were assigned the same color if a color had already been assigned in the earlier sample. Gray was used for additional haplotypes if more than seven haplotypes had already been observed in a given window.

For each window, the top three largest rates of change (|Δf|) for individual markers were averaged and reported along with the most rapid change for a haplotype within the same window. The use of three alleles was a heuristic approach to balance a) the excessive noise of using only the single highest marker in a window and b) the dramatic reduction in sensitivity associated with taking the average across the entire window. Average pairwise length of shared haplotypes (*H*) was assessed using H-scan (version 1.3) developed by Philip Messer (https://dl.dropboxusercontent.com/u/77898333/H-scan.cpp).

### Data availability

The authors state that all data necessary for confirming the conclusions presented in the article are represented fully within the article.

## Results

### Maturity defines population structure in modern varieties

Our total dataset consisted of 579 soybean varieties released from <1940 to 2009 (Table S1). Each line was genotyped using the SoySNP50K Infinium Chip as part of USDA’s effort to characterize the entire *Glycine* germplasm bank. The data are publicly available. Historical analysis in maize indicates that modern breeding for heterotic groups strongly differentiated North American populations (van Heerwaarden *et al.* 2012). Soybeans are not released as hybrids, but they are very sensitive to photoperiod; therefore, flowering and maturation time are expected to be major variables in structuring the cultivated soybean population. We analyzed the IBS across all accessions in this study. When sorted based on maturity group (MG), three highly similar groups emerge (Figure 1). The pattern is generally defined by the similarity of MG 0-I lines, III-IV lines, and V+ lines. Lines were subordered within each maturity group based on year-of-release, but there were no evident patterns associated with this parameter. Thus, lines have been comparably differentiated into three maturity-group populations since the very earliest stages of modern soybean breeding. This supports and expands prior results based on pedigree analysis that indicated very distinct ancestral contributions to modern Northern *vs.* Southern cultivars (Gizlice *et al.* 1994). In short, a small number of dominant lines quickly established their territories, the boundaries of which have been in place ever since. The exception appears to be the MG II lines, which, prior to ∼1970s, fall into the MG 0-I cluster. After the 1970s, these lines exhibit substantial admixture between the MG 0-I and MG III-IV groups. For this reason, in further analysis, MG II lines were removed, reducing the total genotypes from 579 to 487. We treated the three remaining clusters as independent populations, which will be referred to as “MG 0-I,” “MG III-IV,” and “MG V+” throughout the remainder of the manuscript (Figure 1).

### North American soybean ancestors have biased IBS between populations

**Figure 2:**
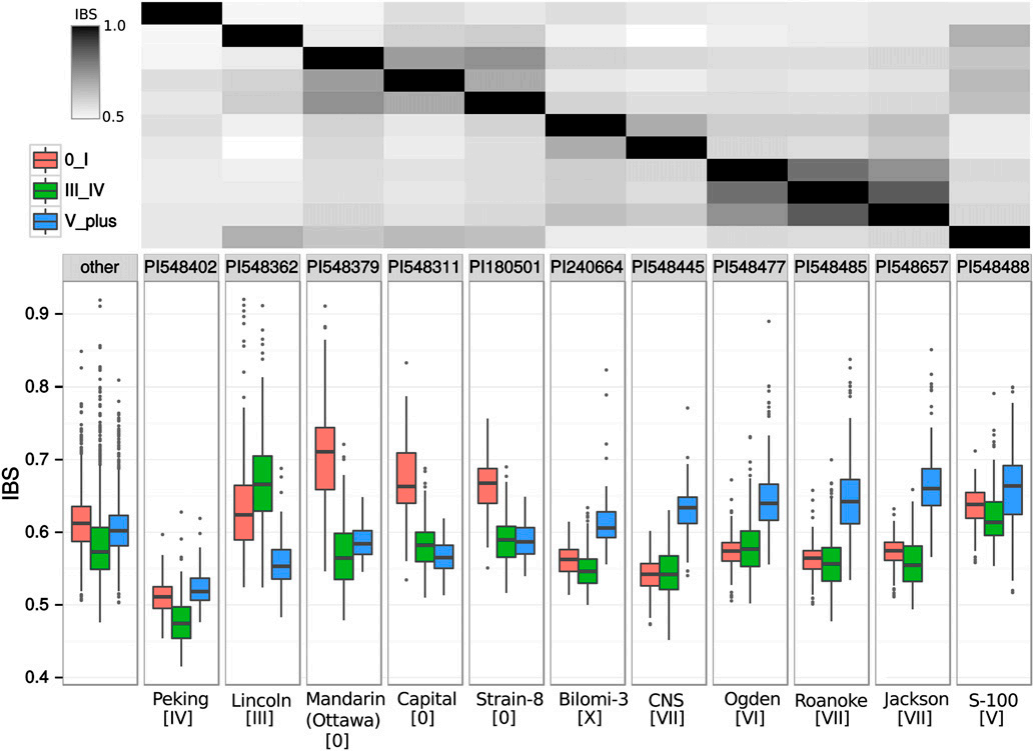
Biased population IBS with major ancestors. For each population, the IBS between each individual and the indicated ancestor is given as a boxplot. Pairwise IBS between each ancestor is shown at the top as a heatmap matrix in which black represents an IBS of 1, or perfect identity. The “other” category represents combined results from all other ancestors in Figure S1. Maturity group of the ancestor is indicated in brackets below name. PI548445 is shown independently because it in an outlier for its low IBS relative to all other ancestors. IBS, identity-by-state.

As described above, it is known from pedigree analysis that Northern and Southern breeding lines are derived from distinct ancestry. Therefore, it is likely that populations 0-I, III-IV, and V+ each have very distinctive profiles of ancestry. To investigate this possibility, we calculated the average IBS of each population relative to each of the 29 major North American ancestors (Figure S1). Nearly every ancestor shows a significantly higher IBS with one or more populations over another (Figure S1). In Figure 2, we emphasize the ancestors with the most substantial relatedness and/or differential relatedness to populations.

Distinct landraces clearly have a differential relatedness to the three elite populations (Figure 2). Generally, these results compliment prior work based on pedigree analysis (Gizlice *et al.* 1994). Key ancestors such as Lincoln, Mandarin (Ottawa), CNS, and S-100 show significantly higher IBS to specific populations, and these lines are genetically distinct from one another, suggesting distinct genetic contributions. Identity to population 0-I is dominated by Mandarin (Ottawa) and Capital, although Capital does have fairly high IBS with Mandarin (Ottawa). In addition, Lincoln also has a higher than average IBS with this population. Population MG III-IV is highly related to Lincoln, whereas population MG V+ appears to have three major ancestors: CNS, Roanoke, and S-100. S-100 is unique in that it appears to make similarly high contributions to all populations, perhaps because of its similarity to Lincoln.

The relationship of elite population structure to the North American soybean ancestors has implications for which genomic regions experienced reduced diversity in the earliest lines developed from hybridization breeding. Diversity within those early populations could be reduced because of selection or because there was reduced diversity among the lines that served as parents in the vast majority of crosses resulting in those lines (see *Introduction*). To achieve a more refined measurement of the degree to which an ancestral line contributed to a population, we compared the haplotypes of elite lines within each population released prior to the 1970s to the haplotypes of all 29 major North American ancestors (see *Materials and Methods*).

The results of this haplotype analysis (Table 1) generally support our IBS analysis (Figure 2 and Figure S1), but they also clarify situations in which one ancestor shares substantial haplotype structure with another ancestor but did not contribute to the same population, as in the case of S-100 [Jackson (PI 548657) was excluded from this haplotype analysis because it was introduced in the 1950s and is 85% identical to Roanoke (PI 548485) (Figure 2)]. In addition, the results are strikingly similar to those seen in prior pedigree-based studies (Gizlice *et al.* 1993), yet, while those studies only emphasized Northern *vs.* Southern germplasm, we have further subdivided the Northern germplasm. The differential contribution of Mandarin (Ottawa) suggests a major cause for the early and stable distinction between these MG0-I and MGIII-IV populations (Table 1). The ancestry for the MGIII-IV is generally the most homogenous; the single cultivar Lincoln has a fractional contribution of nearly 0.3. It is also interesting that, though Peking has a very low IBS (Figure 2), it has >0.03 fractional contribution to the MGV+ population (Table 1). This finding is consistent with the historical use of Peking as an early donor of soybean cyst nematode (SCN) resistance in Southern but not Northern varieties (Carter *et al.* 2004b).

**Table 1:** Fractional contribution based on haplotype sharing for major North American ancestors relative to varieties released prior to 1970 within each population

ID	Name	MG 0-I	MG III-IV	MG V+
PI548362	Lincoln	0.23	0.29	0.02
PI548379	Mandarin (Ottawa)	0.26	0.03	0.01
PI548488	S-100	0.02	0.07	0.20
PI548485	Roanoke	0.03	0.04	0.19
PI548445	CNS	0.00	0.04	0.18
PI548406	Richland	0.12	0.14	0.02
PI548477	Ogden	0.01	0.04	0.14
PI548391	Mukden	0.07	0.04	0.02
PI548318	Dunfield	0.02	0.07	0.04
PI548461	Improved Pelican	0.00	0.01	0.07
PI548311	Capital	0.05	0.03	0.01
PI548382	Manitoba Brown	0.05	0.00	0.01
PI548360	Korean	0.02	0.05	0.02
PI548325	Flambeau	0.04	0.01	0.00
PI548352	Jogun	0.01	0.03	0.00
PI548402	Peking	0.00	0.01	0.03

Only ancestors with values >0.03 are shown. ID, identifier.

In the remainder of the manuscript, we treat ancestors with a fraction contribution >0.03 as “founding ancestors” of a particular population. This distinction is in contrast to the group of all 29 major North American ancestors used above.

### Reduced diversity in founding ancestors dictates regions of reduced diversity in posthybridization cultivars

**Figure 3:**
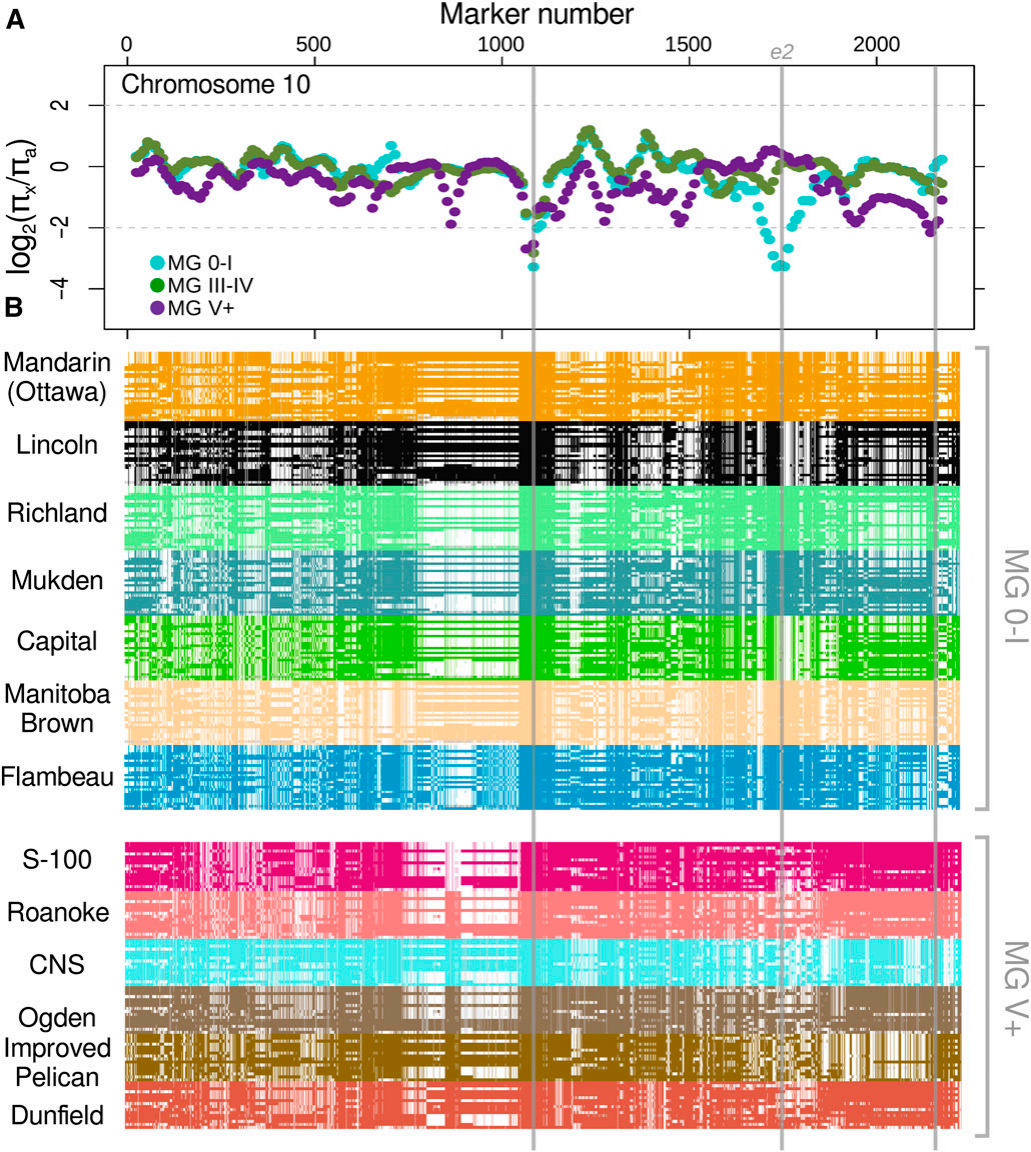
Relationship between reduced diversity and founding ancestors of pre-1970s varieties within each population. Only chromosome 10 is shown as a representative example; similar plots for all chromosomes are available as supplemental material (File S1). (A) Log_2_ ratio of mean pairwise difference of a given population (π*_x_*) relative to all 29 North American ancestors (π*_a_*) is given for each window of 50 markers along the chromosome. Markers are numbered 1 through the last marker on the chromosome in order of physical position (File S4). (B) The identity of each marker relative to the given ancestor is depicted for each of the pre-1970s lines from populations MG 0-I and MG V+. Each individual is shown as a row and, therefore, appears as many times as there are ancestors. A locus is colored if it is identical to the ancestor. White space indicates a mismatch. Heterozygous markers, though rare, are colored gray. Gray vertical lines spanning the figure indicate regions of reduced diversity. Position of maturity gene, *e2*, is labeled. The trough at position 1074 is off scale for all populations (see Table 2).

With regard to the biased contribution of different ancestors, we attempted to determine if a region of reduced diversity in a population resulted from the pool of founding ancestors being fixed at that locus or from a single ancestral haplotype that was rapidly fixed in early stages of regional breeding. For this analysis, genotyping information for each population was divided into windows that were 50 markers long. For each of these windows, we assessed the log_2_ ratio of genetic diversity (π) of the earliest stages of the posthybridization era (pre-1970s), π*_x_*, to that of all 29 North American ancestors, π*_a_*. Generally, this diversity measure varies between 2 and −2. Values were rarely >2, thus, diversity was rarely substantially increased (Figure 3A and File S1).

Using chromosome 10 as an example (Figure 3), there are three regions of reduced diversity. The first region exhibits reductions in all three populations (Figure 3A). The second region is unique to MG 0-I. The third, minor trough is unique to MG V+. Figure 3B depicts the identity of each marker of each individual variety in the pre-1970s populations relative to their respective set of founding ancestors. In regions of reduced diversity, we would expect homogeneity across all lines, yet the identity with ancestors should be different if diversity was present in that region among the founding ancestors. In other words, strong vertical patterning is indicative of reduced diversity in a population; if the pattern is solid across all ancestors, then the haplotype was fixed in most of the early crosses. Alternatively, if diversity was present, then early selection may have caused the reduction in diversity. It should also be noted that some regions will have reduced diversity in a population but will not show a particularly low log_2_ diversity ratio. This result is due to there being little or no diversity among all 29 North American ancestors.

Table 2 summarizes the results of this analysis, and all graphical files comparable to Figure 3 are available as supplemental material (File S1). In nearly all cases, a single haplotype appears in all or most of the founding ancestors at regions of reduced diversity. Because there is variation for these regions across the total set of ancestors (otherwise the log2 diversity ratio would be ∼0), these loci could possibly represent regions that are important to their specific regions of cultivation. The most obvious of these would be genomic intervals containing genes related to flowering time. Indeed, all four of the major effect maturity genes, *e1–e4*, are present at a threshold of <2.3 (Table 2). Interestingly, for all low diversity genomic regions proximal to maturity genes, founder column is less than 100% in Table 2 (and File S1); therefore, the ancestors were segregating for haplotypes that were eventually fixed by 1970. In the MG 0-I population, the ancestral haplotype common to Lincoln and Capital was lost in favor of the more common alternative haplotype (Figure 3). The alternative haplotype is found in the majority of MG 0-I ancestors such as Mandarin (Ottawa), Manitoba Brown, and Flambeau. Interestingly, Lincoln is a maturity group III line, but Capital, a maturity group 0 line, shares the Lincoln haplotype. Moreover, while Richland and Mukden are maturity group II, they have the Mandarin (Ottawa) haplotype. It is known that maturity genes interact in complex ways, particularly in early maturity groups (Tsubokura *et al.* 2014), but it is clear that the Mandarin (Ottawa) haplotype for *e2* was favored by early breeders.

**Table 2:** Characterization of regions of reduced diversity in founding ancestors of each population

Chr.	Pop.	Percent Founders*^a^*	Midsite Marker*^b^*	Start (bp)	Stop (bp)	log_2_(π*_x_*/π*_a_*)	π*_a_*	Major QTL/Gene*^c^* or Selective Sweep*^d^* Overlap
8	0-I	86	2014	38,409,071	39,587,082	<−8	0.1	Wen *et al.* 2015
10	0-I	100	1074	28,888,713	32,173,874	<−8	0.05	
10	III-IV	100	1074	28,888,713	32,173,874	<−8	0.05	
10	V+	100	1074	28,888,713	32,173,874	<−8	0.05	
18	0-I	100	2274	48,936,186	49,279,506	<−8	0.18	
20	V+	100	544	18,760,809	22,082,754	<−8	0.08	
6	V+	83	1034	16,372,276	16,548,439	−6.53	0.23	
16	V+	50	1214	29,198,889	29,895,378	−5.3	0.29	Zhou *et al.* 2015
1	0-I	100	504	7,476,077	10,015,701	−4.34	0.16	Zhou *et al.* 2015
1	V+	67	1484	52,033,784	52,821,919	−4.28	0.28	Methionine
19	0-I	86	1444	36,758,516	37,272,085	−4.24	0.22	Zhou *et al.* 2015
1	0-I	100	1234	49,196,746	49,637,775	−3.47	0.35	
17	0-I	100	324	4,161,627	4,680,961	−3.46	0.25	
14	0-I	86	1214	19,720,771	25,312,530	−3.32	0.22	Wen *et al.* 2015
20	V+	67	684	26,836,040	29,865,868	−3.31	0.2	Protein/yield
10	0-I	71	1754	43,758,245	44,436,997	−3.27	0.32	Maturity-*e2*
20	0-I	71	714	28,639,256	31,998,840	−3.26	0.12	Protein/yield
8	0-I	100	2184	40,881,278	41,453,586	−3.22	0.21	
12	V+	67	334	2,681,036	3,072,635	−3.22	0.29	
7	0-I	100	1264	15,793,416	16,436,790	−3.2	0.28	
11	III-IV	75	1434	33,511,555	34,570,537	−3.04	0.14	
11	0-I	100	704	10,516,051	11,429,744	−2.87	0.15	
17	0-I	43	1044	14,159,743	15,834,164	−2.86	0.27	Zhou *et al.* 2015
17	V+	67	874	13,006,407	13,154,755	−2.82	0.4	
19	0-I	100	304	3,591,338	4,759,347	−2.8	0.12	
13	V+	67	94	857,022	1,644,249	−2.78	0.31	
13	V+	67	94	857,022	1,644,249	−2.78	0.31	
20	V+	83	1354	42,078,113	43,040,384	−2.74	0.25	Seed weight
4	III-IV	33	1884	48,242,486	48,922,546	−2.63	0.29	Wen *et al.* 2015
11	V+	67	1424	33,371,745	34,403,523	−2.57	0.14	
20	V+	50	774	32,581,226	33,137,092	−2.56	0.22	Maturity-*e4*
19	III-IV	67	2024	46,033,555	47,088,579	−2.51	0.29	Maturity-*e3*
12	0-I	100	534	5,163,152	6,044,298	−2.49	0.17	Pubescence form
11	0-I	86	1434	33,511,555	34,570,537	−2.48	0.14	
11	V+	100	634	8,633,864	9,963,410	−2.46	0.2	
18	0-I	100	2134	47,719,925	48,017,046	−2.45	0.4	Wen *et al.* 2015
14	III-IV	100	1204	19,264,654	23,701,369	−2.39	0.22	
12	V+	67	834	8,419,651	9,023,940	−2.35	0.23	Zhou *et al.* 2015
8	V+	83	154	2,331,207	2,729,661	−2.35	0.31	
9	0-I	100	424	5,002,375	5,769,646	−2.35	0.36	
6	V+	67	1214	18,916,841	21,745,751	−2.32	0.38	Maturity-*e1*, Zhou *et al.* 2015

Chr., chromosome; Pop., population; QTL, quantitative trait locus.

a Percent of founding ancestors possessing the major haplotype.

b Markers are indexed from 1 to the total markers for that chromosome based on genomic position as depicted in Figure 3.

c Based on cloned genes deposited in Soybase and publicly available data from three genome-wide association studies (Vaughn *et al.* 2014; Sonah *et al.* 2015; Zhou *et al.* 2015).

d Identified as improvement sweeps in Wen *et al.* (2015) or Zhou *et al.* (2015).

It is rare for any population to completely lose diversity across a 50 bp window (values <−8 in Table 2). Extreme diversity ratio (<−8) can be the result of there being very little diversity in North American ancestors and that diversity being lost. This appears to be the case for the extreme chromosome 10 values shared by all populations (Table 2), although clearly there are exceptions. It is also rare for all three populations to have the same region of reduced diversity (Table 2, but more easily seen in File S1).

### Effective population size correlates with number of major founding ancestors

Because populations are finite, evolution occurs due to genetic drift regardless of selection. The effective population size, N_e_, is the population size that, if the loci comprising the sample were neutral and individuals were randomly mating, would produce the observed dispersion of allele frequencies from one generation to the next. Generally, estimates of N_e_ are important in generating a null expectation of allele frequency change. Starting with posthybridization lines released prior to 1970 and ending with lines released in the 2000s, we tracked the decade by decade change of allele frequencies within each population. We used a likelihood approach based on the variance in allele frequency change to predict N_e_ (Hui and Burt 2015). Because of the likelihood framework, the approach rigorously accounts for experimental sampling as well. Still, biased sampling could pose a problem to this analysis and those that follow. An examination of principal component (PC) plots based on genome-wide marker data within each population indicates that sampling was generally random (Figure S2), although there does appear to be mild population differentiation when moving from pre-1970s populations to 2000s populations (bottom panels in Figure S2).

N_e_ for each population is given in Table 3. Population MG V+ has the largest N_e_, followed by 0-I and then III-IV. Inbreeding reduces N_e_ because, in effect, it is structuring one population into several subpopulations due to the nonrandom mating of genotypes. The use of a single genotype as a parent within a population over time is one form of inbreeding. In turn, our N_e_ estimates correlate with the homogeneity of parentage within each population: MG V+ has three major ancestors, MG 0-I has two, and MG III-IV has only one (Lincoln) (Figure 2 and Table 1).

**Table 3:** Effective population size (Ne) estimates for populations

Population	Total Individuals Per Timepoint	Total Markers*^a^*	N_e_	95% C.I.
MG 0-I	27, 20, 40, 34, 16	8123	172	166–178
MG III-IV	31, 28, 59, 59, 22	6903	115	112–118
MG V+	16, 22, 24, 38, 32	6625	273	260–287

C.I., confidence interval.

a Number of markers with a major allele frequency between 0.5 and 0.6 in the first timepoint sample.

### Selection is typically on standing variation, but “haplotype sneaks” are also common

The ability to estimate selection coefficients from time-serial data are an area of active research (Bank *et al.* 2014). We compared one such published algorithm, WFABC (Foll *et al.* 2015), with the commonly used F_st_ statistic, as well as two simple statistics based on ether linear or logistic regression. We simulated the allele frequency trajectory based on the number of generations in our sample and a population size of 200, a value comparable to relevant N_e_ estimates (Table 3). The range of initial frequencies and selection coefficients are given in Table 4.

**Table 4:** Power of assorted statistics in the detection of selection at a 5% false positive rate for selection coefficients 0.02, 0.05, 0.1, and initial favored allele frequencies of 0.2, 0.5, and 0.8

Initial Frequency of Favored Allele	0.2			0.5			0.8			
Selection Coefficient	0.02	0.05	0.1	0.02	0.05	0.1	0.02	0.05	0.1	Average Power
Without sampling: sample frequency equals population frequency										
Δf	14.2	52.3	99.5	11.6	43.3	94.0	9.3	19.1	48.4	43.52
Logistic β	2.6	5.5	59.4	12.8	50.5	97.9	11.5	24.9	61.7	36.31
F_st_	23.6	69.4	99.6	13.1	48.6	97.0	0.7	0.0	0.0	39.11
WFABC *s*	4.4	13.2	80.6	14.0	44.3	96.1	13.1	25.0	54.0	38.3
With sampling: n = (31, 28, 59, 59, 22)*^a^* for generations = (0, 15, 25, 35, 45), respectively										
Δf	9.3	38.8	95.3	10.8	31.3	80.4	8.5	13.9	26.2	34.94
Logistic β	2.8	7.7	58.4	12.5	45.2	93.8	12.3	27.2	61.8	35.74
F_st_	19.0	58.2	99.6	11.3	33.7	62.8	1.6	1.9	2.1	32.24
WFABC *s*	0.3	0.0	0.0	9.0	24.0	58.4	NA*^b^*	NA	NA	15.2

Δf, frequency change per generation; Fst, fixation index; WFABC, Wright–Fisher ABC-based approach; NA, not applicable.

a Based on MG III-IV sampling depth.

b WFABC algorithm failed when favored allele frequencies were 0.8 and sampling was used.

While the mean WFABC prediction of selection coefficients was fairly accurate (File S5), the variance in estimates resulted in poor power to differentiate selected loci at a 5% false positive rate (Table 4). F_st_ was most powerful when selected alleles started at low frequencies, but was very limited when the selected allele started at high frequencies. This limitation is because F_st_ can only take advantage of end points in the allele trajectory, whereas the other three estimates use intermediate points up to fixation. The two simplest measures, Δf and the logistic coefficient, behaved similarly although the logistic regression performed inferiorly at low initial frequencies and superiorly at high frequencies. Statistical sampling reduced the power of all methods, as expected, although the logistic coefficient was less affected. Because we are uncertain as to the distribution of initial frequencies of selected alleles in our real data, we used Δf for the remainder of the analyses and the manuscript.

Though it is often rightly assumed that in natural populations the vast majority of polymorphisms should behave neutrally, this will not always be the case in breeding populations, which have high linkage disequilibrium (LD) and have been under intensive selective pressure. Indeed, we know that variation in allele frequencies is increased by selection in the populations used herein, although we cannot be sure of the magnitude. If selection has, in fact, driven many alleles to higher frequency, the observation that few values extend beyond the distribution under neutral expectation (Figure S3) indicates that selected loci are likely of small effect and, after including the markers in high LD with selected loci, comparable in number to neutral loci. Given the difficulty in devising an appropriate null distribution (see *Discussion*), the loci with the strongest signatures of change are the best candidates for regions under strongest selection (Akey *et al.* 2002; Beissinger *et al.* 2014; Hirsch *et al.* 2014). Using population specific thresholds, we established a set of putatively selected regions based on Δf, and on the reduction in diversity between the pre-1970 lines in a population and those released in the 2000s (Table 5).

**Table 5:** Counts and relative frequencies of selection modes for sliding window analysis

	MG 0-I	MG III-IV	MG V+	All
Total windows	4151	4151	4151	12453
Δ*f* threshold	0.013	0.018	0.014	NA
Diversity threshold	1.9	1.9	1.3	NA
Haplotype sneak*^a^*	216 (53%*^b^*)	166 (38%)	38 (13%)	420 (37%)
Hard sweep	16 (4%)	15 (4%)	32 (10%)	63 (6%)
Soft sweep	173 (43%)	246 (58%)	237 (77%)	656 (58%)

a See Figure 4 for additional clarification of terminology.

b Percent of total putatively selected regions.

Strong selection that acts on a new or introduced allele, a hard selective sweep, will produce a signature of rapid allele frequency change and reduced diversity around the allele due to the fixation of nearby neutral alleles in linkage. Soft sweeps, on the other hand, will exhibit a substantial change in allele frequencies without this extreme change in diversity. Thus, though they are related, the two measures used above—change in individual allele frequency and reduction in diversity across a window of loci—are potentially sensitive to distinct possibilities in the evolutionary history of a genomic region.

We performed a diversity analysis in the manner described above except that the lines released between 1940 and 1970 within a given population were treated as the ancestor population, and the lines released after year 2000 were compared with these populations. Individual markers with the top three highest Δf within each diversity window were averaged and plotted by the relative diversity within a window (Figure 4). We defined categories shown in Figure 4 using thresholds based on the shape of total distributions and the estimated N_e_ (Table 5). As is common in genome-wide characterization of sweeps, there is rarely enough data to differentiate selection on a new mutation from selection on very rare standing variation. Our approach is also susceptible to confusing these two conceptually distinct scenarios, although we feel the operational definitions used herein give an accurate impression of which modes of selection are most common.

**Figure 4:**
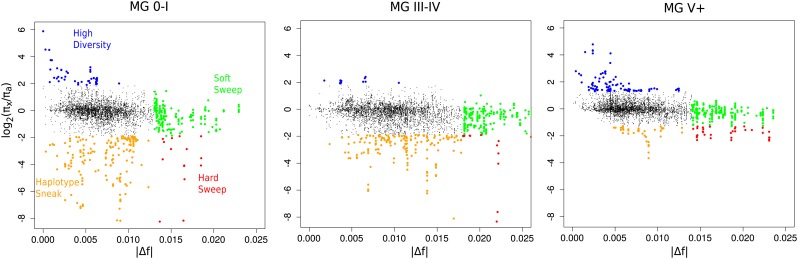
Modes of selection acting within and across each population. Each point represents the results for a window of 50 markers incremented by 10 markers along the chromosome. The diversity of lines released in the 2000s relative to lines released prior to 1970 is plotting on the y-axis for each population. The average of the top three absolute values of Δf for each window is plotted on the x-axis. Categories are color coded based on the thresholds defined in Table 5.

MG 0-I had the most dynamic range of values, although all populations exhibited comparable relationships between hard and soft sweeps (Table 5). The enrichment of soft sweeps relative to hard sweeps is expected from breeding populations, which are known to have rapid response to selection due to the presence of standing variation (Falconer and Mackay 1996), and the finding corroborates with other studies on long-term selection projects (Beissinger *et al.* 2014; Hirsch *et al.* 2014) as well as historical genomic analysis (van Heerwaarden *et al.* 2012). It should be noted that some soft sweeps may be incomplete sweeps that simply have not risen to high enough frequencies to dramatically change diversity; indeed, in certain contexts incomplete sweeps may actually increase diversity before decreasing it.

Interestingly, many regions exhibited a substantial reduction in diversity while not showing a resultant increase in Δf (Figure 4). This signal persisted at a similar scale when low diversity ancestral regions (π*_a_* < 0.1) were removed (data not shown) (generally, there was only a very weak correlation between reduction in diversity and the ancestral diversity level). This result indicates that a genomic region has become fixed without a substantial change in constituent allele frequencies. We refer to these windows as “haplotype sneaks” for the remainder of the manuscript (Figure 4 and Table 5). Three possible origins of haplotype sneaks are: 1) an initially high frequency haplotype goes to fixation, 2) a rare untyped variant becomes a target of strong selection, or 3) recombination creates a rare haplotype that sweeps through the population.

**Figure 5:**
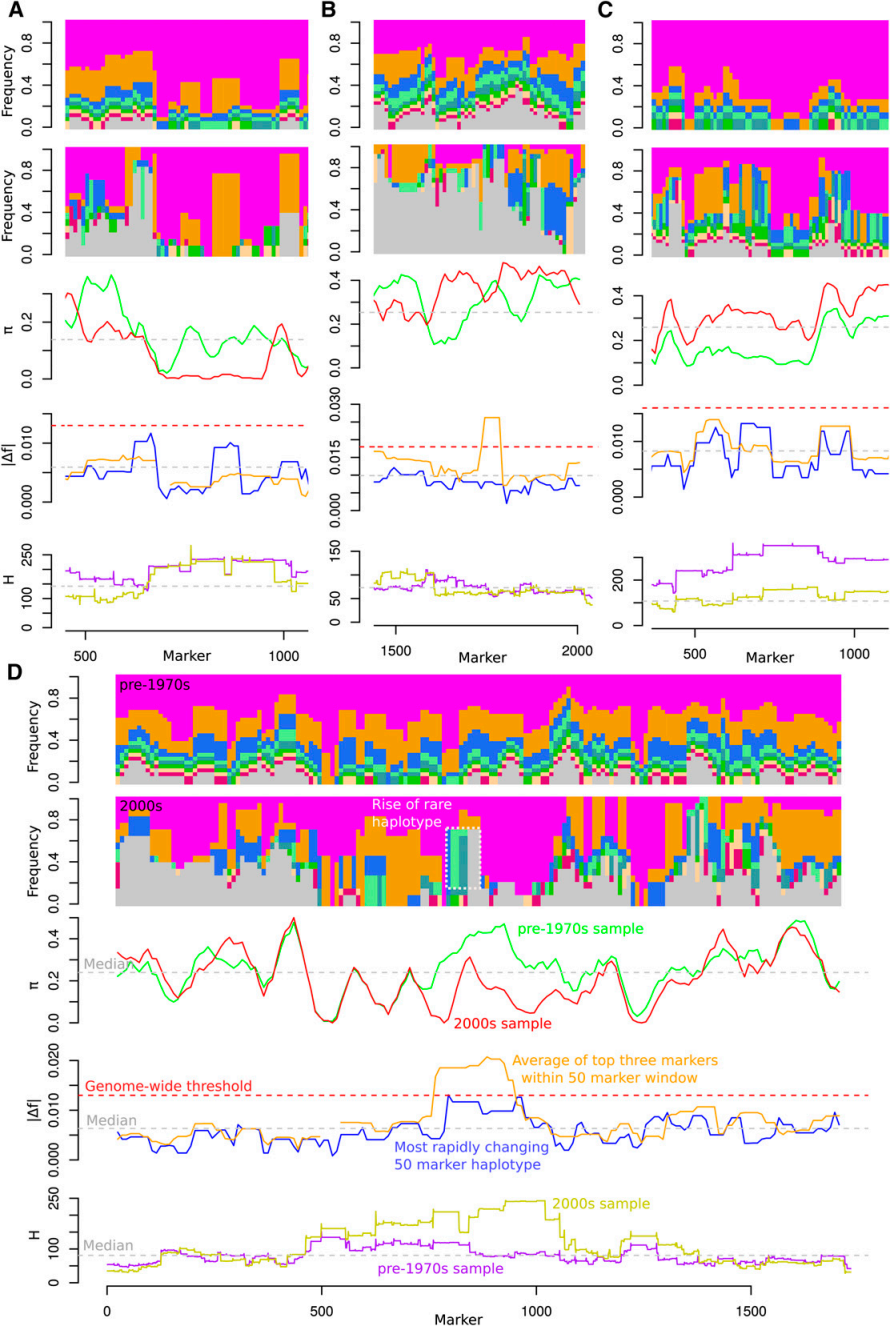
Haplotype spectra associated with selection. Top two panels within each subfigure show the frequency of each 50 marker haplotype, in 10 marker increments, along a chromosome for pre-1970s and 2000s samples in a given population. Markers are numbered 1 through the last marker on the chromosome in order of physical position (File S4). Haplotypes are ordered in the top panel, purple to crimson, based on frequency in pre-1970s sample. All additional haplotypes are colored gray. The same haplotype in both samples, pre-1970s and 2000s, will have the same color, excepting gray haplotypes. Note that shared colors left to right along the chromosomes could represent unlinked haplotypes although similar frequencies (and frequency changes) generally suggest linkage. The third panel shows average pairwise difference (π) for 50 marker windows; lines are colored based on indicated samples. In the fourth panel, blue lines indicate the frequency change of the most rapidly changing haplotype within a window. Orange lines indicate the average change of the three most rapidly changing alleles in a window. Haplotype change is based on the first (pre-1970s) and last (2000s) samples, whereas allele change is based on all sampled decade groupings (see *Materials and Methods*). The last panel depicts the average shared haplotype length (H) around a marker for pre-1970s (purple) and 2000s (yellow) samples. Gray dotted lines throughout indicate the median of values in a panel, while red lines, when present, indicate genome-wide thresholds described in Table 5 and shown in Figure 4. See *D* for annotation of figure elements. (A) Section of chromosome 11 from population MG 0-I. (B) Section of chromosome 5 from MG III-IV. (C) Section of chromosome 19 from MG V+. (D) Entire chromosome 1 from MG 0-I.

To differentiate Type 1 from Type 2 or 3 above, we tabulated all possible 50-marker-long haplotypes (in 10 marker increments) across the genome for each population (Figure 5). We tracked the change in these haplotypes from the pre-1970s sample to the 2000s sample. In addition, we overlaid pairwise diversity measures, maximum allele and haplotype changes within a window, and average length of shared haplotypes within the samples. Entire plots for all chromosomes for all populations are available as supplemental material (File S2). Across all populations, we found 15 examples of Type 2|3 regions, although 73% occurred in the MG 0-I population. As an example, a ∼500 marker-wide region from chromosome 11 of MG 0-I has three subregions, with haplotypes that changed more rapidly than any of the underlying markers (Figure 5A). This region also shows many of the hallmarks of a hard-sweep, except that there are no dramatic changes of any single marker frequency. As emphasized above, such signatures may be indicative of selection on a recombinant *or* on a rare, untyped variant that has become selectively advantageous.

Across all populations and genomic windows, the ratio of greatest-haplotype-change to greatest-individual-allele-change for haplotype sneaks is 18% greater than neutral windows as defined in Figure 4 (p-value = <10^−6^). The inclusion of neutral regions or regions selected for single alleles will reduce this ratio. Thus, the median value of 0.86 among haplotype sneaks, though higher than neutral, does not exceed 1. Moreover, haplotype change was assessed based on pre-1970s samples relative to 2000s samples, whereas Δf is only evaluated prior to fixation across all decades and, therefore, can have a higher value even if total change is the same. Still, composite graphs across all populations (File S2) rarely reveal examples, such as Figure 5A, of a haplotype sneak being associated with a striking change in any haplotype frequency. More commonly, haplotype sneaks are simply related to the fixation of large chromosomal regions within a population over time.

Using haplotype spectrum plots, we also interrogated soft sweeps, which were the most common mode of selection detected in our data (Figure 4 and Table 5). For example, an allele on chromosome 5 in population MG III-IV changes rapidly, but there are effectively no other signals indicating selection on this region (Figure 5B). This is suggestive of selection on standing variation, in that the locus under selection had already extensively recombined with other linked segregating loci prior to the change in selective regime. Indeed, marker-level analysis indicates that three distinct haplotypes, all containing the most rapidly changing markers, rose at comparable rates (not shown).

Haplotype spectra plots from MG V+ population (Figure 5C and File S2) show that large genomic tracks can have increases in π and decreases in H relative to pre-1970s cultivars, as was suggested by Figure 4. When averaged across the genome, H was somewhat reduced in both MG 0-I and MG V+, from a median value of 107–86 and 88–68, respectively. Thus, the reductions in H suggest that LD associated with the early population structure in the pre-1970s sample was steadily lost as a result of germplasm sharing and hybridization across breeding programs throughout the second half of the 20th century. The median genome-wide log_2_ diversity ratios were slight: –0.05 and –0.01 in MG 0-I and V+ populations, respectively, likely reflecting a balance between increased diversity related to recombination and reduced diversity related to selection. In contrast, the MG III-IV population exhibited a genome-wide increase in H from 77 to 112 in pre-1970s populations relative to 2000s, and a sharper reduction in diversity with a median log_2_ ratio of –0.19.

## Discussion

### Can we utilize selected alleles across populations?

The genetic diversity in modern cultivars is fairly representative of that found in North American ancestors (Hyten *et al.* 2006). This finding is correct based on diversity across all North American elite cultivars, yet the population as a whole is also highly structured (Figure 1). In this regard, the effective diversity available to regional breeding programs is much narrower. In addition, the fractional contribution of ancestors within each population is very uneven (Figure 2 and Figure 3). Still, after initial population structure was established, diversity has generally been maintained (File S2), although in many cases selection has reduced the diversity locally (Figure 5, A–D). It remains a standing challenge in the breeding community as to how to retain beneficial alleles already present in breeding germplasm while also exploring the utility of exotic material in elite backgrounds. A major method to address this goal involves developing near-isogenic lines (NILs) that introduce genes from exotic material (Imai *et al.* 2013). Since this approach is very laborious and costly, we hope our results will help breeders to more effectively choose regions that might benefit from the introduction of exotic alleles and to identify parents that would deliver those alleles.

Anecdotal evidence among soybean breeders has long held that elite Northern by Southern crosses rarely result in agronomically valuable progeny. Our population structure analysis supports these observations, in that admixed lines of MG V+ and either MG 0-I or MG III-IV are rare (Figure 1). This agronomic “incompatibility” is to a large degree explained by the low probability of accumulating the appropriate maturity genes together with a transgressive (or even average) segregant for yield (Jiang *et al.* 2014). Epistatic effects may also play a role with regard to simpler traits such as lodging that can have a substantial impact on yield. Our results in identifying regions under breeding selection suggest an experimental program for using marker-assisted introgression in order to test transferable yield alleles across these populations. We have produced a list based on whether an allele’s frequency change in one population exceeds thresholds given in Table 5 and if the same allele is fixed in the opposite direction of selection in at least one other population (Table 6). While some of these alleles were fixed early in breeding (Δ*f* = ∼0, final freq. = ∼0 or ∼1 in Table 6), most were still segregating after the 1970s. Those that were fixed prior to our sampling may represent regions that were responsible for founder success. Alternatively, they may have simply been lost by chance; in which case, they could potentially be reintroduced through marker-assisted selection to improve the recipient population. Whatever the effect on yield, the result of such an introgression would be interesting: either the effect is positive and the allele gives a generic yield benefit, or the effect is negative or neutral and the allele was selected in a distinct population because it is specifically advantageous to a particular environment or genetic background.

**Table 6:** Tagging markers for haplotype blocks putatively selected in one population but fixed in the opposite direction in another

Chr.	Marker Index*^a^*	Position	Ref.*^b^*	MG 0-I		MG III-IV		MG V+	
				Δ*f**^c^*	Final Freq.	Δ*f*	Final Freq.	Δ*f*	Final Freq.
2	39	558,323	C	−9.8	0	3.8	0.64	19.08*	0.95
2	337	4,551,551	C	−14.15*	0.31	−5.13	0.64	11.37	0.96
2	1047	11,998,550	C	−13.35*	0.31	−0.24	0.59	5.71	0.96
3	59	592,600	T	0	0.96	−6.04	0.68	−15.51*	0
6	355	7,683,418	A	−14.61*	0.06	2.97	0.64	0	1
6	1201	19,407,046	A	−0.23	0.88	32.01*	0.96	−5.07	0.02
7	595	8,112,122	C	−0.38	0.94	−5.04	0.55	−17.86*	0
13	1495	28,550,563	A	−13.81*	0	−0.68	0.93	6.85	0.81
13	2263	36,616,135	A	−15.76*	0.06	−14.44	0.32	1.22	0.95
15	677	9,508,185	G	3.94	1	−7.03	0.55	−14.7*	0.25
15	682*^d^*	9,544,360	T	−14.32*	0	3.95	0.82	8.93	1
15	857	11,416,165	G	17.53*	0.97	−2.75	0.59	−5.97	0.05
17	468	6,742,263	C	0	1	−10.4	0.36	−22.32*	0
18	2555	53,152,286	C	−4.87	0.06	−4	0.59	16.84	0.95
19	1817	42,812,863	T	3.74	0.5	20.49*	0.96	−8.44	0.04
20	1450	44,469,797	A	21.16*	1	4.88	0.8	0	0

Chr., chromosome; Ref., reference; Δ*f*, frequency change per generation; Freq., frequency.

a For cross-reference to figures in File S2.

b Δ*f* and final frequencies are relative to the major allele in MG 0-I, pre-1970s sample.

c Δ*f* (frequency change per generation) are multiplied by 1000 for ease of presentation and asterisks (*) indicate regions that are beyond population thresholds given in Table 5.

d Though part of the same linkage block, two representative markers are given because of high and contrasting rates in both MG 0-I and MG V+.

### Detecting selection within individual populations

Detection of selection is complicated by the fact that neutral allele frequencies also change over time in finite populations. Advantageous alleles are expected to change frequency more rapidly over many generations than neutral alleles. Though we can assess this rate of allele frequency change in this study, it remains a challenge to determine if the extreme values that we observe in allele frequency change could be predicted by genetic drift alone. The distribution of a given statistic under neutral expectation can be solved or simulated using the estimated effective population size (N_e_). As described in the *Results*, it is likely inappropriate to assume that the majority of loci are behaving in a neutral fashion in crop populations under active selection for a complex trait. This is not only because of selection, but because of the high LD present in these populations. Therefore, establishing an appropriate null model remains extremely challenging. Even in an experimental population with known demographics, selection and other modes of nonrandom mating can result in a twofold reduction in N_e_ estimates calculated directly from demographic parameters (Beissinger *et al.* 2014).

There are numerous statistics available for detecting selection. These different statistics are sensitive to different evolutionary time-scales and to how the data are structured (Vitti *et al.* 2013). For example, F_st_ values are often used to detect selection since neutral loci will estimate a single F_st_ value indicative of population divergence and migration, while selected alleles will have a variety of F_st_ values that are greater or less than the neutral F_st_, depending on the type of selection (Lewontin and Krakauer 1973). In modern incarnations, the neutral F_st_ estimate and its variance are derived from a genome-wide set of markers (Akey *et al.* 2002). While the statistic has shown some efficacy, F_st_ is generally measured between two populations or two timepoints. Recently, the genotyping of experimental populations has generated interest in algorithms designed to exploit data from samples taken at multiple timepoints for a single population (Bank *et al.* 2014). Herein, we used a simple regression approach to estimate allele frequency change prior to fixation as a function of time, Δ*f*. In simulations, this simplistic approach had more power than F_st_ (Table 4) and both statistics are more powerful than attempts to directly estimate the selection coefficient. The time-serial approach also appears to be more sensitive to selection on standing variation than average shared haplotype length (Figure 5B). Though they are both dependent on the change in allele frequency prior to fixation, the advantage of Δf over F_st_ is related to fact that F_st_ is ignoring the time-scale over which the change occurs. In simulations, both measures generally had a consistent neutral distribution regardless of initial allele frequency (File S5).

It has long been appreciated that simultaneous selection at multiple loci can dramatically affect selection efficacy (Fisher 1930). In the context of artificial selection acting on standing variation in a complex trait, it may be quite common for two or more loci with substantial independent effects to be in tight linkage, and these could either be in negative or positive phase. If in negative phase with comparable effects, the Hill–Robertson effect will dominate (Hill and Robertson 1966). If the key recombinant occurs prior to drift removing one of the beneficial alleles, the rare haplotype will rapidly rise in frequency. Such a scenario may explain the observed excess of recombined parental haplotypes in the progeny of some breeding pedigrees (Lorenzen *et al.* 1996). Importantly, this change may not have nearly as comparable an effect on the underlying allele frequencies, although they too should rise. We considered if selection on such key recombinations was one of the underlying causes of the haplotype sneaks that we observed (Figure 4 and Table 5).

Figure 5D depicts what might at first appear to be a classical hard sweep on chromosome 1 in the MG 0-I population; diversity is substantially reduced, allele frequencies show a rapid change, and, as should follow diversity reductions, the average shared-haplotype length for that region becomes longer. The haplotype spectra indicate that a haplotype initially present at low frequencies rose to ∼78% by the 2000s. Again, this suggests a hard sweep. Yet, what is less explicable is that the purple haplotype, which was common in the pre-1970s, also rose rapidly to near fixation by the 2000s. Indeed, the most rapidly changing marker falls within this region. We hypothesize that the changes in these two regions were not coincidental, and that the purple haplotype had initially been linked to a deleterious region that was inhibiting its rise to fixation. Marker-level analysis across this region indicated that the gray haplotype in the 2000s sample was in fact a recombinant between the green and purple haplotypes that appeared very early in MG 0-I breeding (data not shown). Unfortunately, the green/gray haplotype only appeared in this early sample in linkage with the purple haplotype, and so we cannot rule out that selection was simply on a rare allele that was linked to the purple haplotype by chance (and that the position of peak Δf was also due to chance fluctuation in the unlinked purple haplotype frequency). As emphasized by this example, even signatures of “hard sweeps” can have multiple interpretations when selection is acting on numerous QTL simultaneously. High coverage resequencing can be used to identify and characterize rare variants in a sample and will allow more precise definition of such sweeps. Still, even with resequencing data, our results indicate that the composite perspective of both haplotype and single-marker analyses could be critical in interpreting the results of artificial and natural selection research. The further integration of these methods with time-series models should be a fruitful aim for future studies.

As made clear by this study and many others, it can be very difficult to define an appropriate null distribution for selection. As it eliminates the need to estimate a null distribution from the tested data, perhaps the most useful methodology for understanding selection in crop improvement will involve analyzing genome-wide marker data for hundreds of members of known breeding pedigrees in which resultant progeny were the product of selection (Sebastian *et al.* 1995; Jannink *et al.* 2001). Many crop communities possess “immortalized” genotypes of milestone cultivars and derived lines. We expect these datasets to be enlightening not only for applied breeding but for evolutionary biology as well.

## Supplementary Material

Supplemental Material
