# Supplementary material for: Genomic Signatures of North American Soybean Improvement Inform Diversity Enrichment Strategies and Clarify the Impact of Hybridization
Source: G3 (Bethesda). 2016 Jul 7;6(9):2693–705. doi: 10.1534/g3.116.029215 (PMC5015928; doi:10.1534/g3.116.029215)
Supplement: Supplemental Material [file supp_6_9_2693__index.html]

Genomic Signatures of North American Soybean Improvement Inform Diversity Enrichment Strategies and Clarify the Impact of Hybridization — Supplemental Material 

# Genomic Signatures of North American Soybean Improvement Inform Diversity Enrichment Strategies and Clarify the Impact of Hybridization

## Supplemental Material for Vaughn and Li, 2016

**Files in this Data Supplement:**

- Figure S1 - Boxplots representing the average IBS between each ancestor and the indicated population. (.pdf, 37 KB)
- Figure S2 - PC plots based on genome-wide marker information across all lines in the analysis. (.pdf, 80 KB)
- Figure S3 - The distribution of cumulative allele frequency changes under a drift-only model is indicated in red. (.tif, 399 KB)
- File S1 - A collection of files depicting the information in Figure 3 for all 20 chromosomes. (.zip, 3 MB)
- File S2 - Full figures for each chromosome in each population as depicted in Figure 5. (.zip, 2 MB)
- File S3 - Assorted Perl and R scripts associated with analysis and graphing methods. (.zip, 6 KB)
- File S4 - All marker information including marker number as depicted in Figure 3 and 5. (.txt, 2 MB)
- File S5 - Combined histogram for all simulated statistics in power analysis (Table 4). (.zip, 49 KB)
- Table S1 - PIs used in the analysis and their assorted characteristics. (.xls, 66 KB)
