# Supplementary material for: Genomic Signatures of North American Soybean Improvement Inform Diversity Enrichment Strategies and Clarify the Impact of Hybridization
Source: G3 (Bethesda). 2016 Jul 7;6(9):2693–705. doi: 10.1534/g3.116.029215 (PMC5015928; doi:10.1534/g3.116.029215)
Supplement: Supplemental Material [file supp_g3.116.029215_FigureS1.pdf]

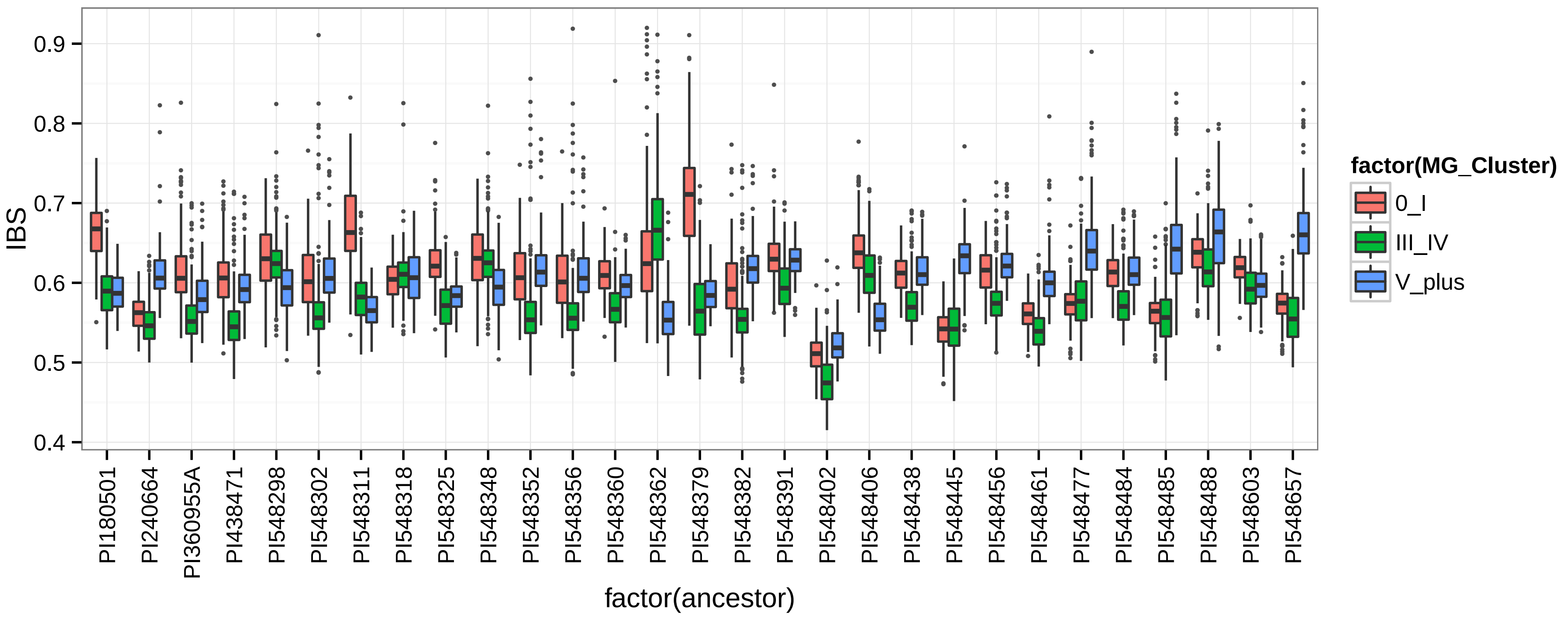

IBS
0.9
0.8
0.7
0.6
0.5
0.4
PI180501
PI240664
PI360955A
PI438471
PI548298
PI548302
PI548311
PI548318
PI548325
PI548348
PI548352
PI548356
PI548360
PI548362
PI548379
PI548382
PI548391
PI548402
PI548406
PI548438
PI548445
PI548456
PI548461
PI548477
PI548484
PI548485
PI548488
PI548603
PI548657
factor(ancestor)
factor(MG_Cluster)
0_I
III_IV
V_plus

Figure S1: Boxplots representing the average IBS between each ancestor and the indicated population.
