# Supplementary material for: Genomic Signatures of North American Soybean Improvement Inform Diversity Enrichment Strategies and Clarify the Impact of Hybridization
Source: G3 (Bethesda). 2016 Jul 7;6(9):2693–705. doi: 10.1534/g3.116.029215 (PMC5015928; doi:10.1534/g3.116.029215)
Supplement: Supplemental Material [file supp_g3.116.029215_FigureS2.pdf]

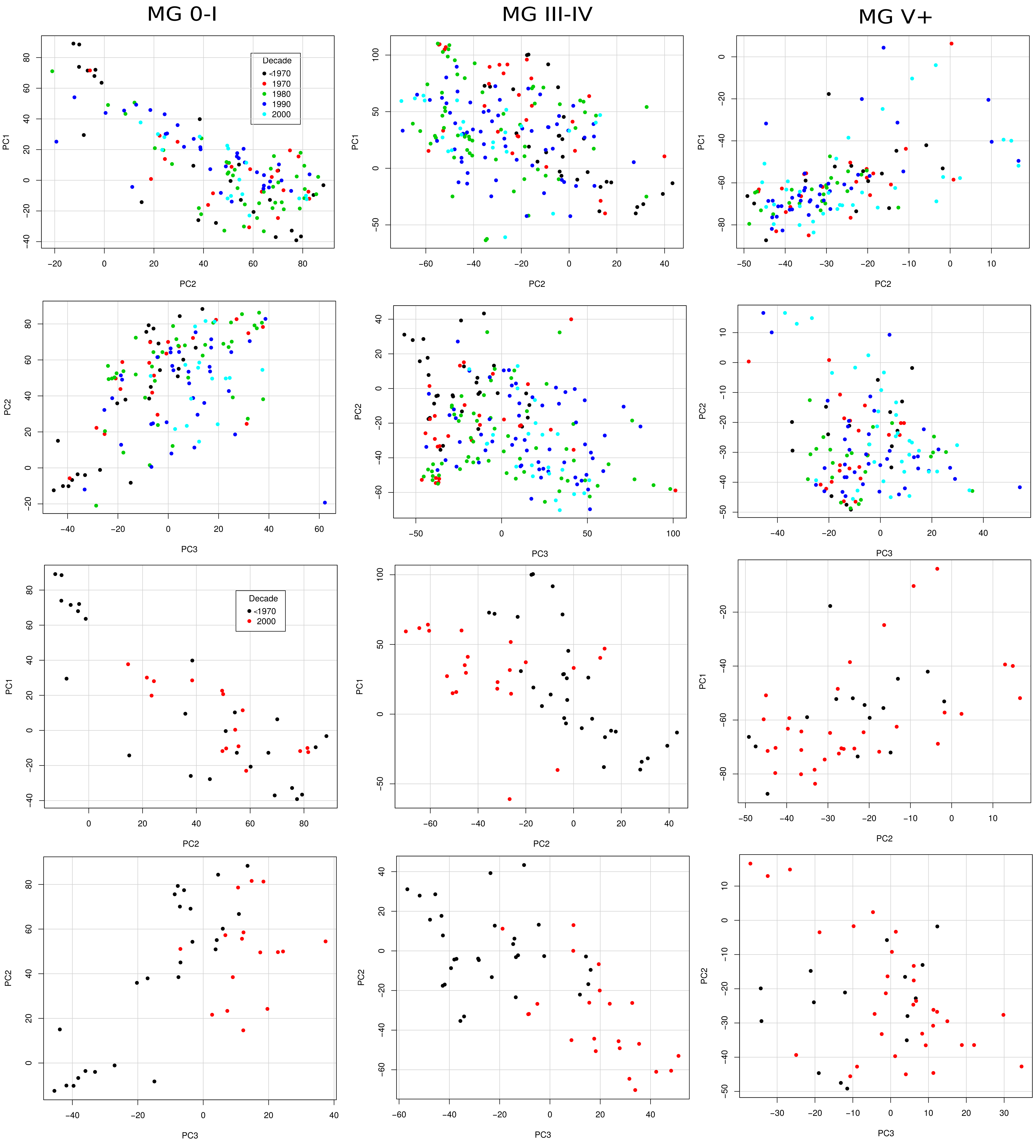

MG 0-I
MG III-IV
MG V+
Decade
<1970
1970
1980
1990
2000
PC1
PC2
PC3

Figure S2: PC plots based on genome-wide marker information across all lines in the analysis. Lines are divided by population as discussed in main text. In the top two panels, lines are color coded based on the era of release. For clarity, only beginning and ending eras are shown in the bottom two panels.
