# Supplementary figures and images for: Genomic Signatures of North American Soybean Improvement Inform Diversity Enrichment Strategies and Clarify the Impact of Hybridization
Source: G3 (Bethesda). 2016 Jul 7;6(9):2693–705. doi: 10.1534/g3.116.029215 (PMC5015928; doi:10.1534/g3.116.029215)

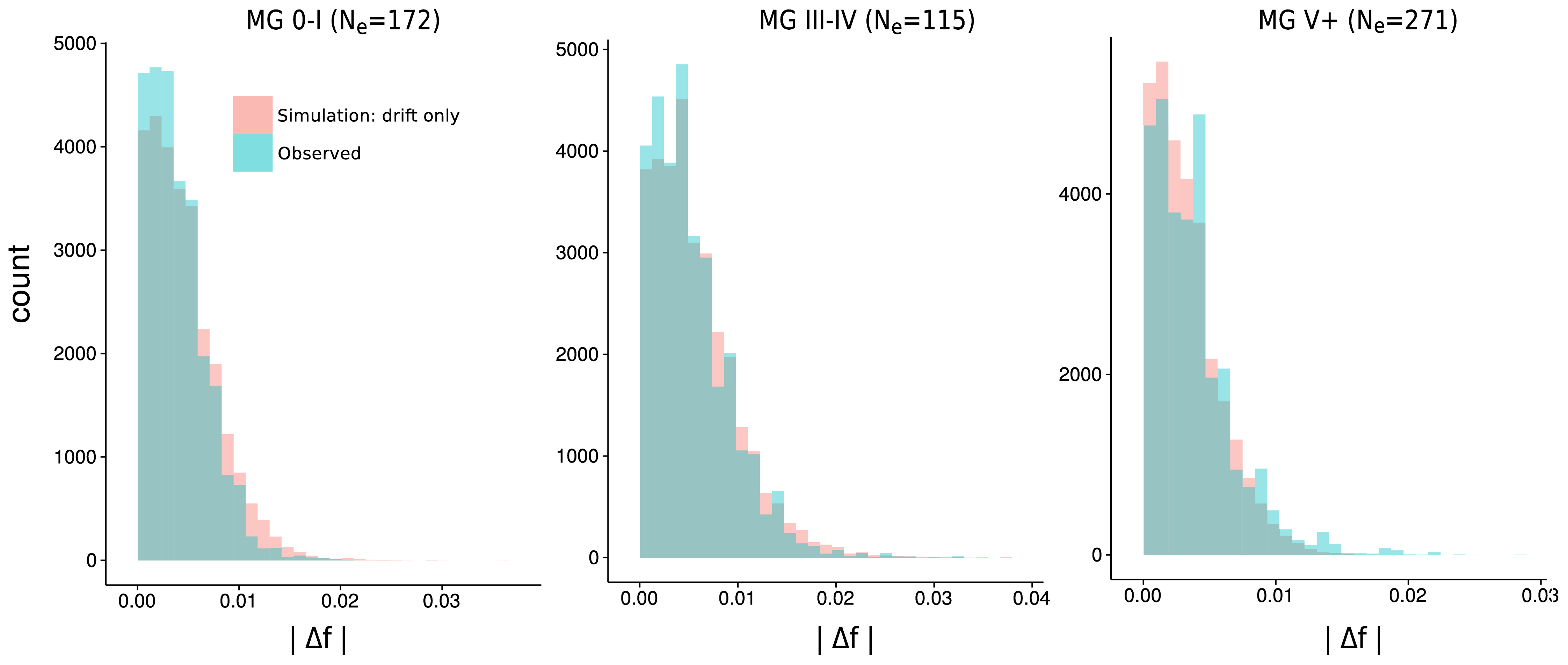

Supplement: Supplemental Material [file supp_g3.116.029215_FigureS3.tif]

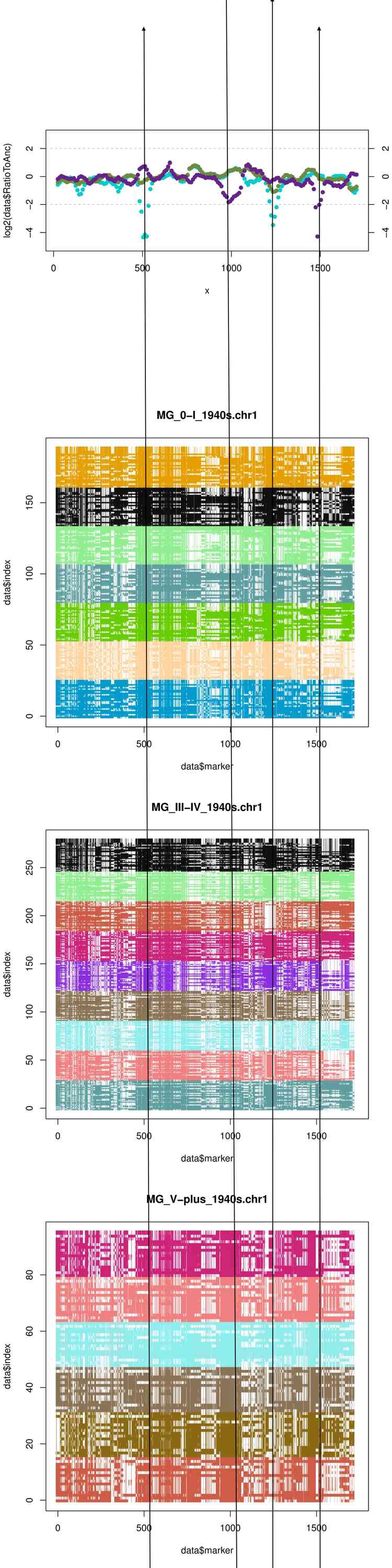

Supplement: Supplemental Material [file supp_g3.116.029215_FileS1.zip › chrom1.jpg]

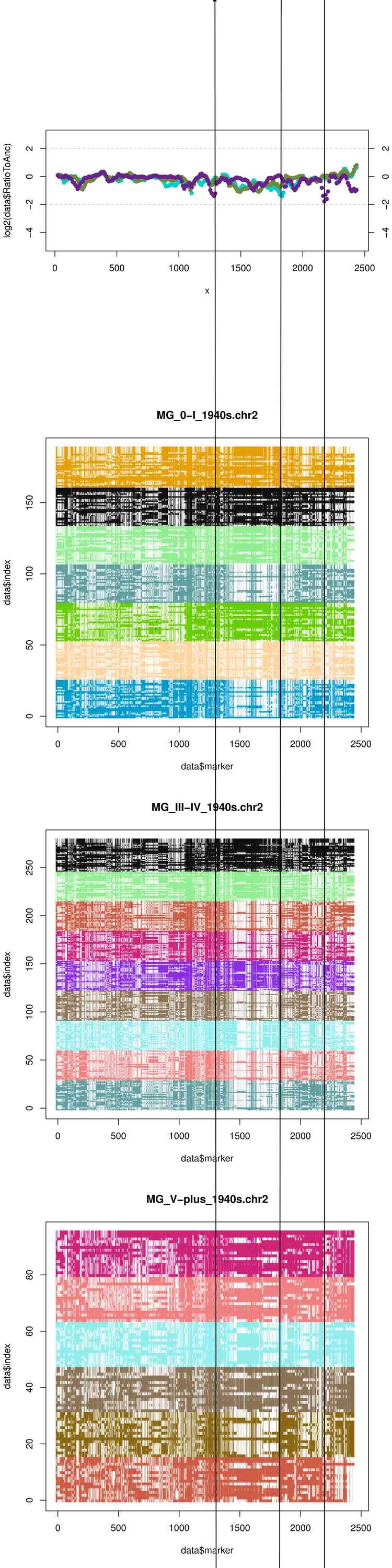

Supplement: Supplemental Material [file supp_g3.116.029215_FileS1.zip › chrom2.jpg]

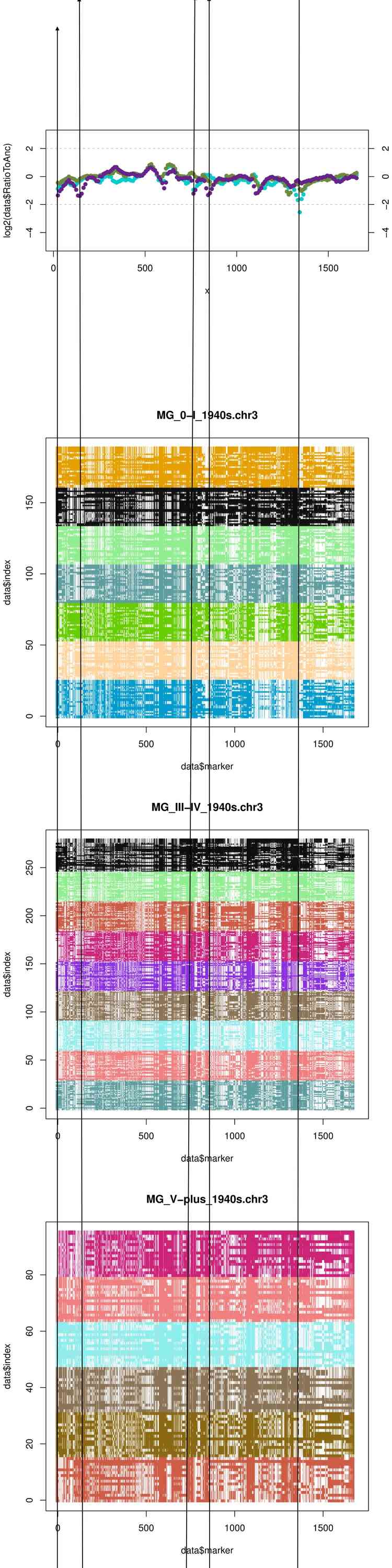

Supplement: Supplemental Material [file supp_g3.116.029215_FileS1.zip › chrom3.jpg]

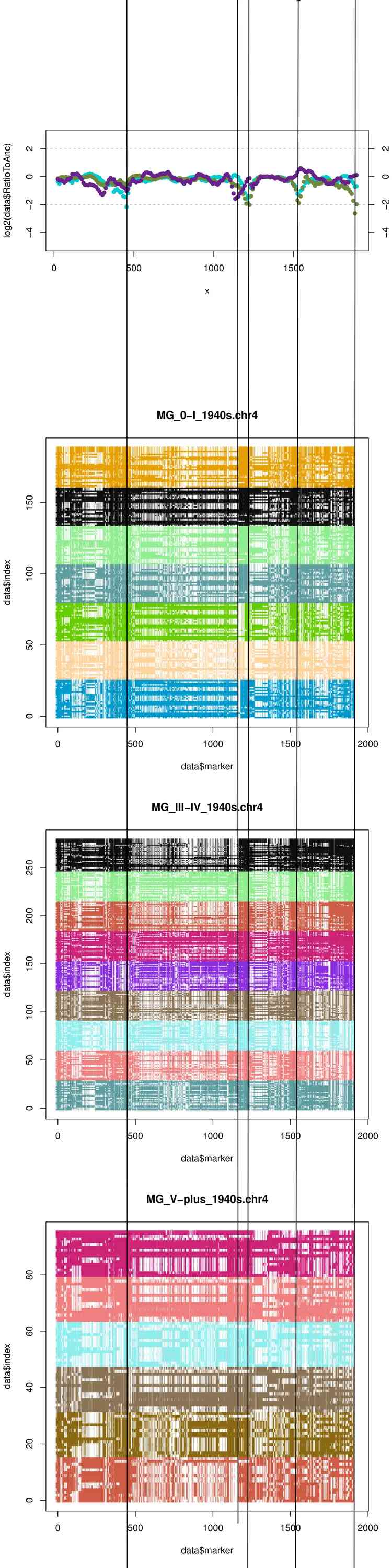

Supplement: Supplemental Material [file supp_g3.116.029215_FileS1.zip › chrom4.jpg]

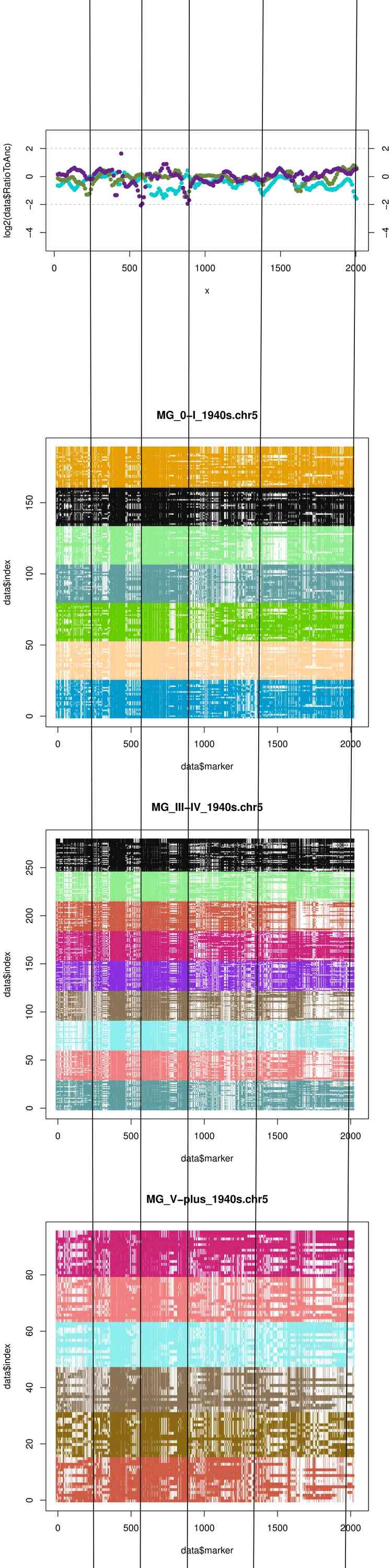

Supplement: Supplemental Material [file supp_g3.116.029215_FileS1.zip › chrom5.jpg]

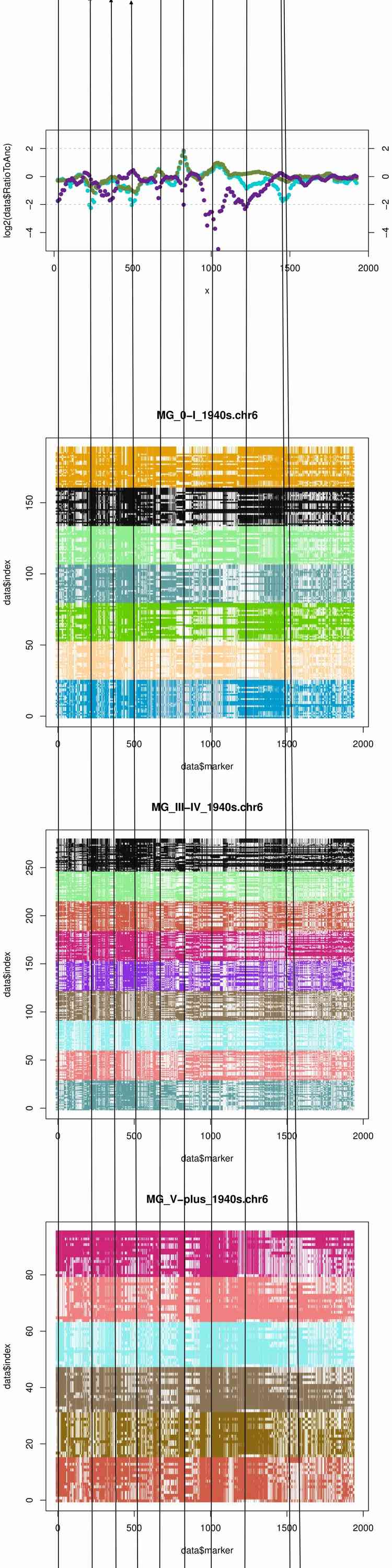

Supplement: Supplemental Material [file supp_g3.116.029215_FileS1.zip › chrom6.jpg]

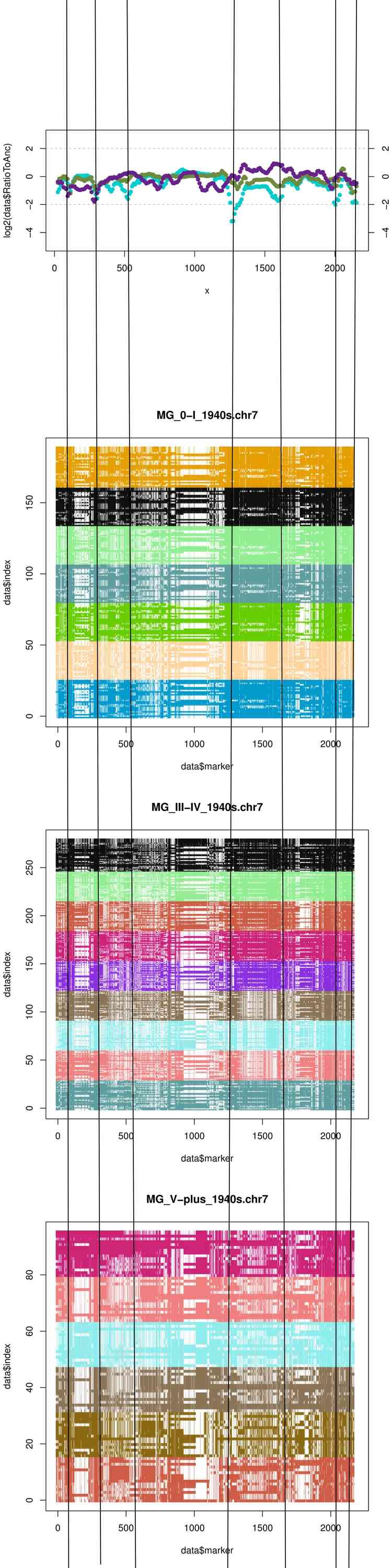

Supplement: Supplemental Material [file supp_g3.116.029215_FileS1.zip › chrom7.jpg]

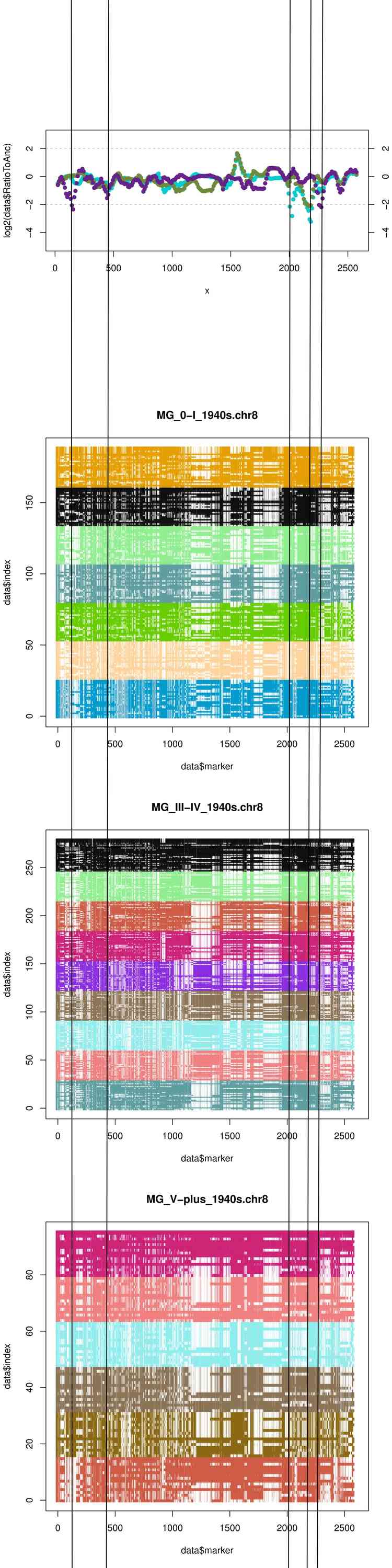

Supplement: Supplemental Material [file supp_g3.116.029215_FileS1.zip › chrom8.jpg]

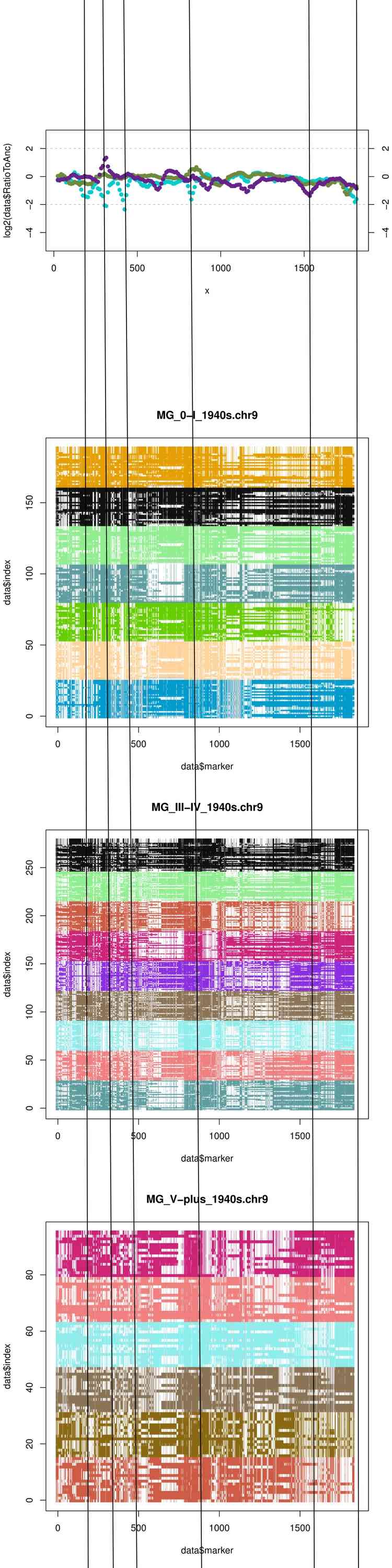

Supplement: Supplemental Material [file supp_g3.116.029215_FileS1.zip › chrom9.jpg]

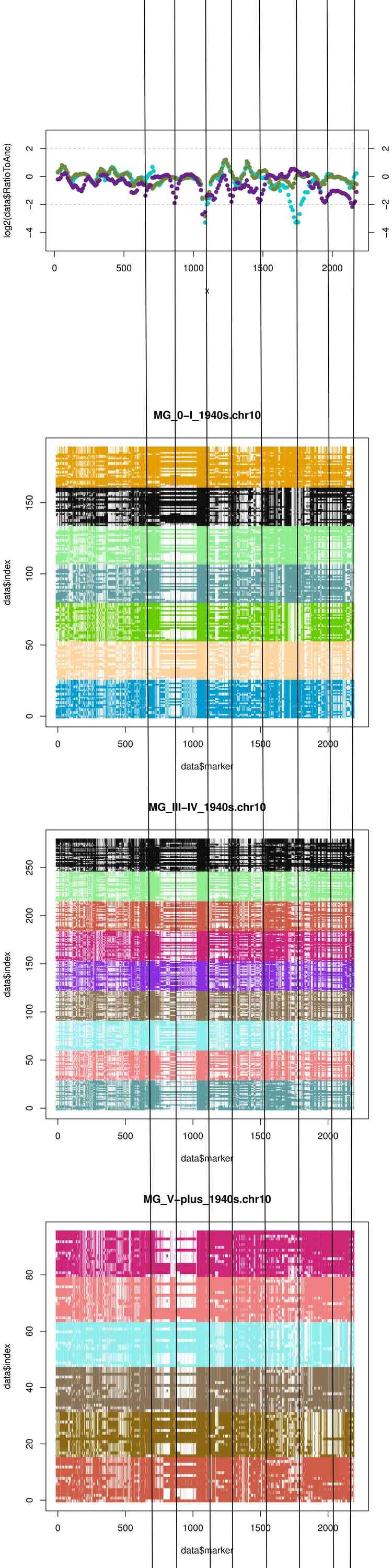

Supplement: Supplemental Material [file supp_g3.116.029215_FileS1.zip › chrom10.jpg]

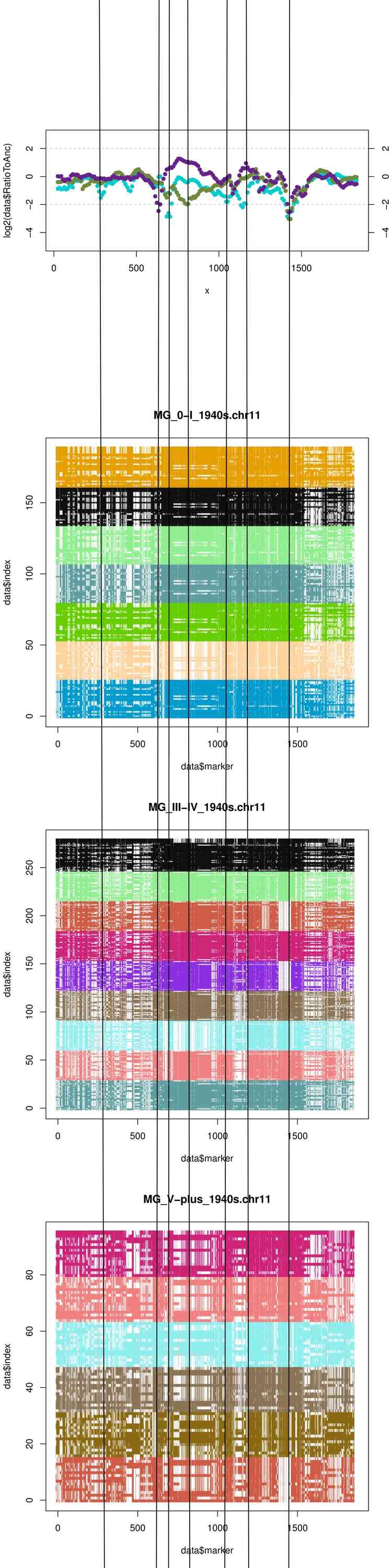

Supplement: Supplemental Material [file supp_g3.116.029215_FileS1.zip › chrom11.jpg]

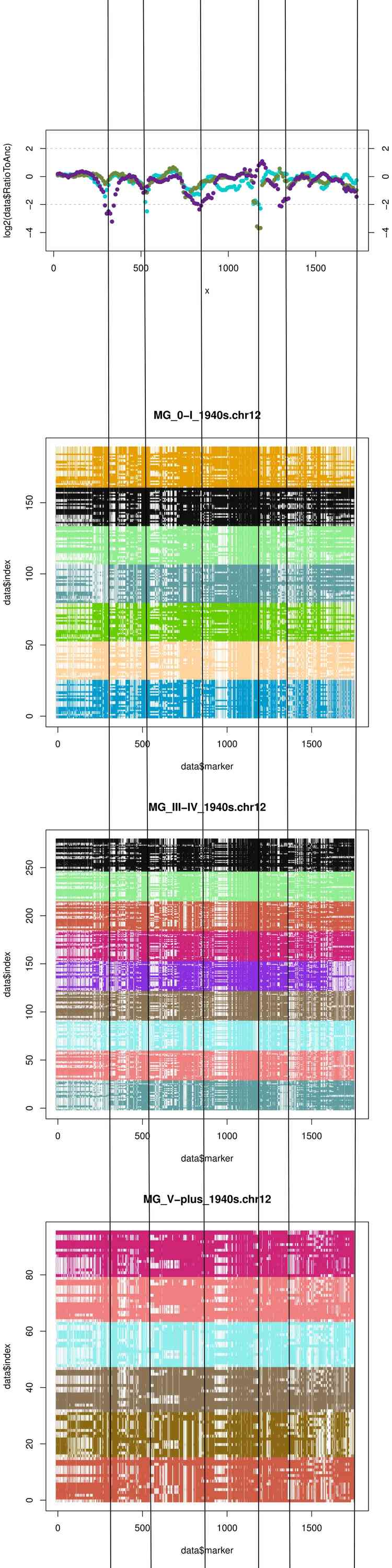

Supplement: Supplemental Material [file supp_g3.116.029215_FileS1.zip › chrom12.jpg]

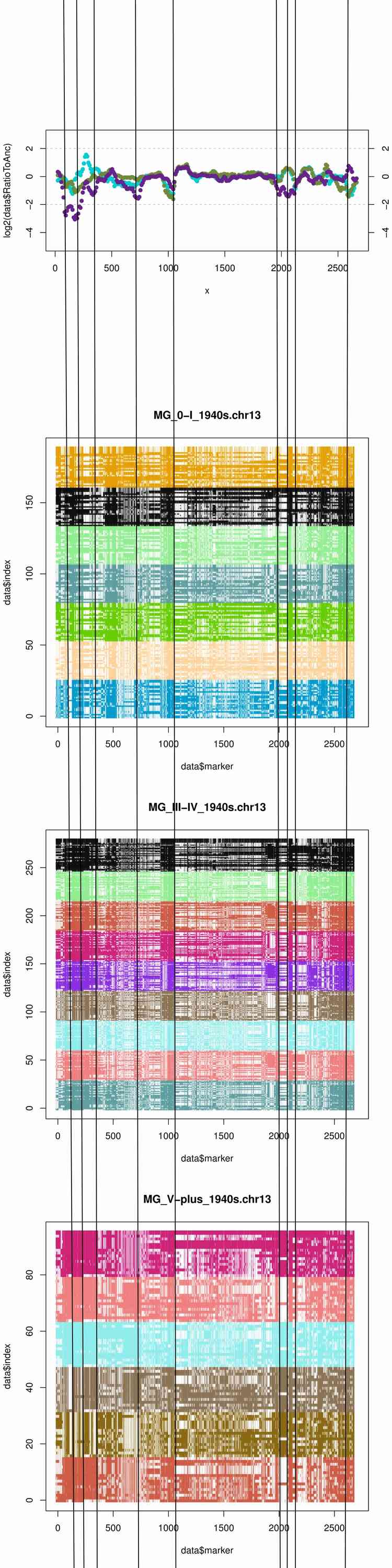

Supplement: Supplemental Material [file supp_g3.116.029215_FileS1.zip › chrom13.jpg]

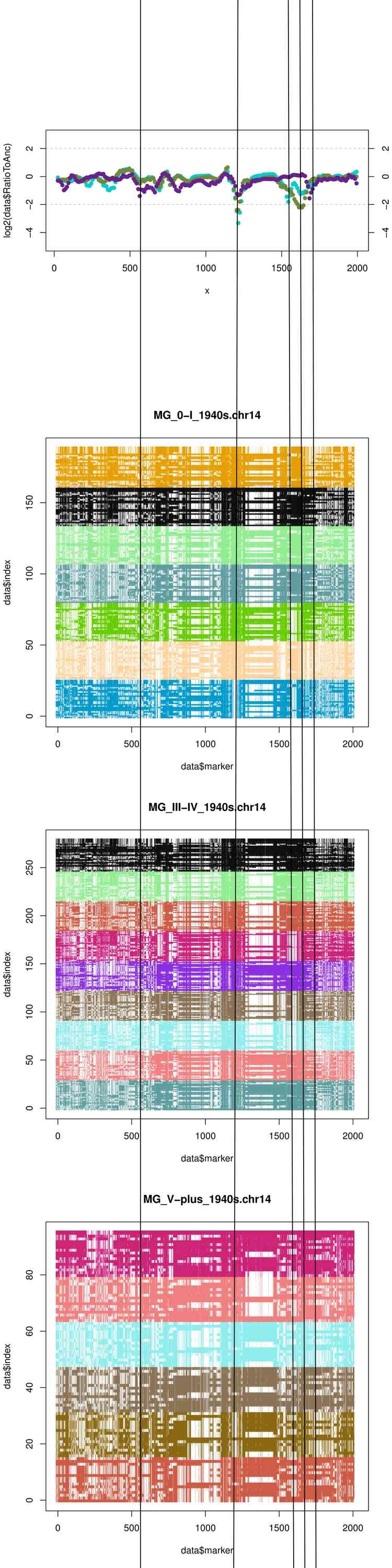

Supplement: Supplemental Material [file supp_g3.116.029215_FileS1.zip › chrom14.jpg]

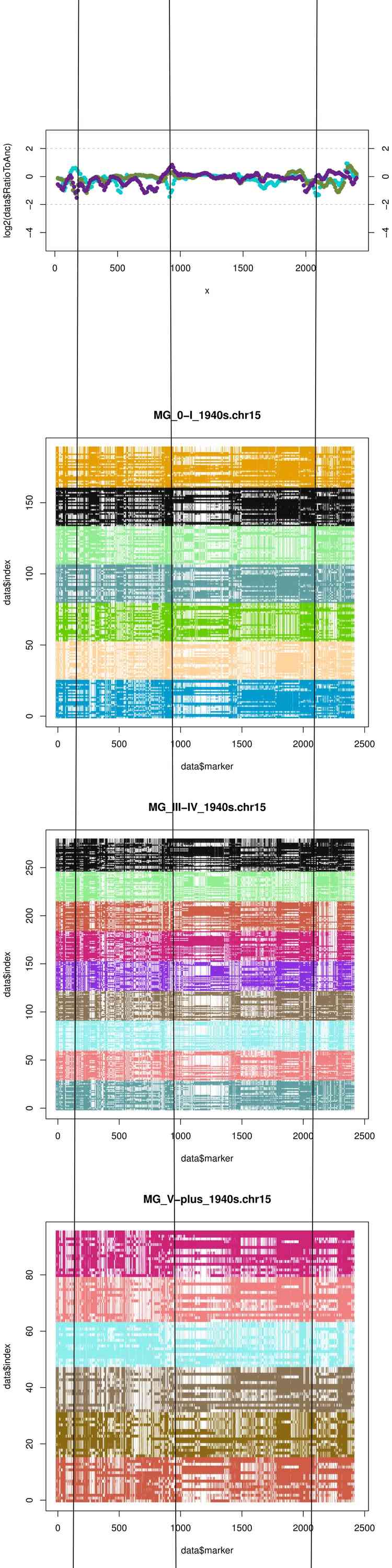

Supplement: Supplemental Material [file supp_g3.116.029215_FileS1.zip › chrom15.jpg]

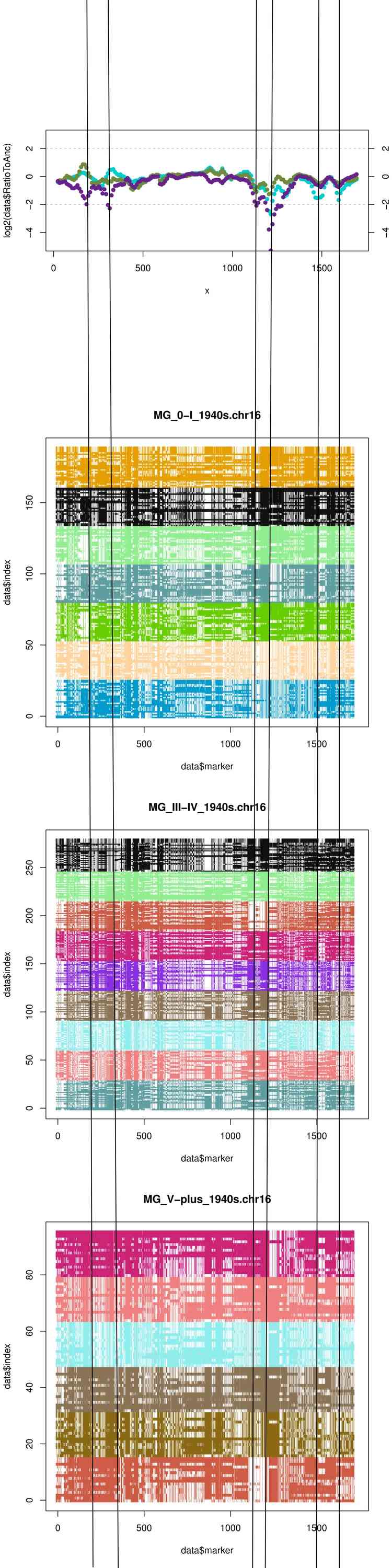

Supplement: Supplemental Material [file supp_g3.116.029215_FileS1.zip › chrom16.jpg]

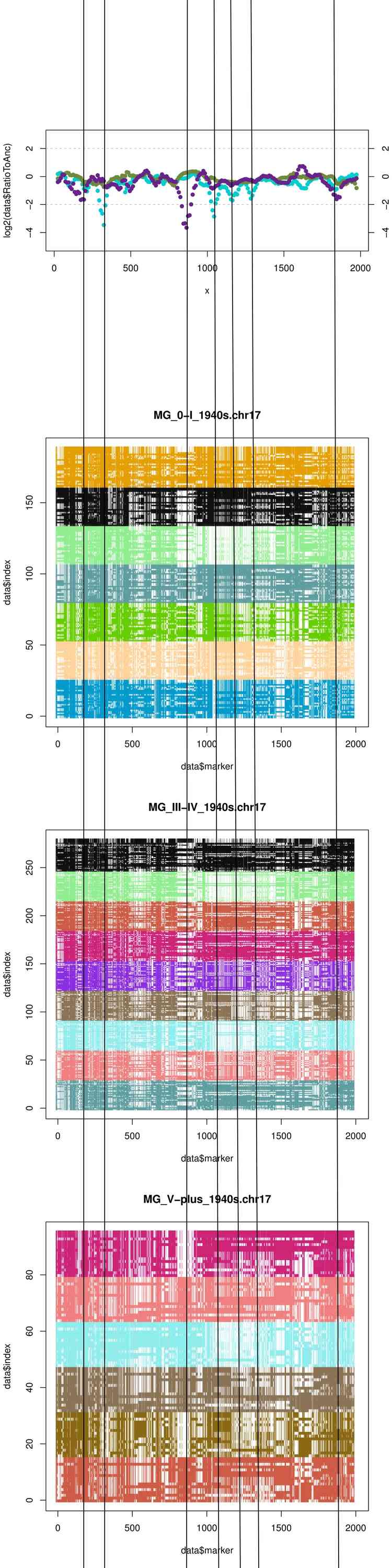

Supplement: Supplemental Material [file supp_g3.116.029215_FileS1.zip › chrom17.jpg]

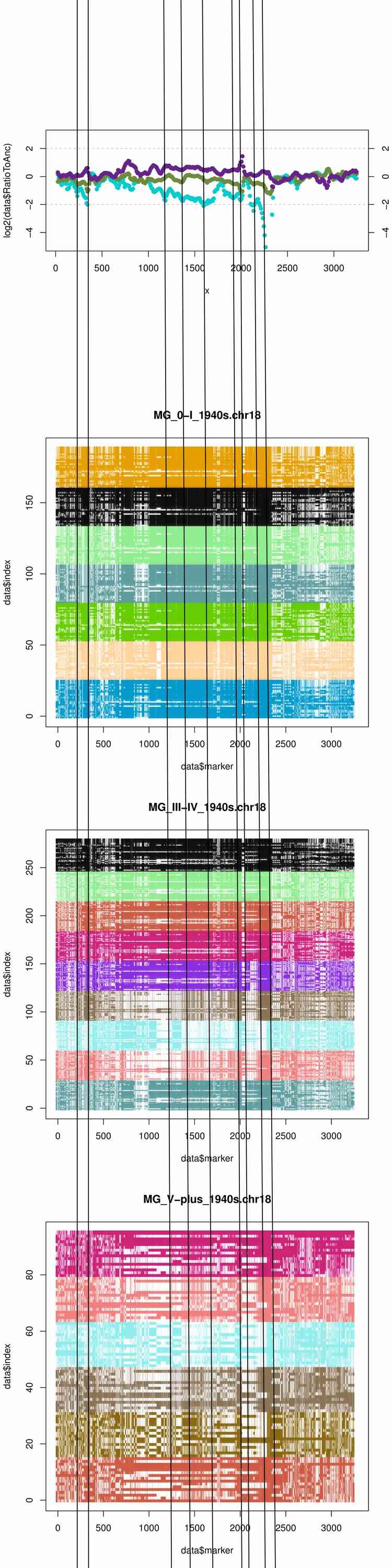

Supplement: Supplemental Material [file supp_g3.116.029215_FileS1.zip › chrom18.jpg]

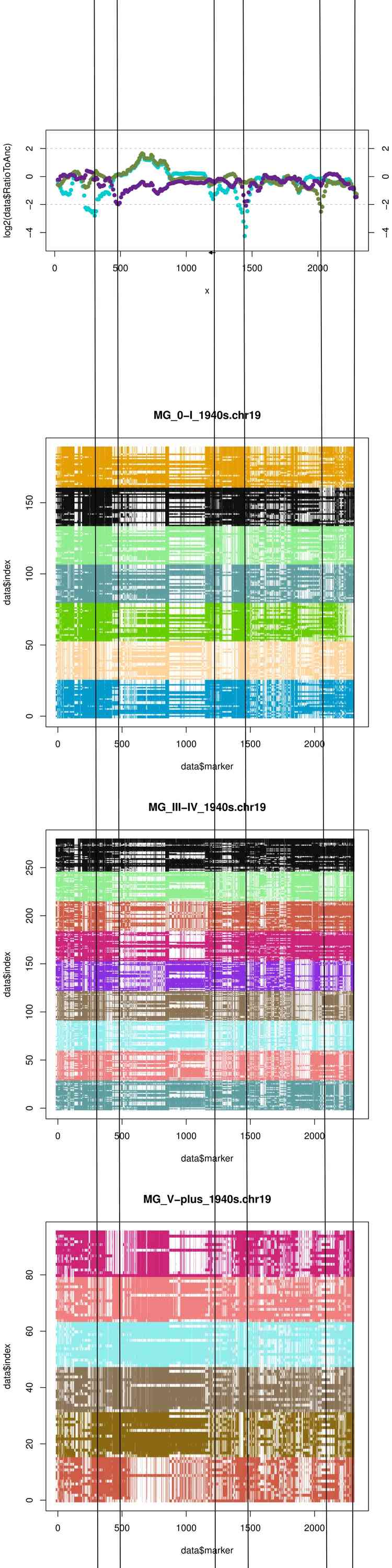

Supplement: Supplemental Material [file supp_g3.116.029215_FileS1.zip › chrom19.jpg]

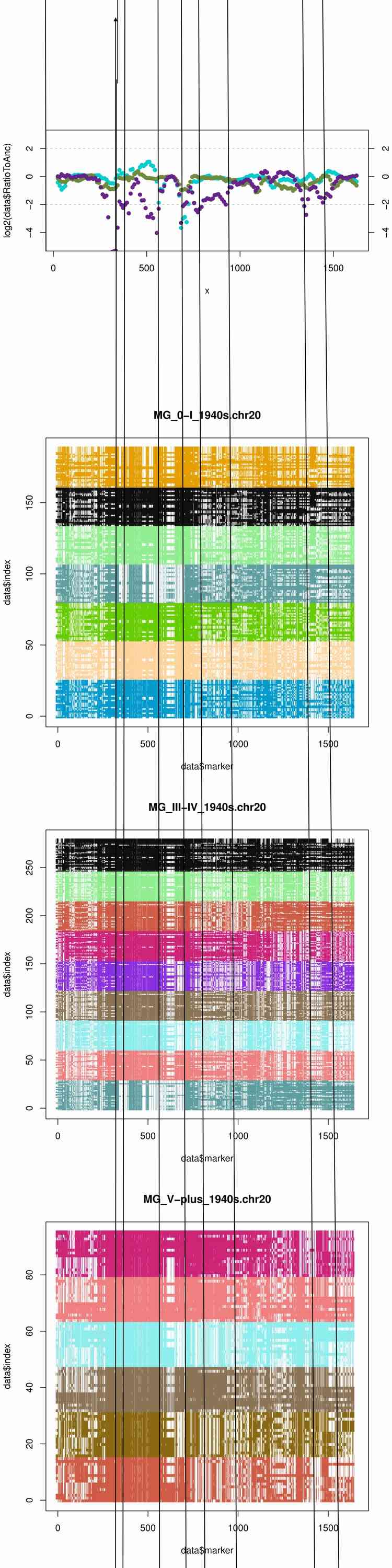

Supplement: Supplemental Material [file supp_g3.116.029215_FileS1.zip › chrom20.jpg]

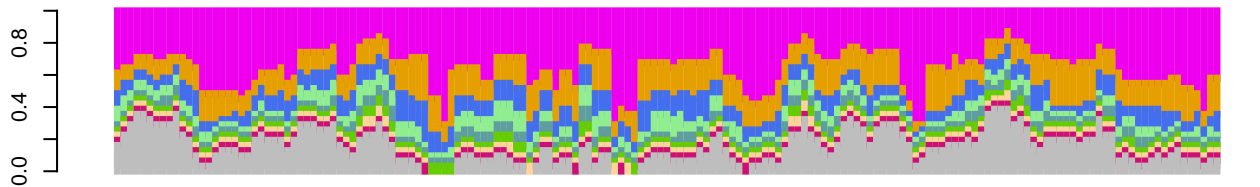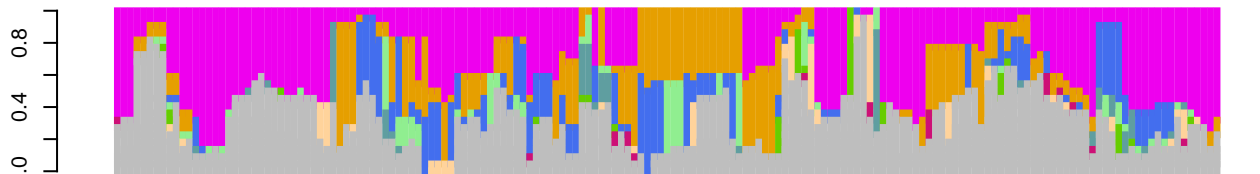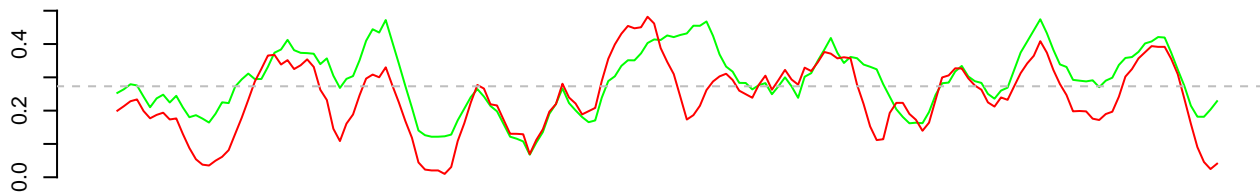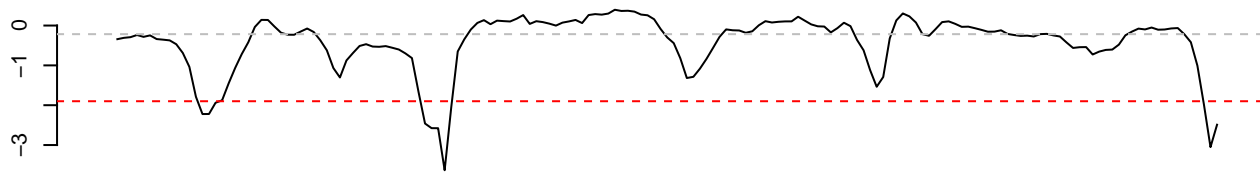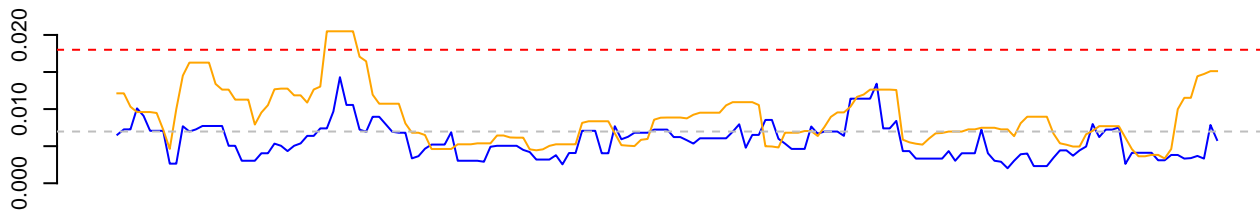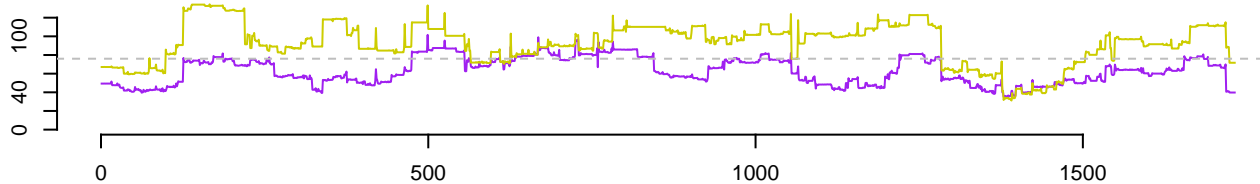

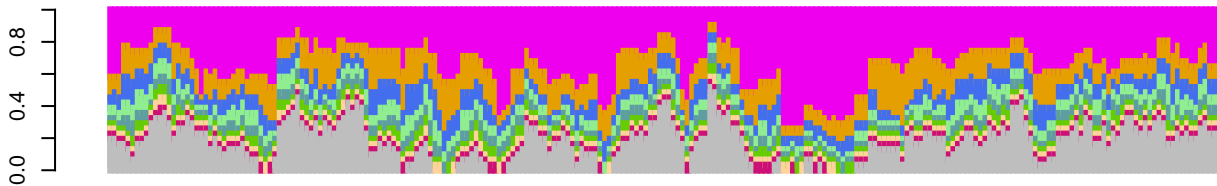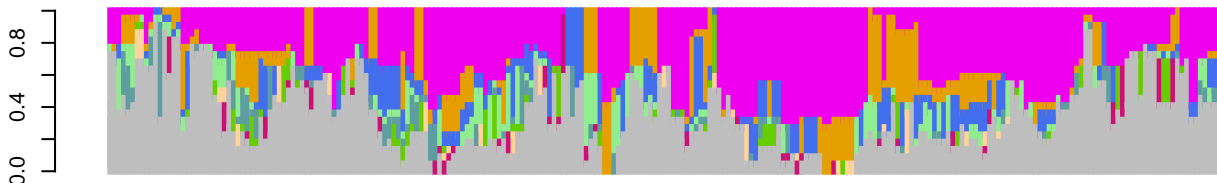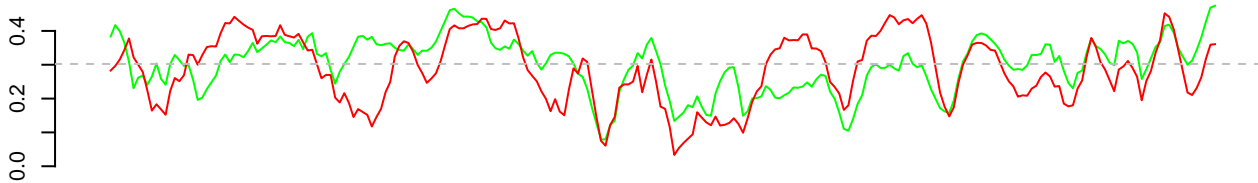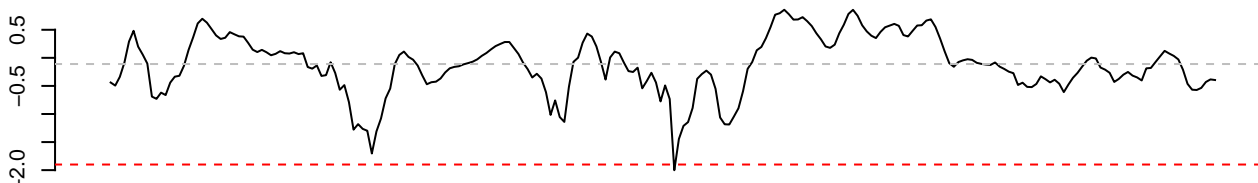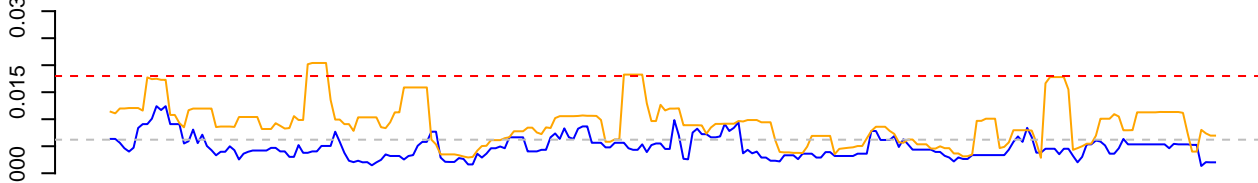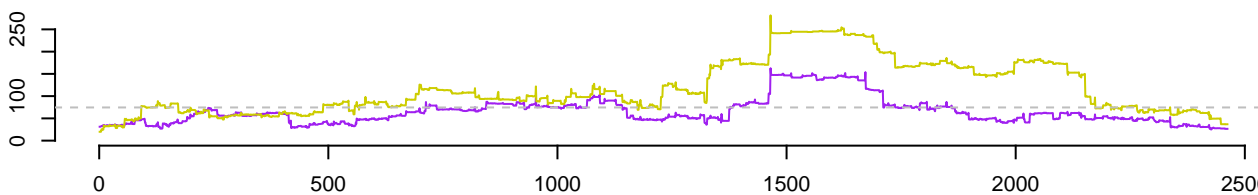

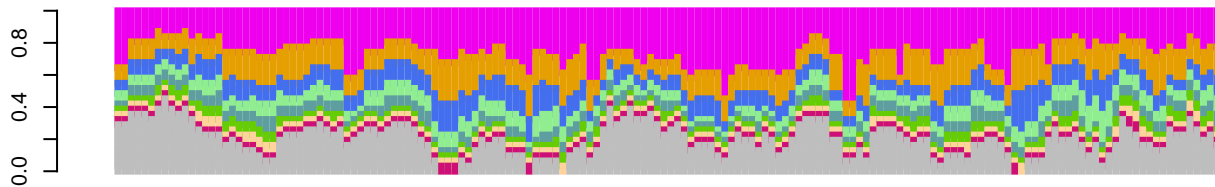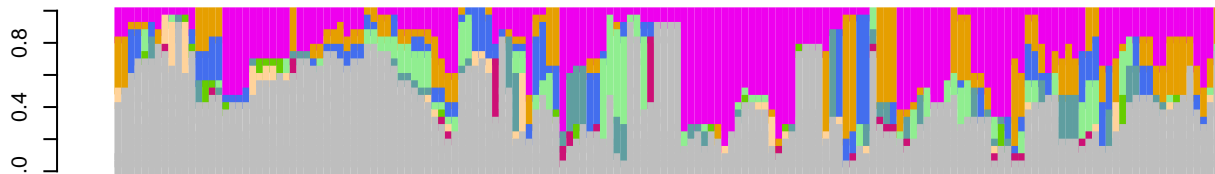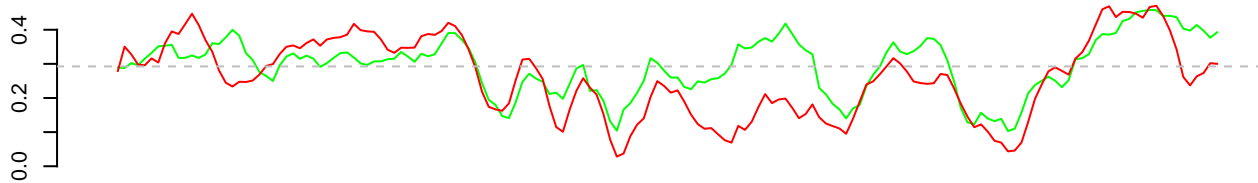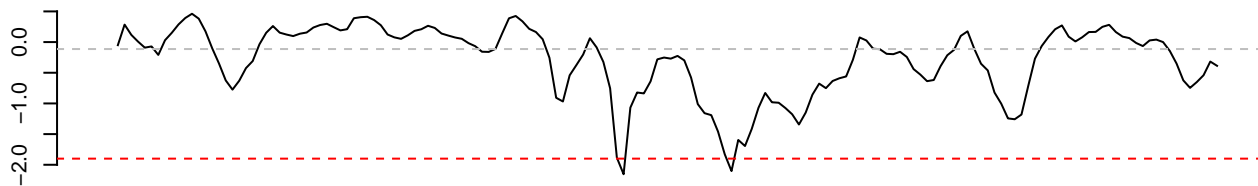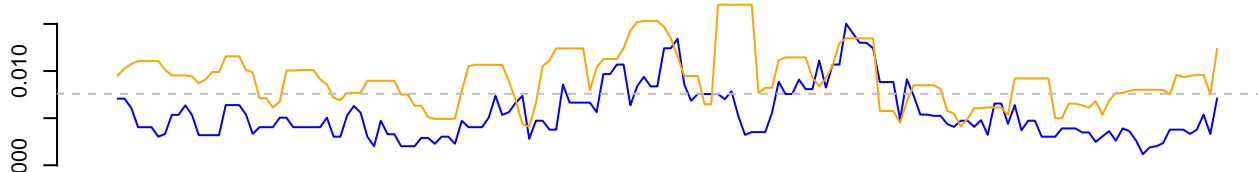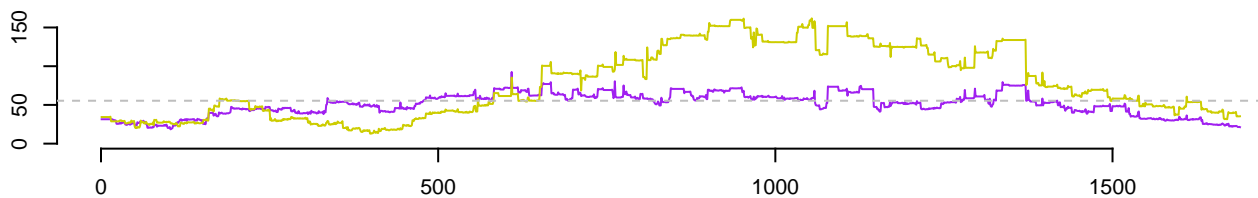

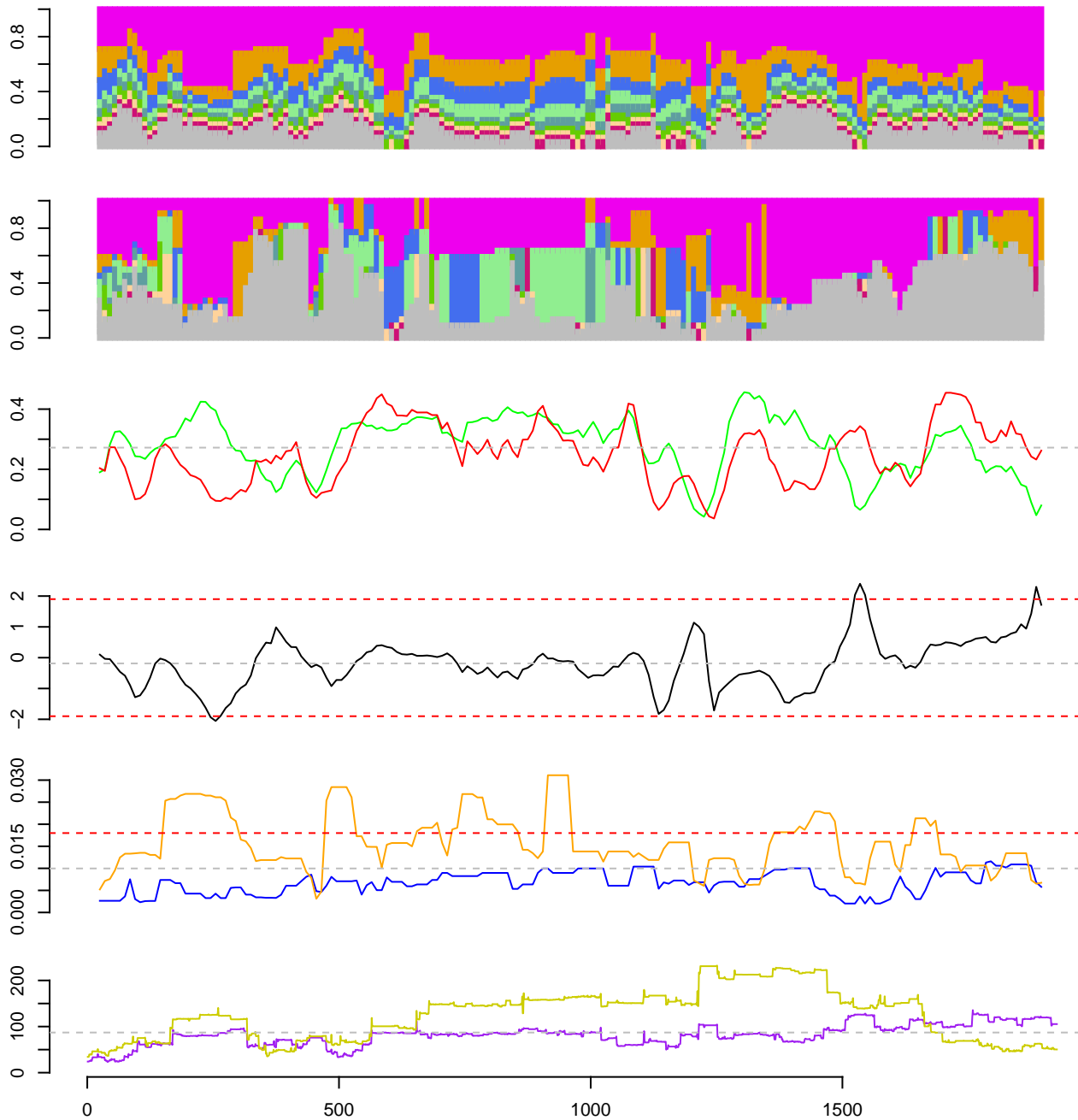

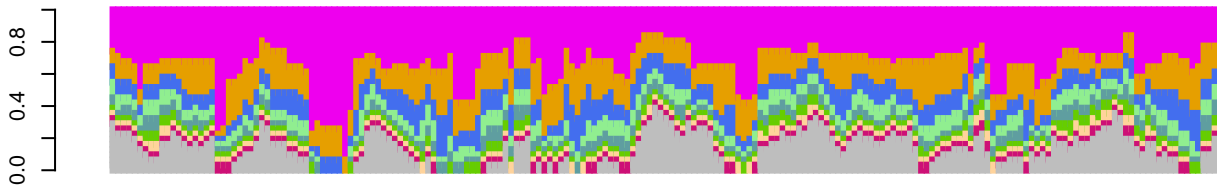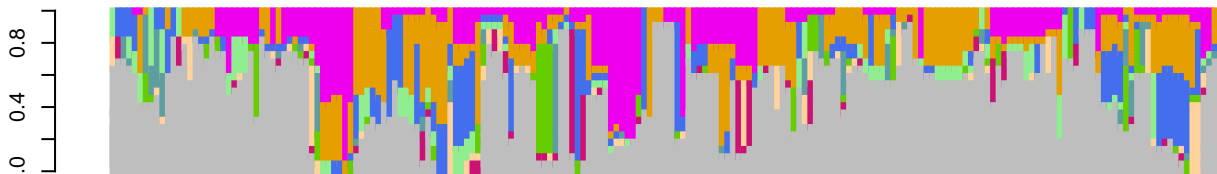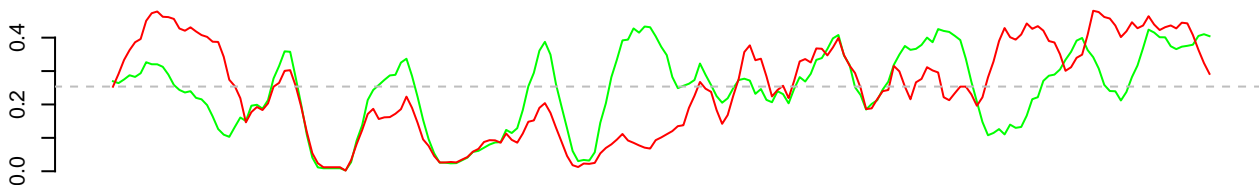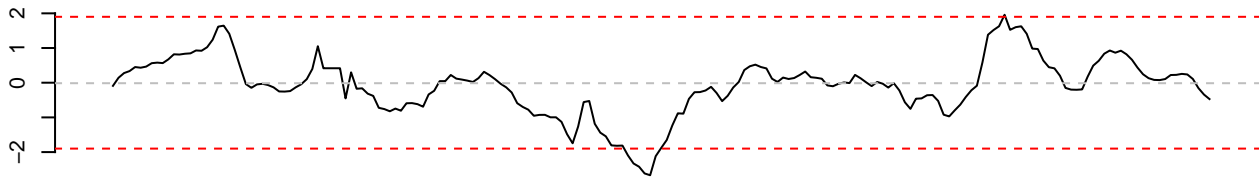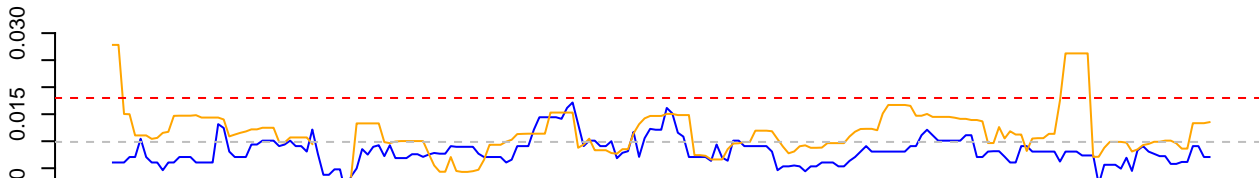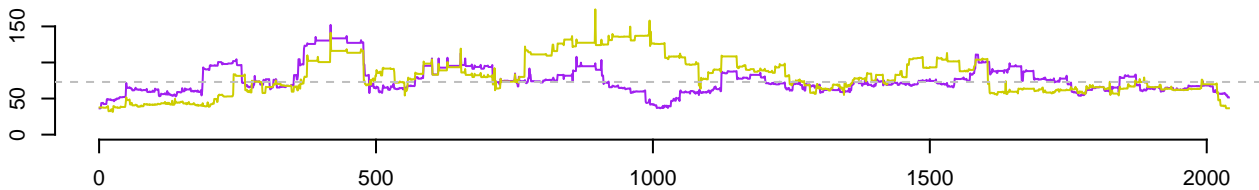

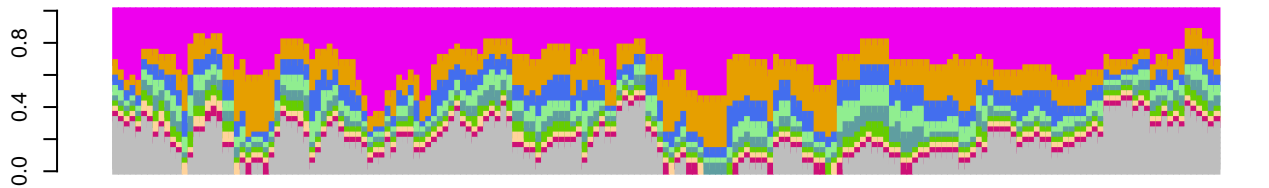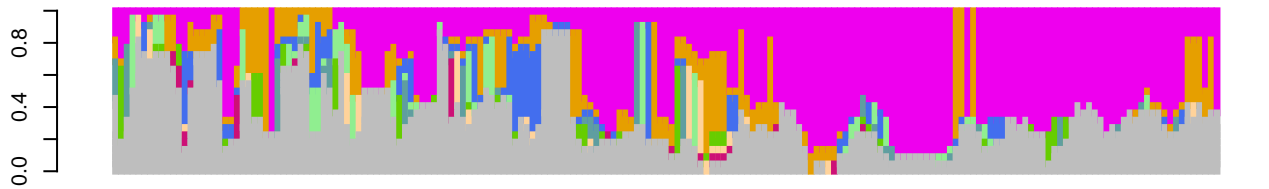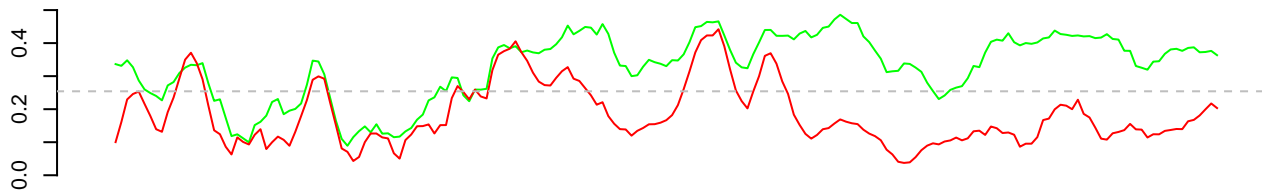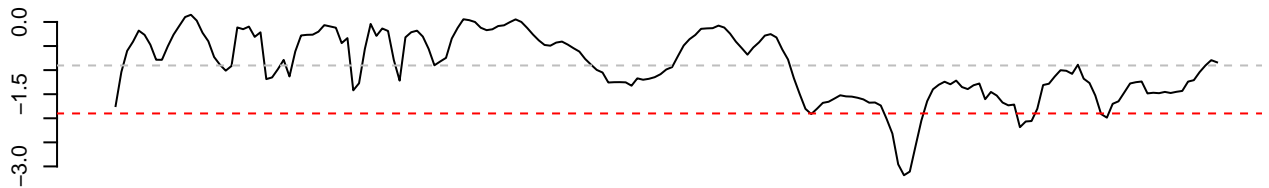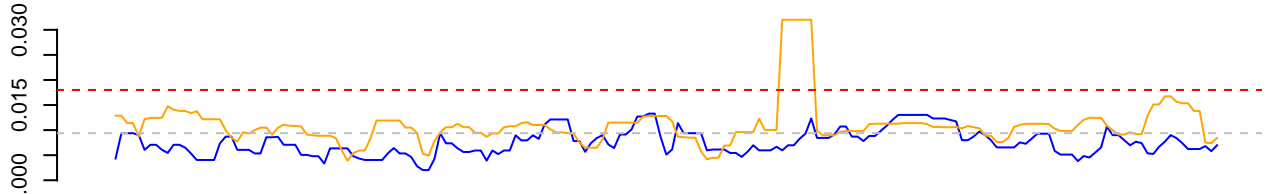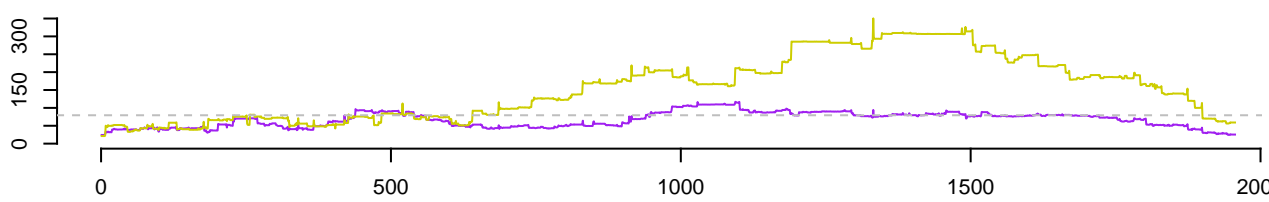

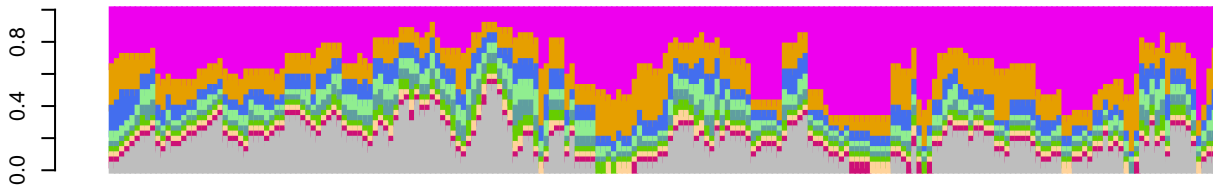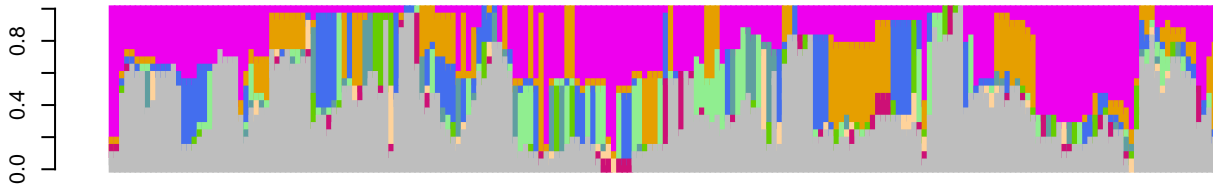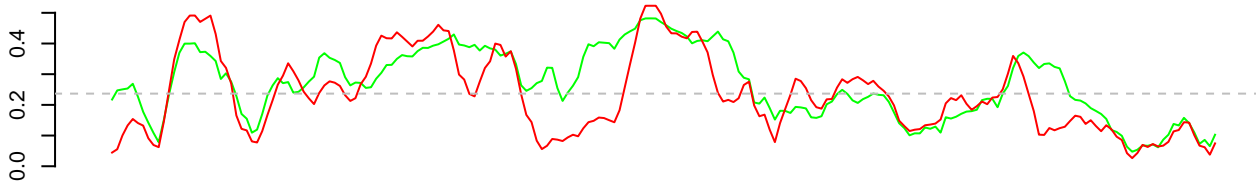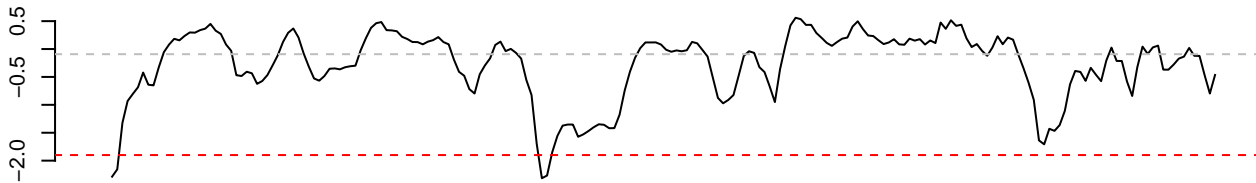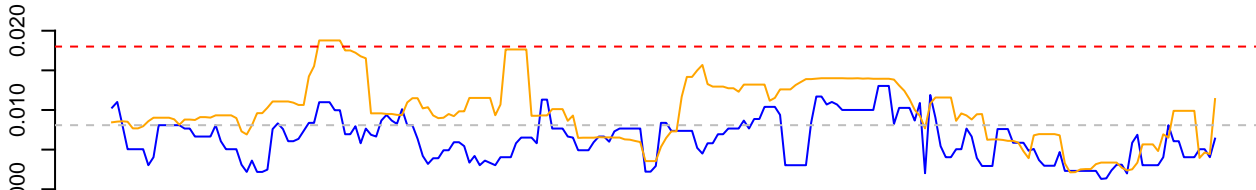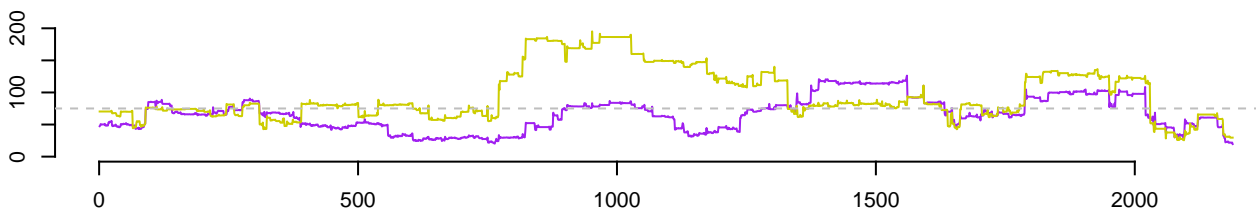

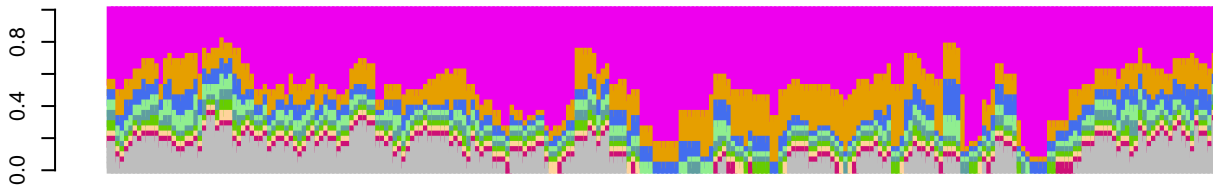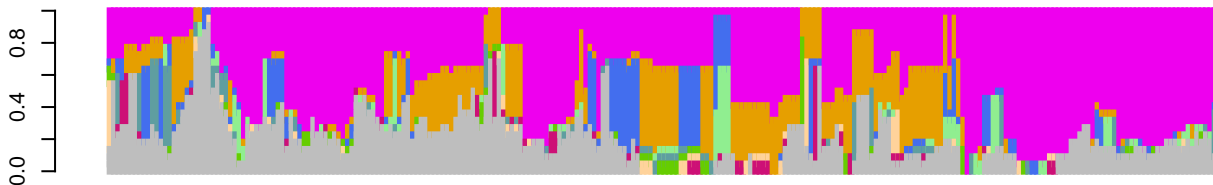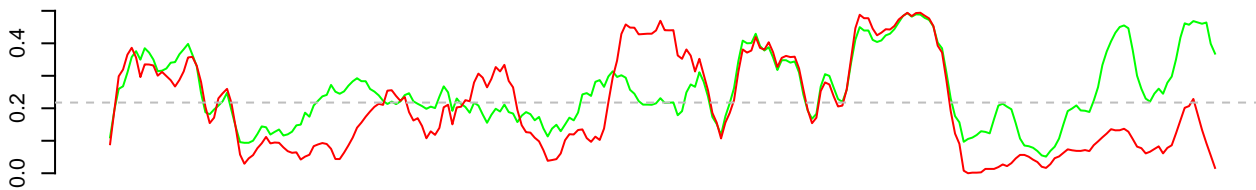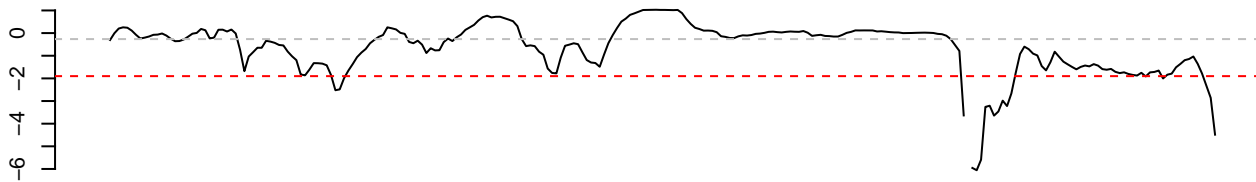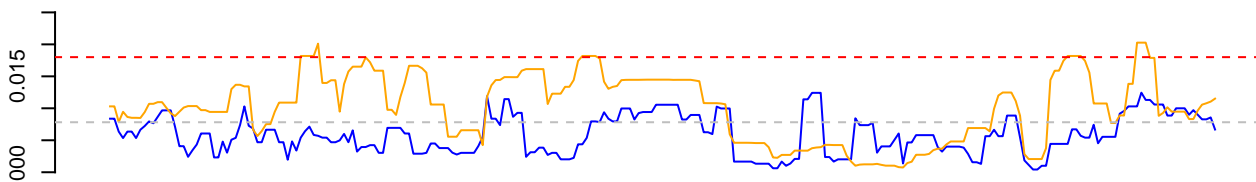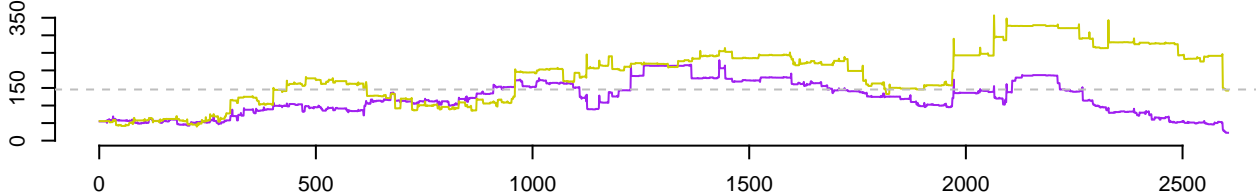

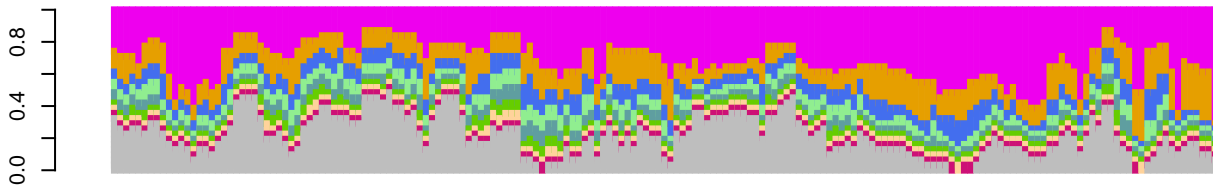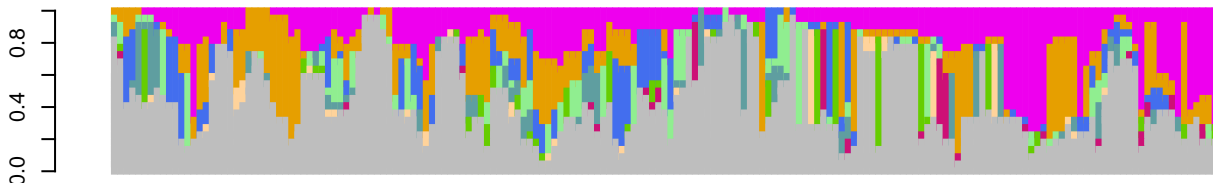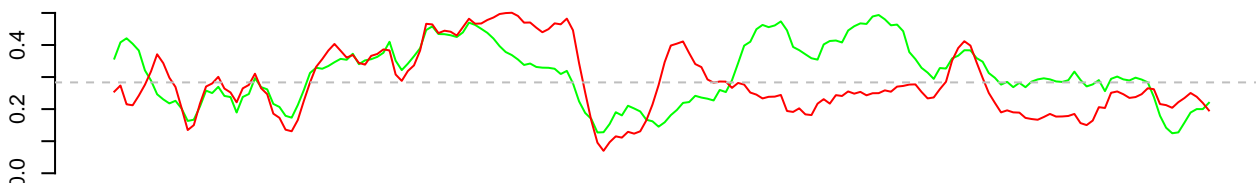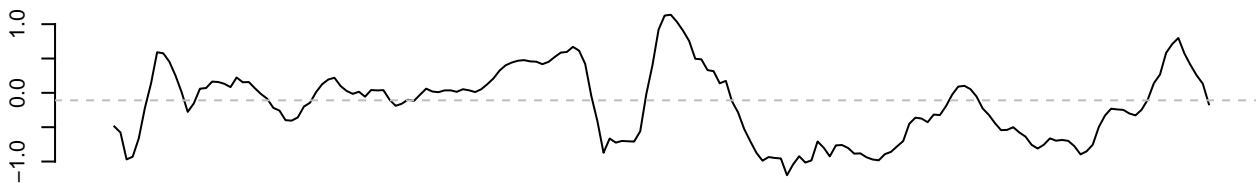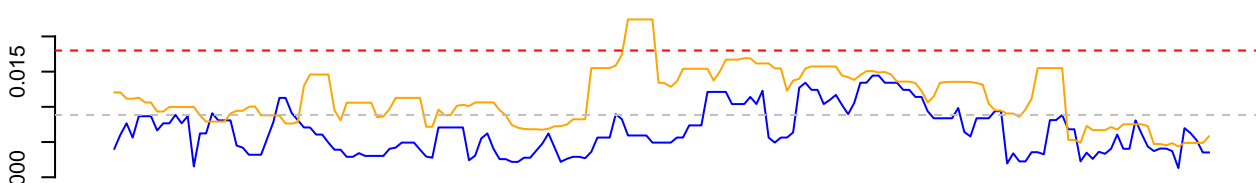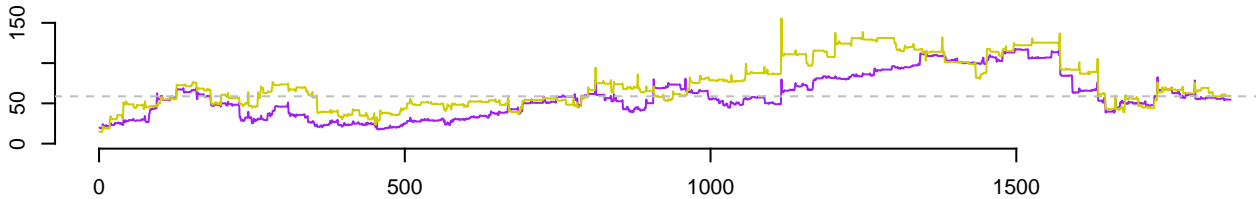

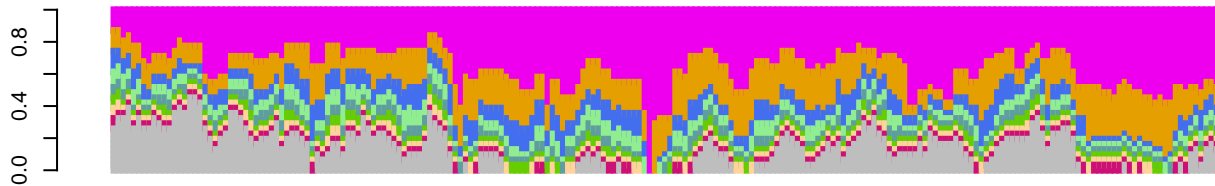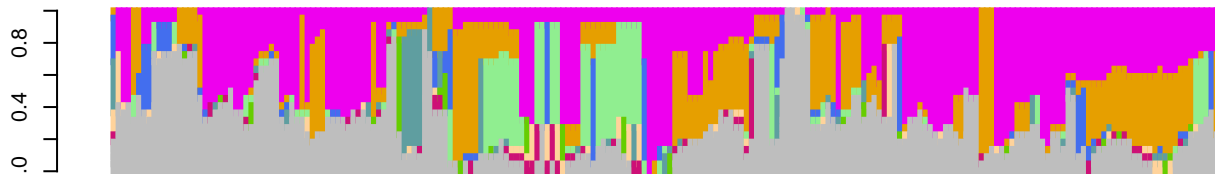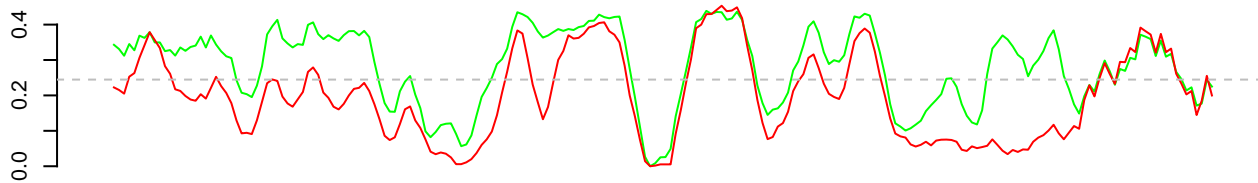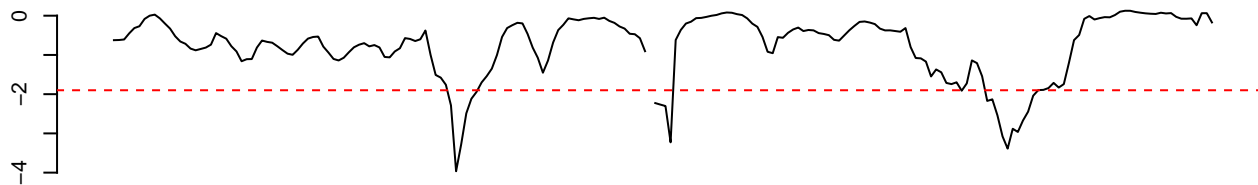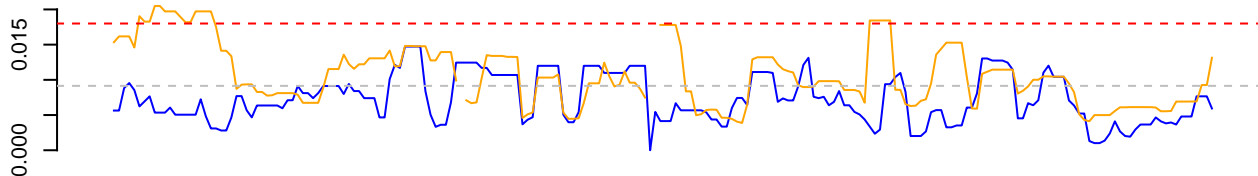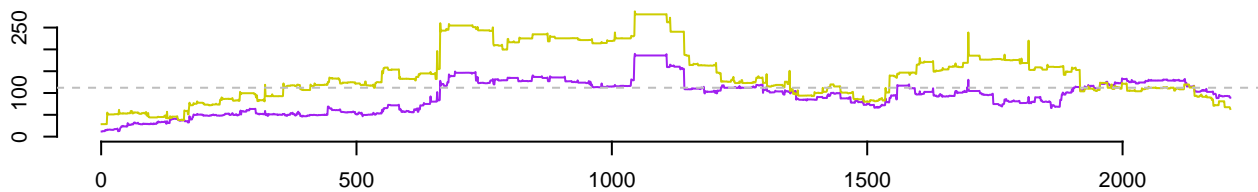

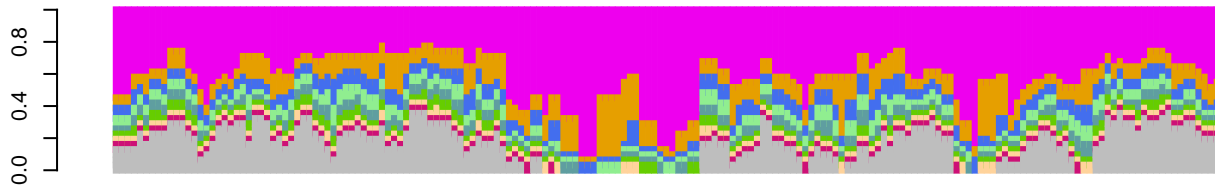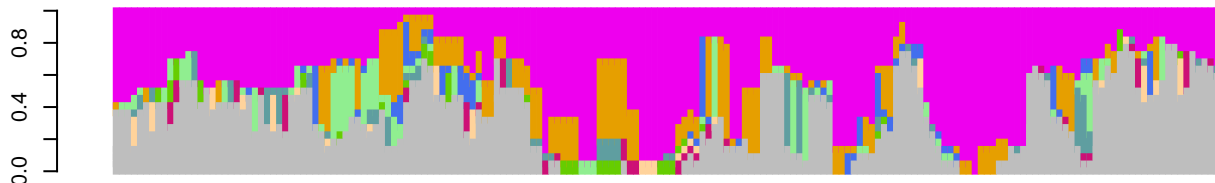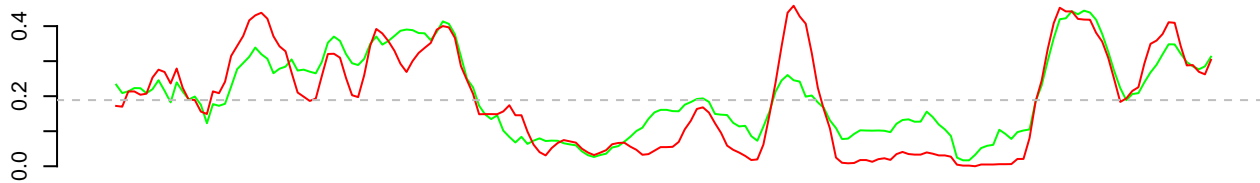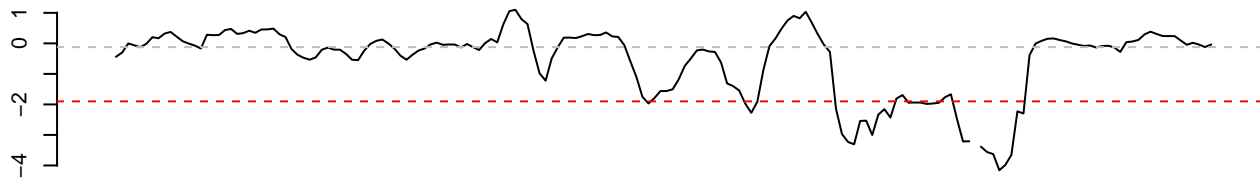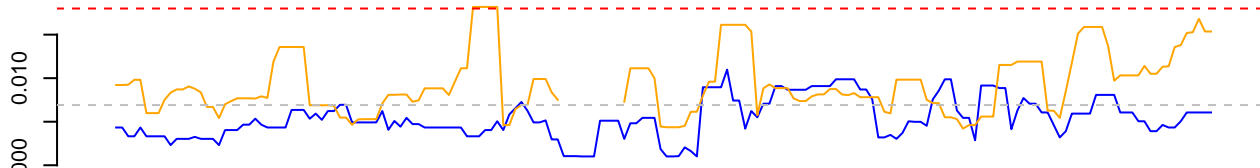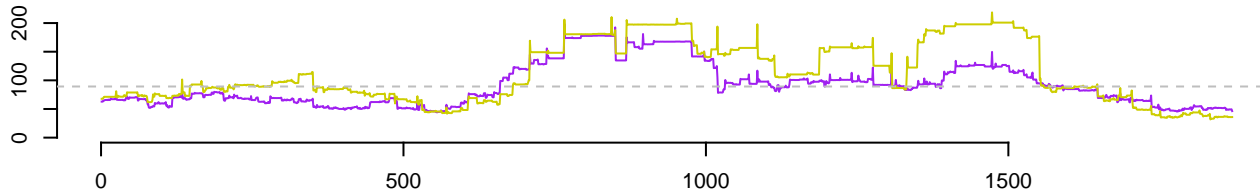

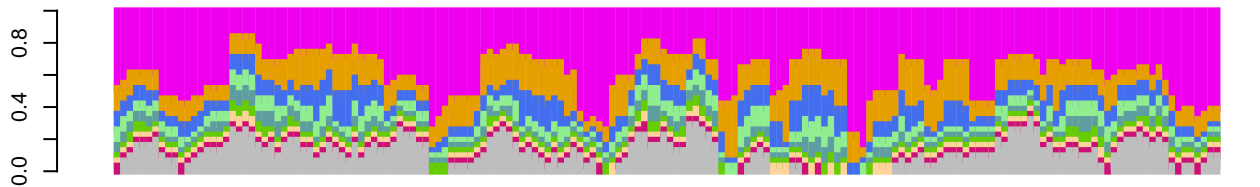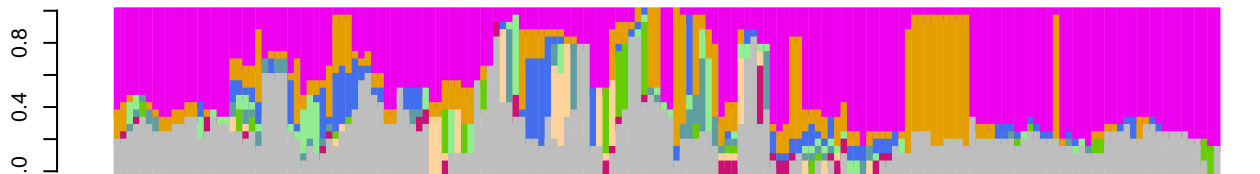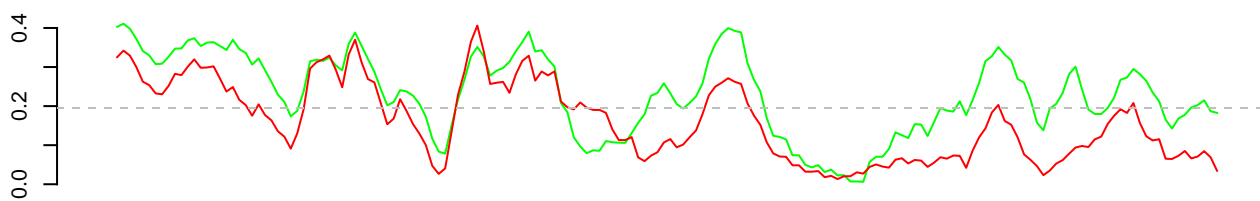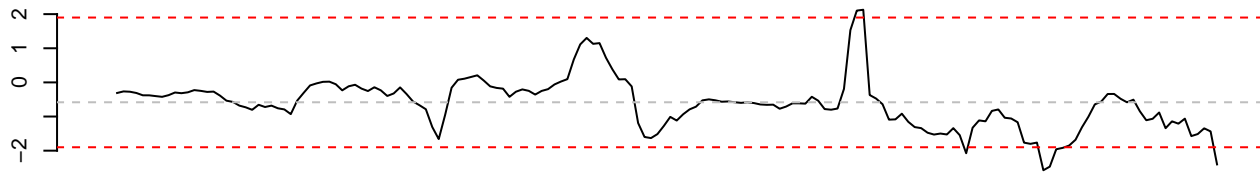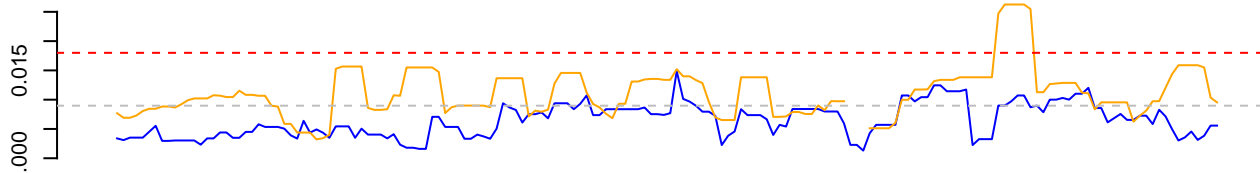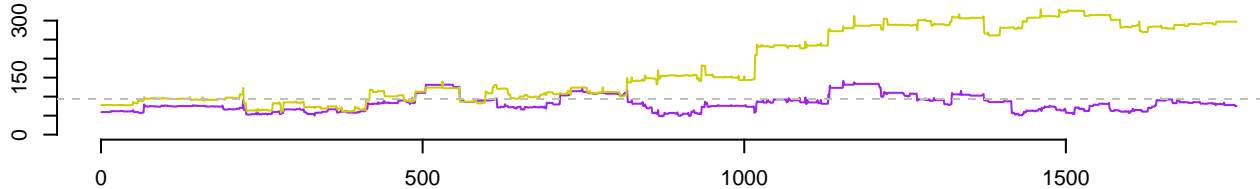

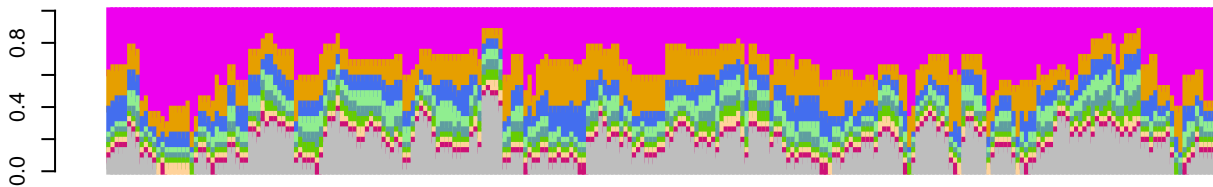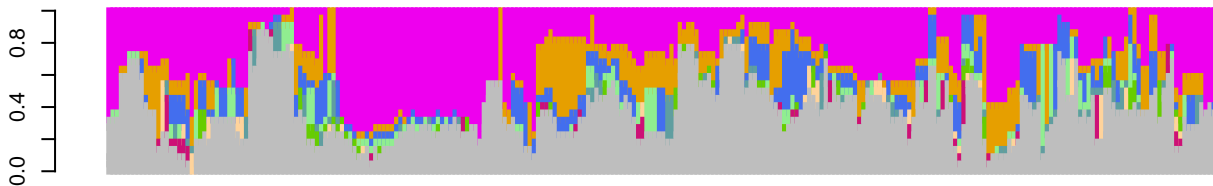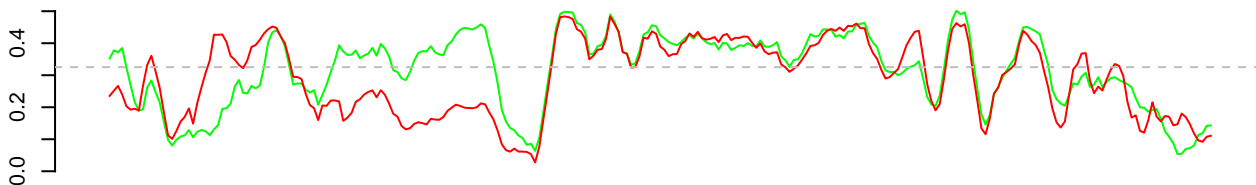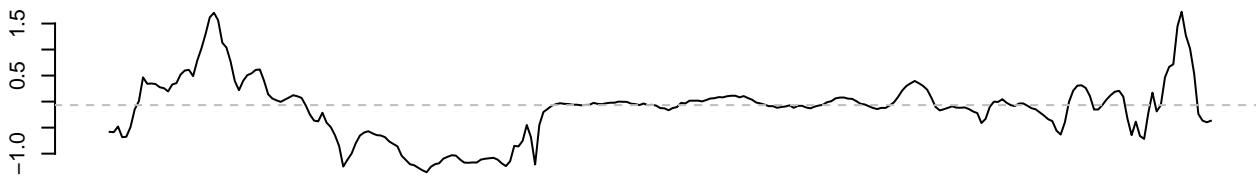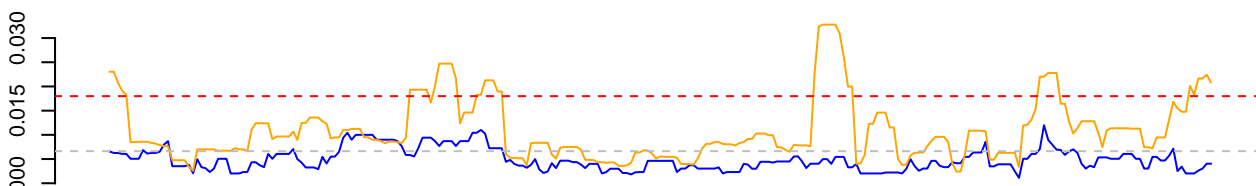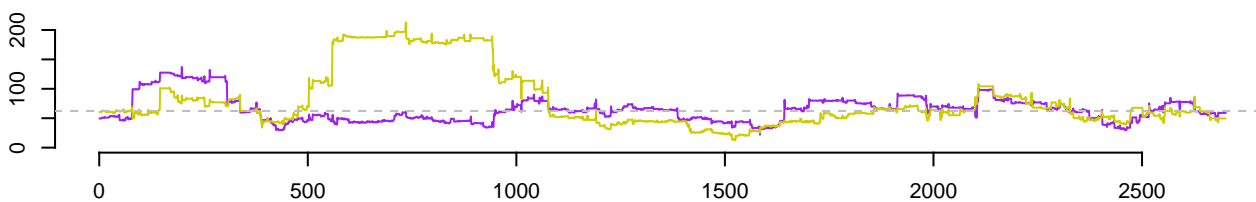

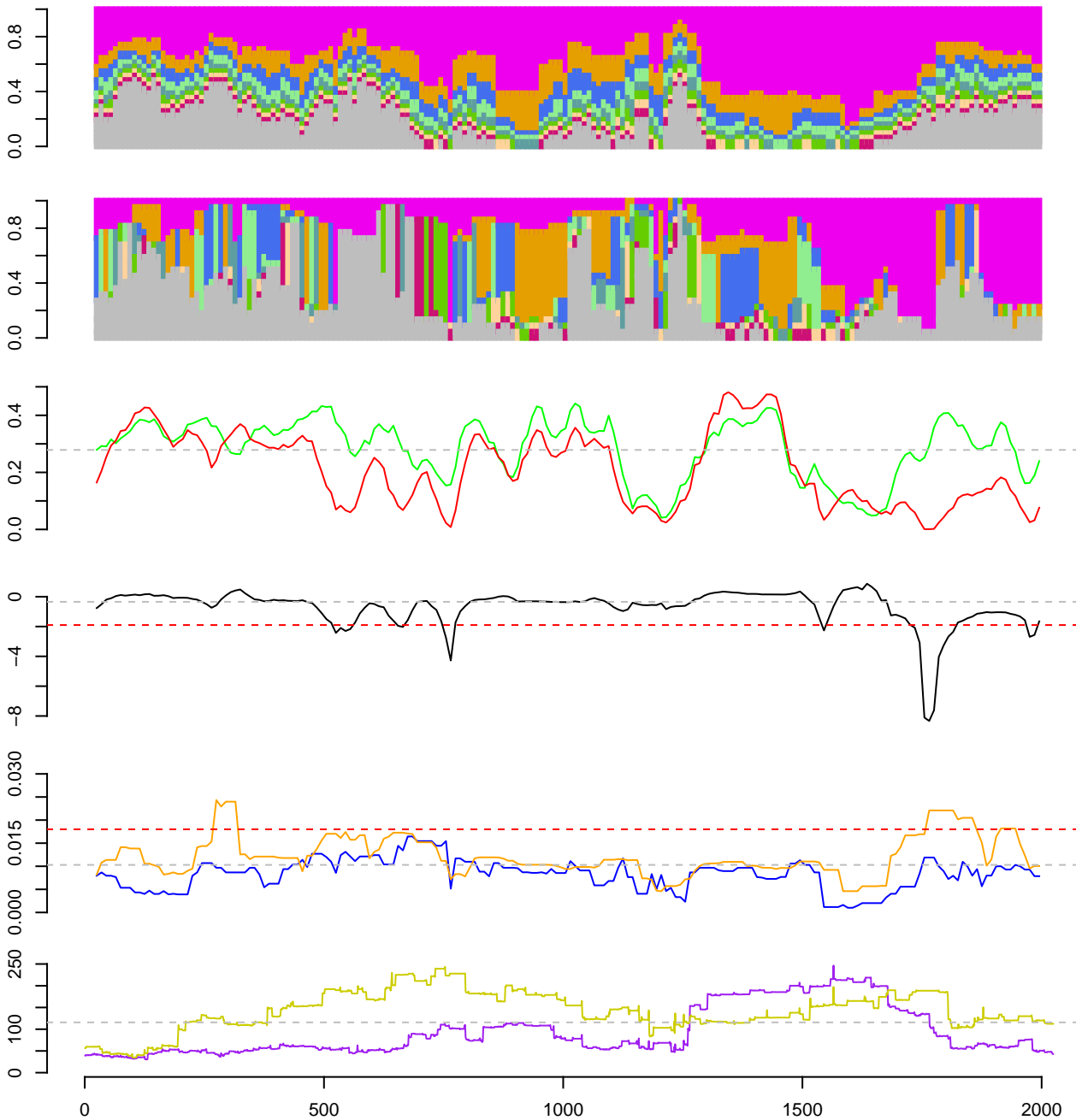

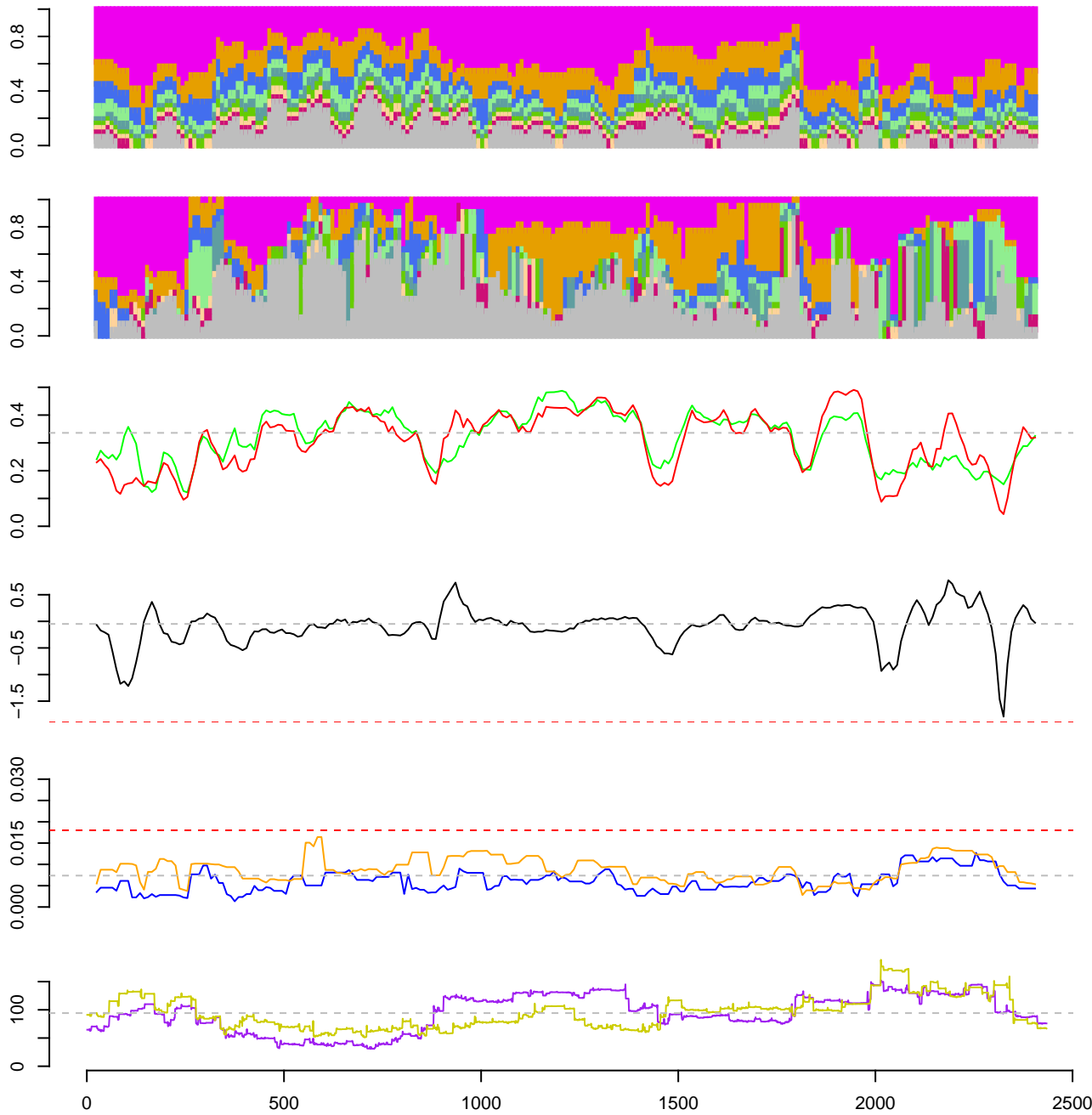

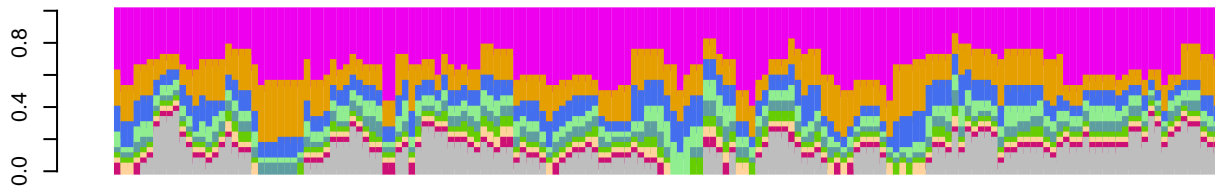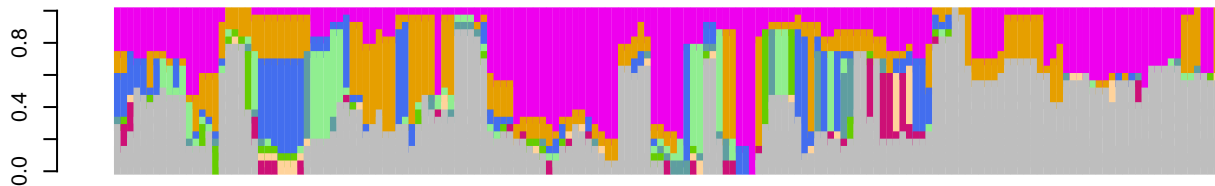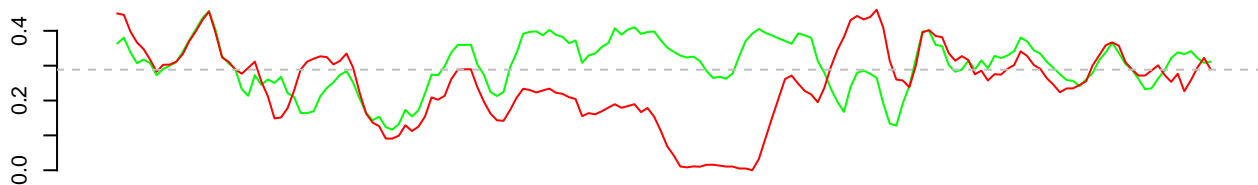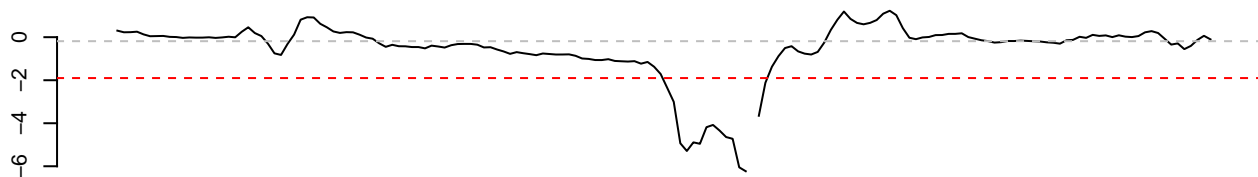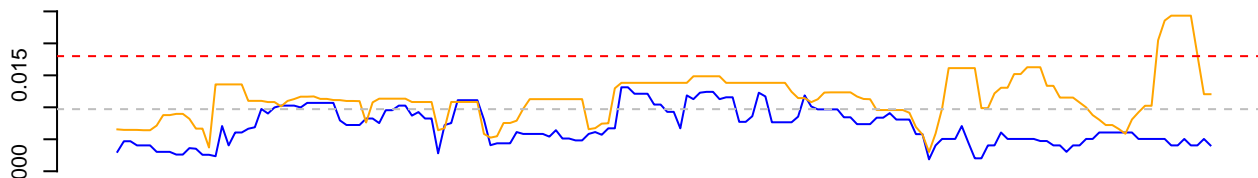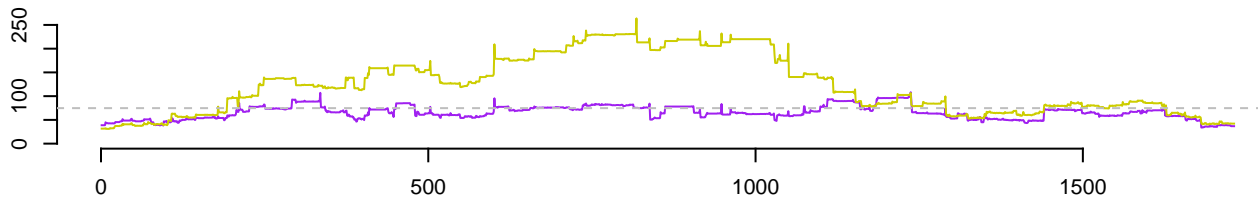

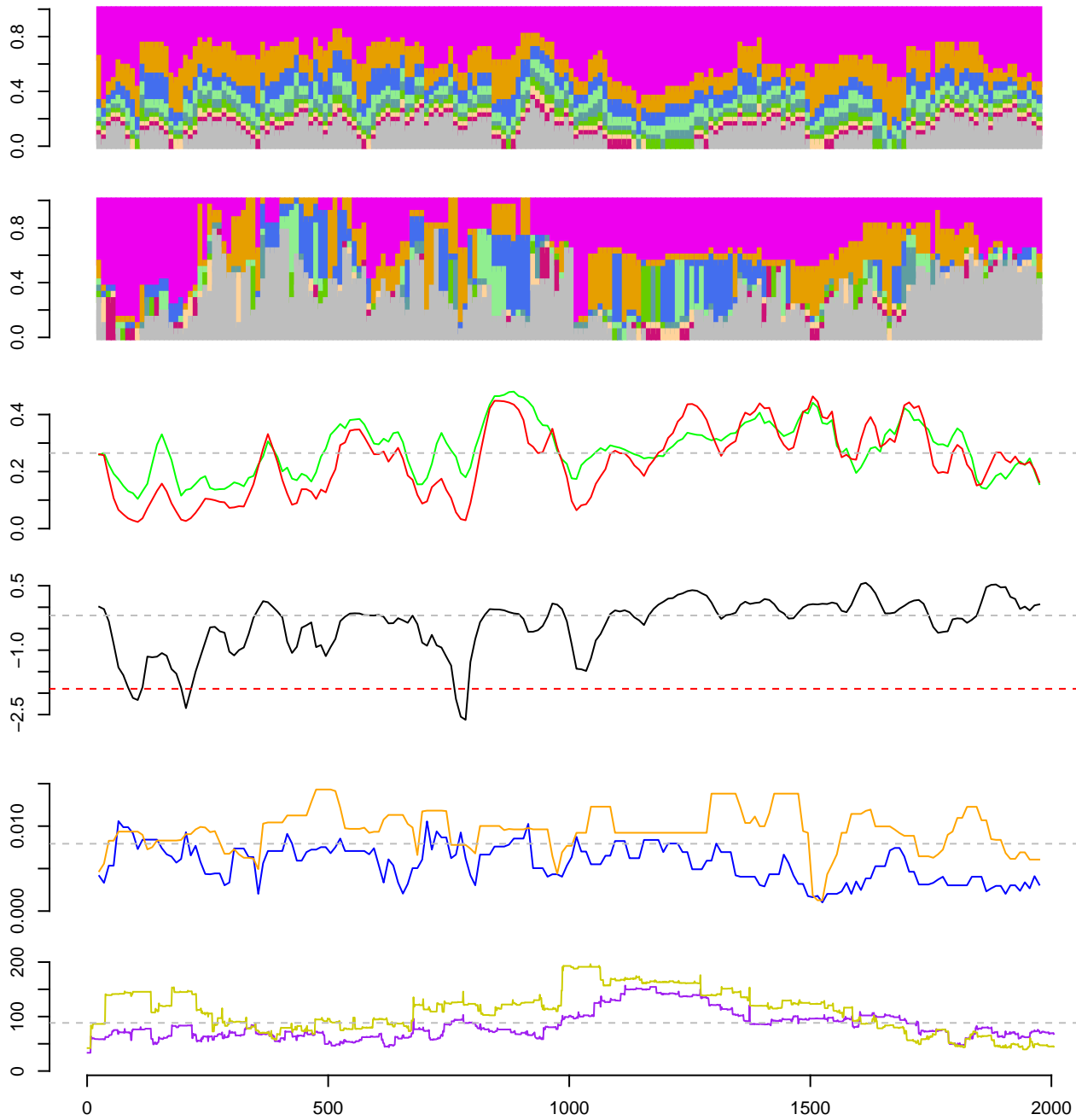

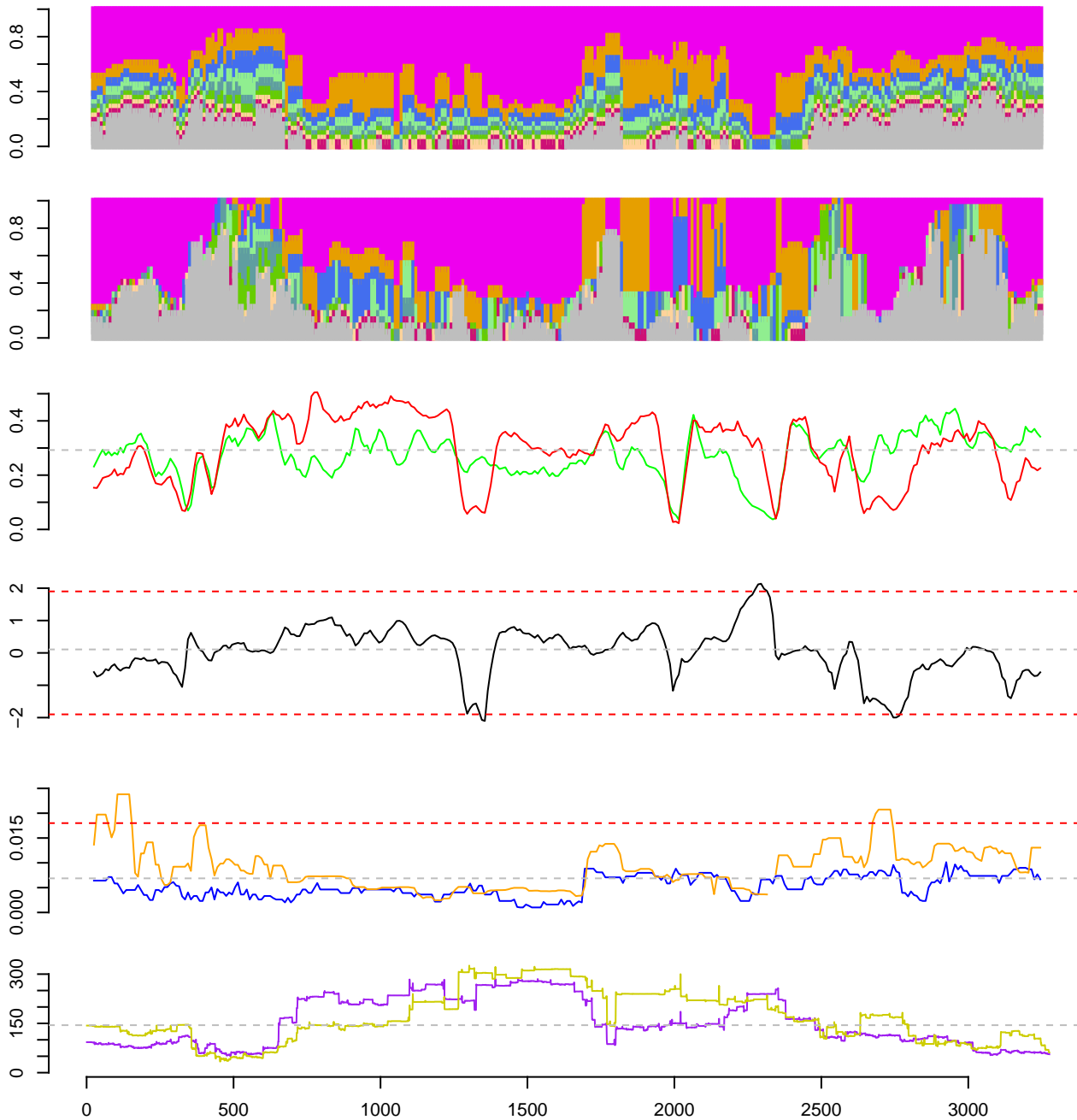

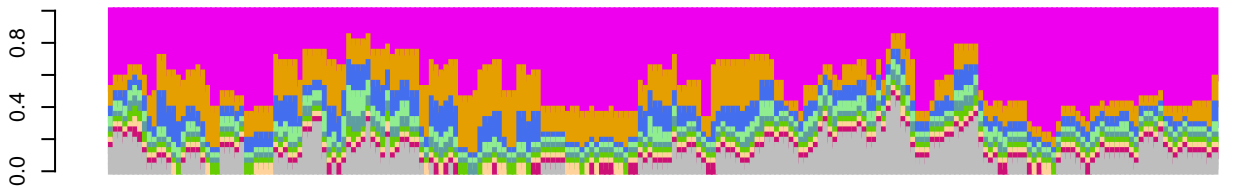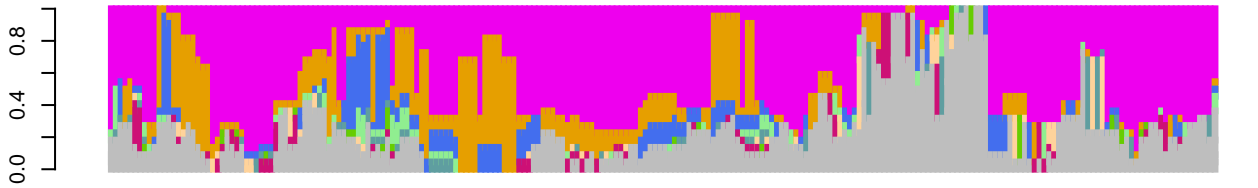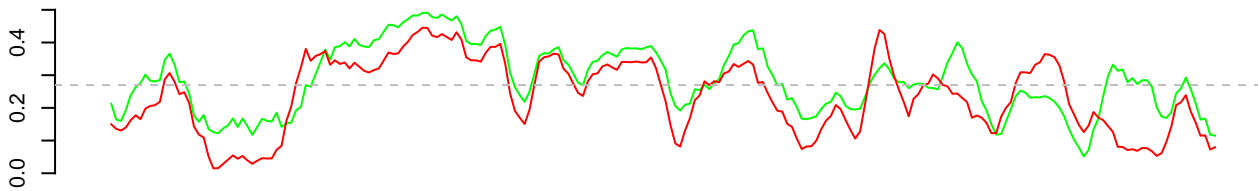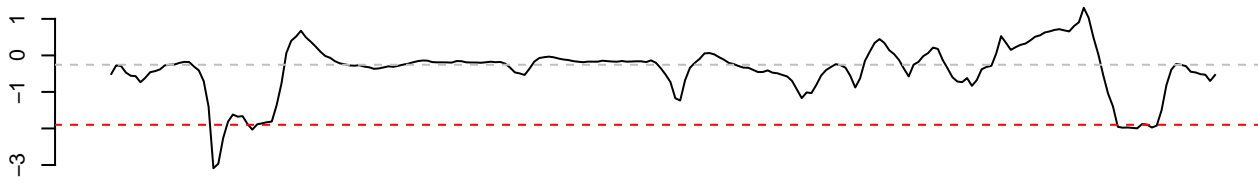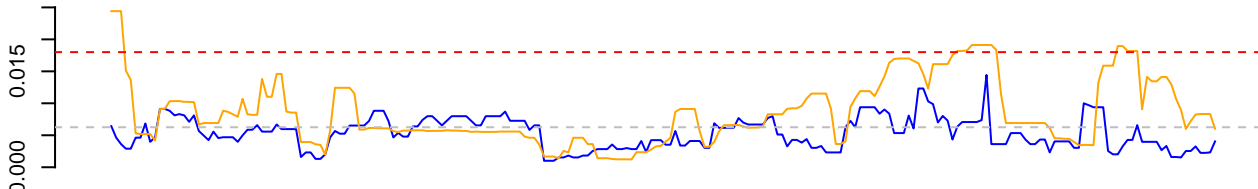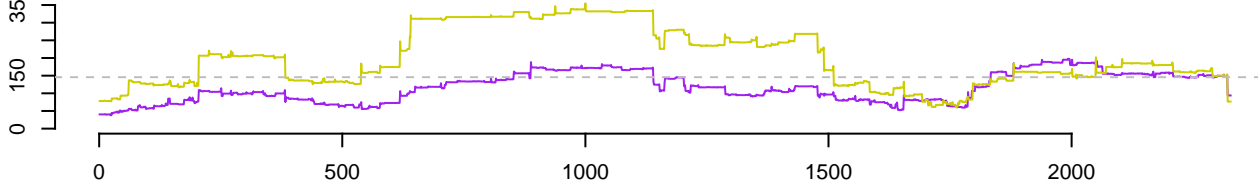

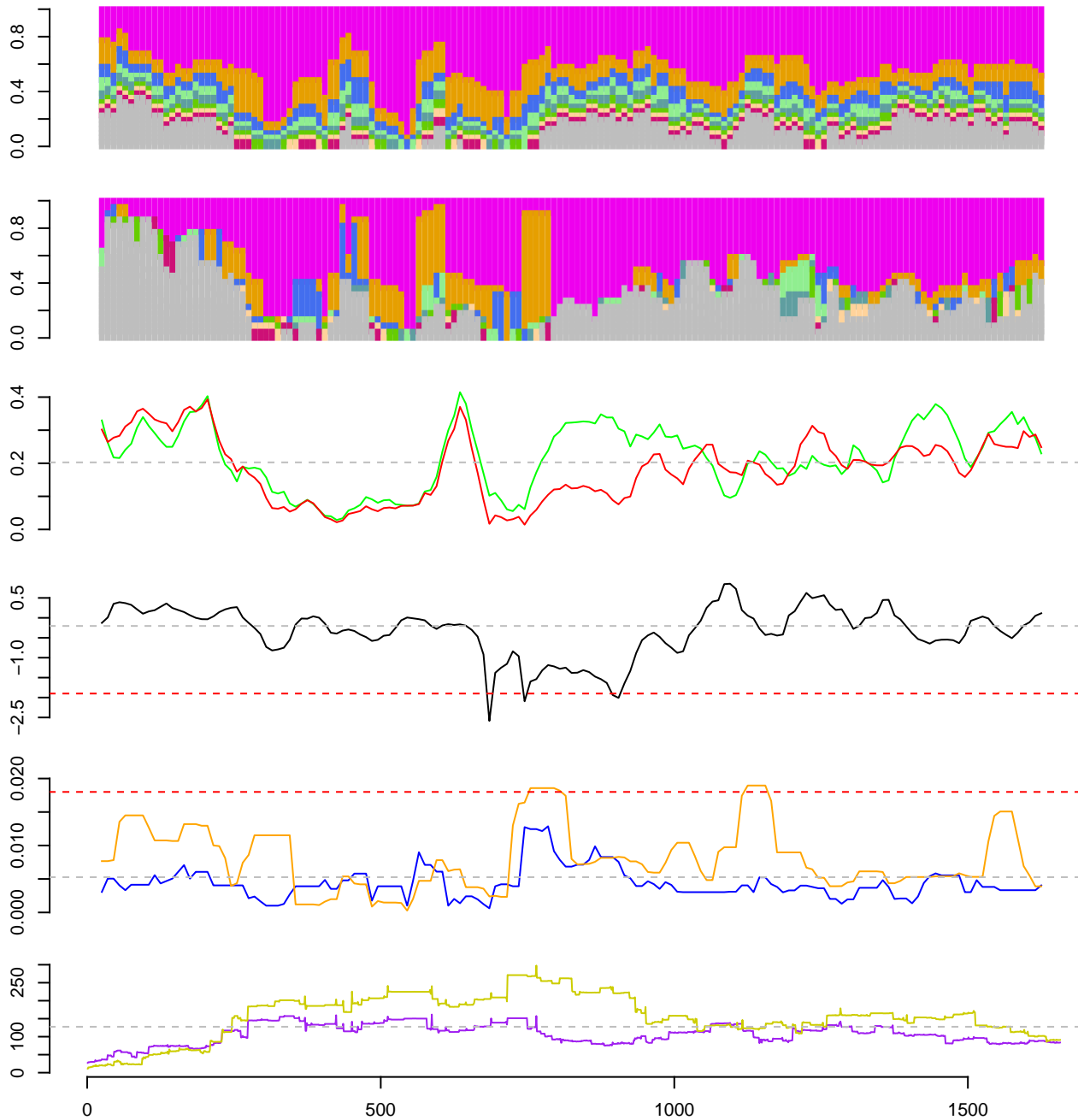

Supplement: Supplemental Material [file supp_g3.116.029215_FileS2.zip › MG3_4.pdf]

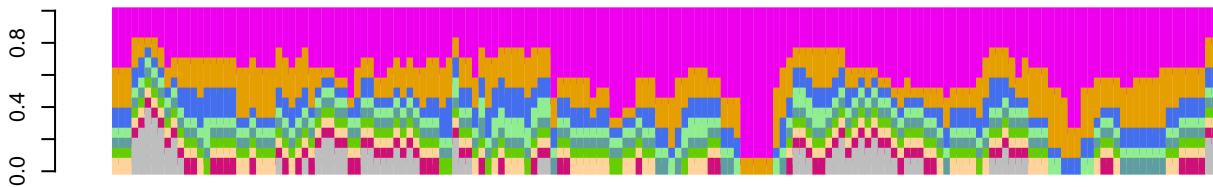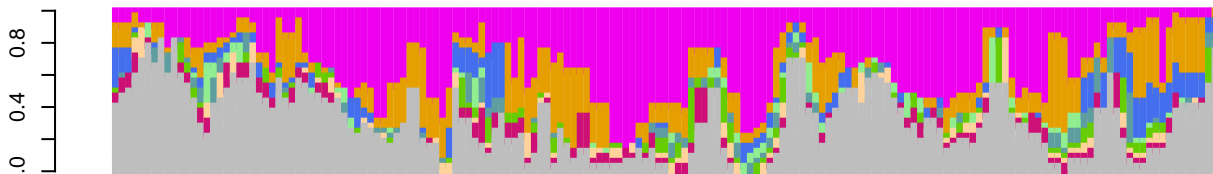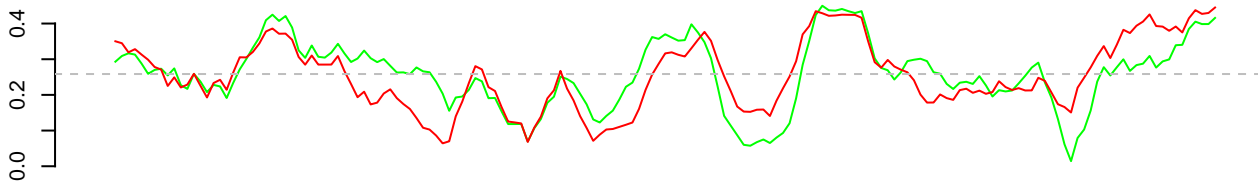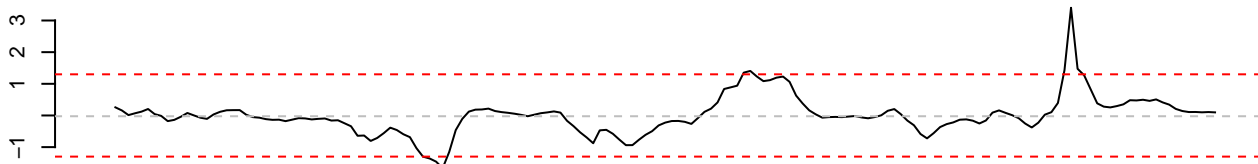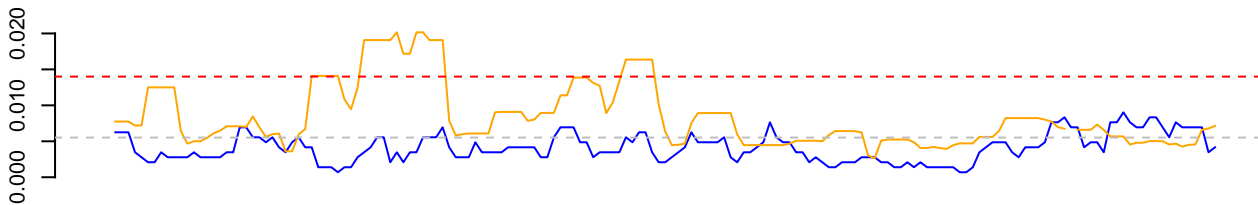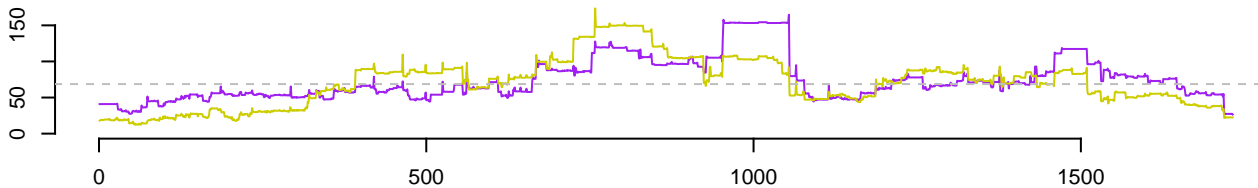

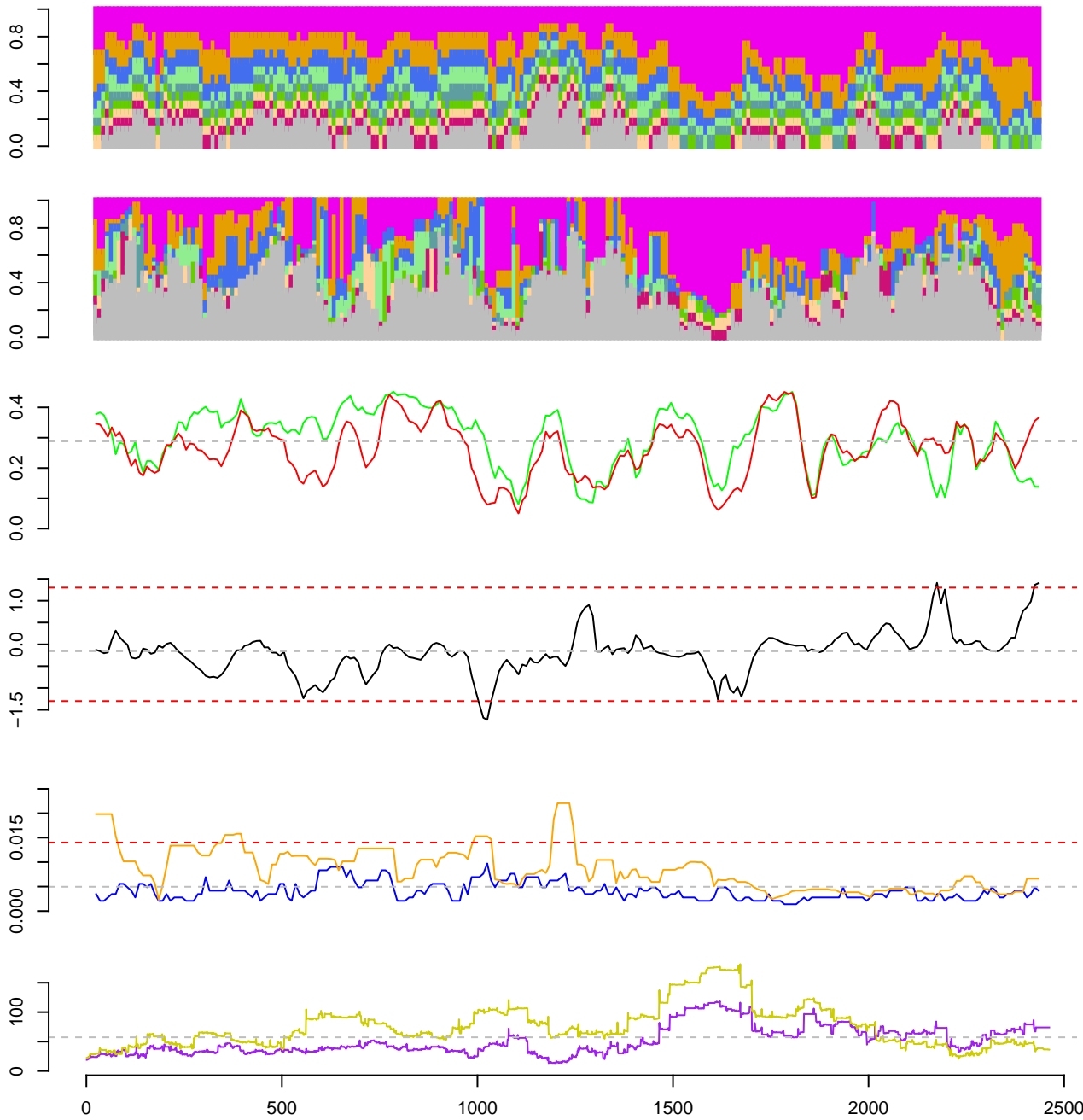

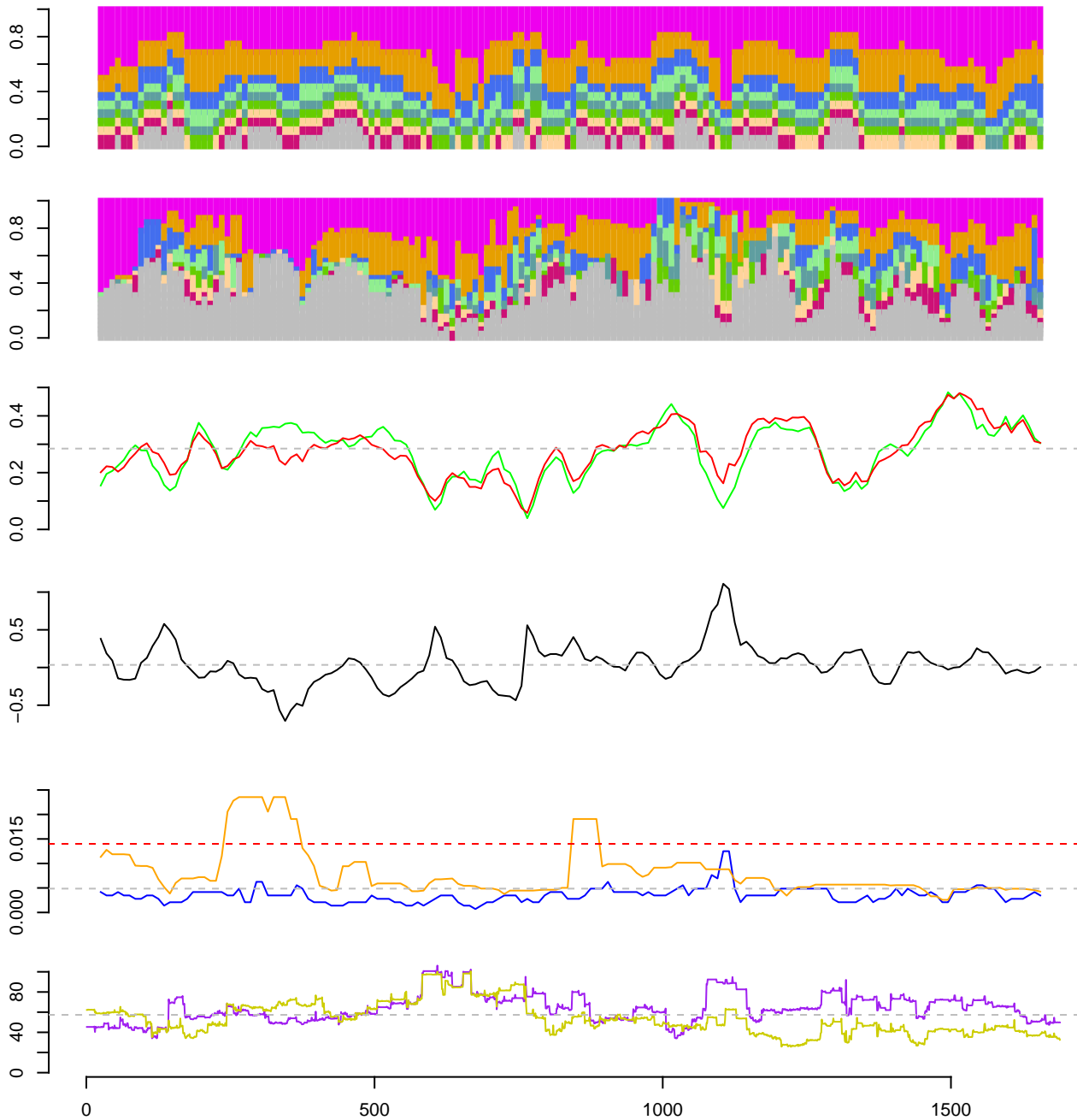

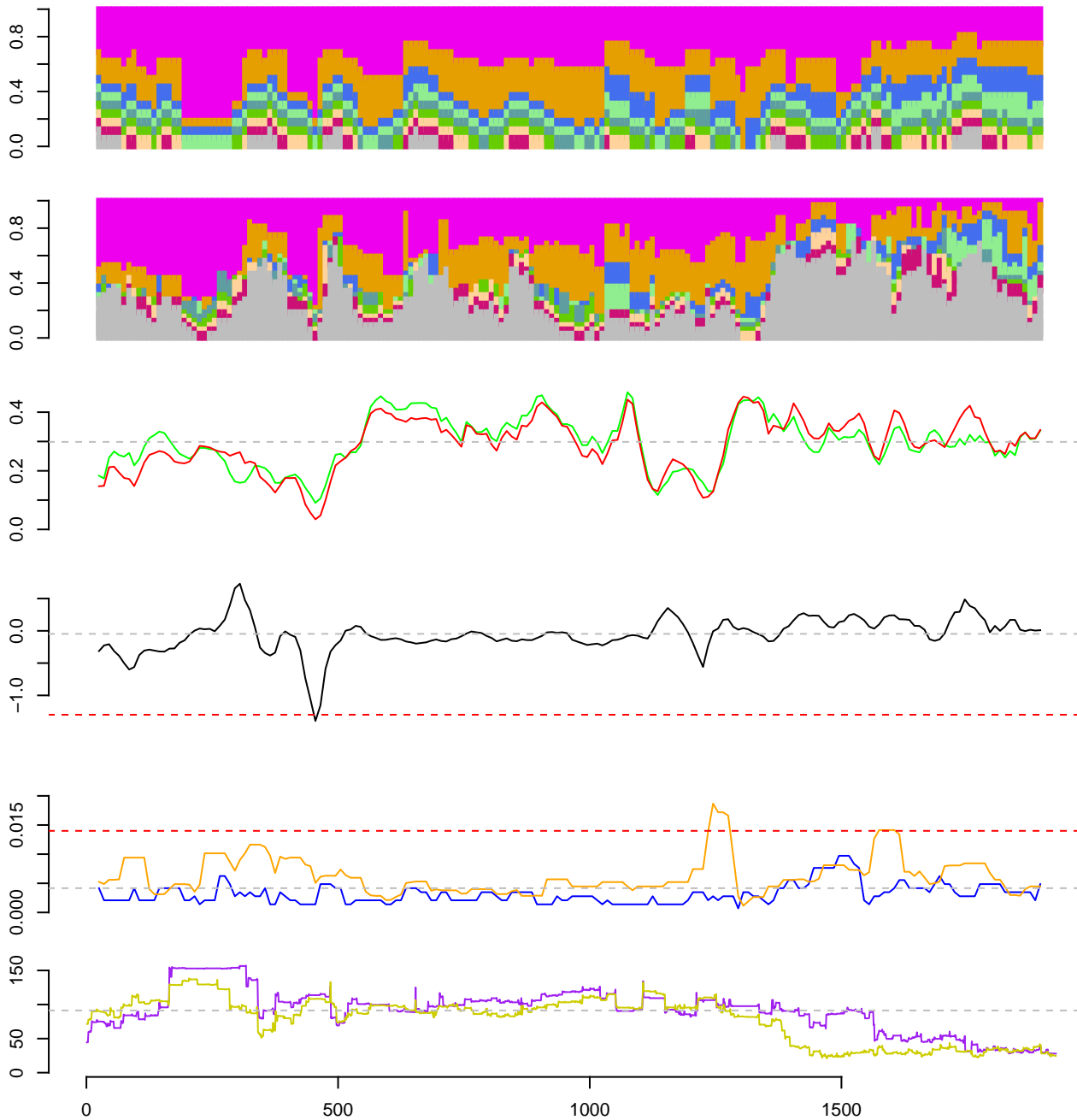

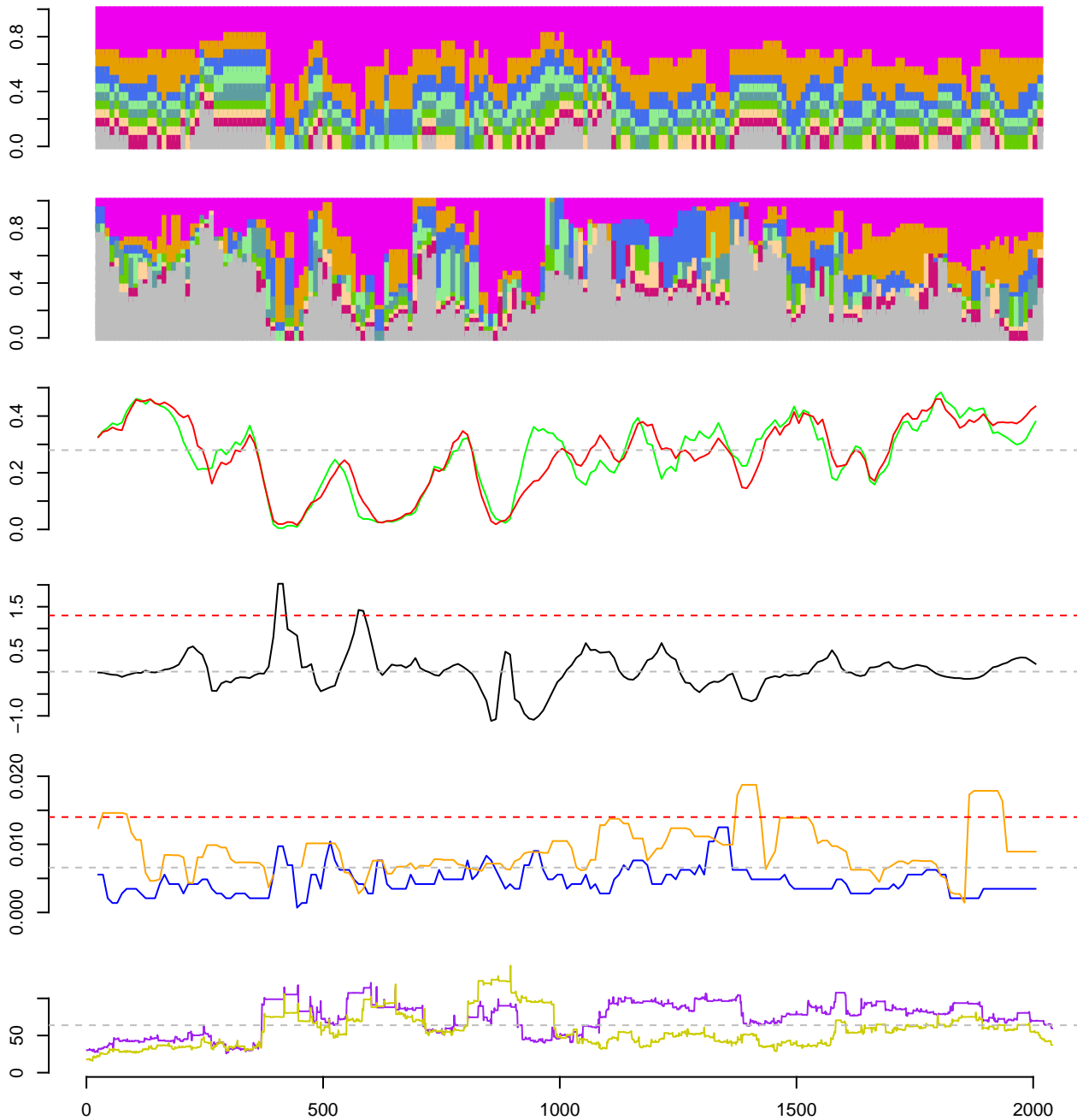

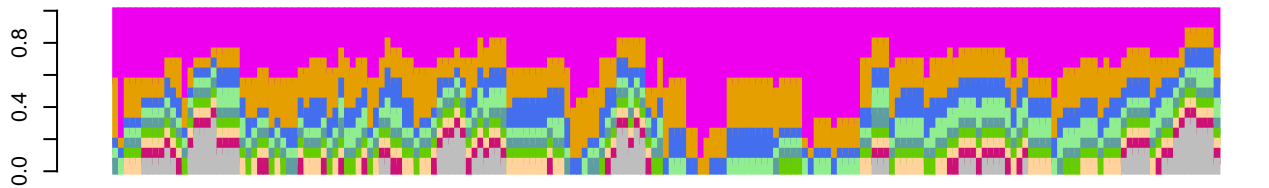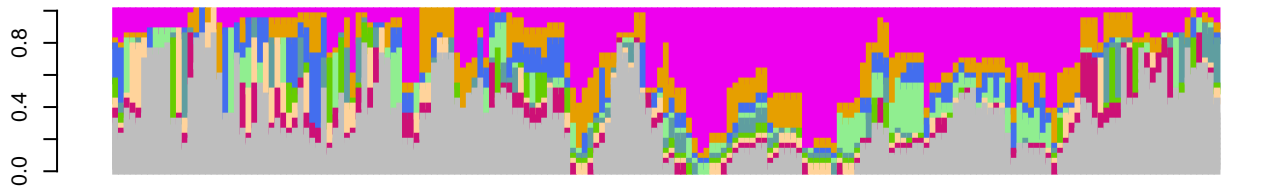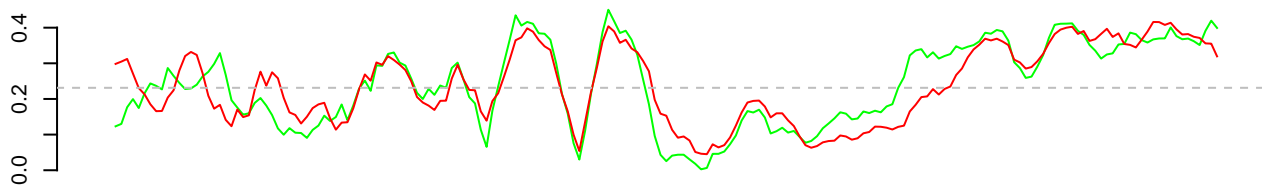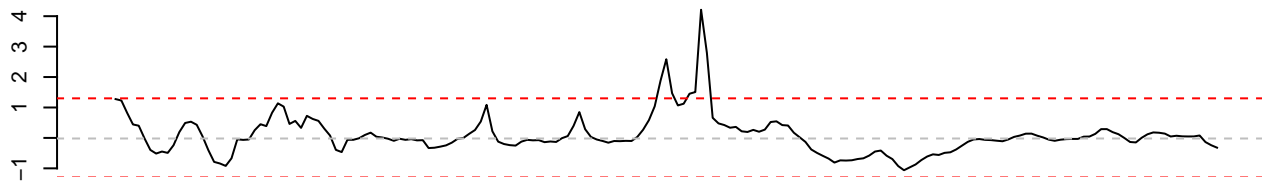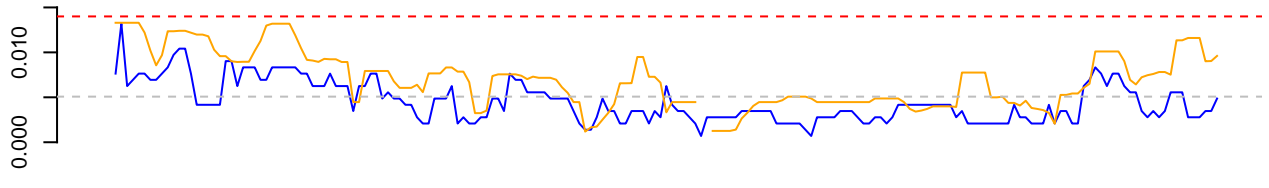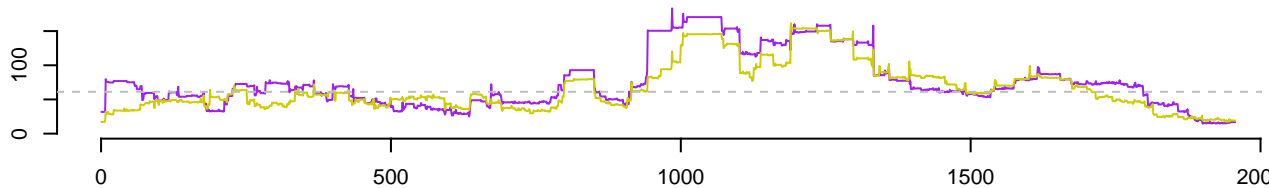

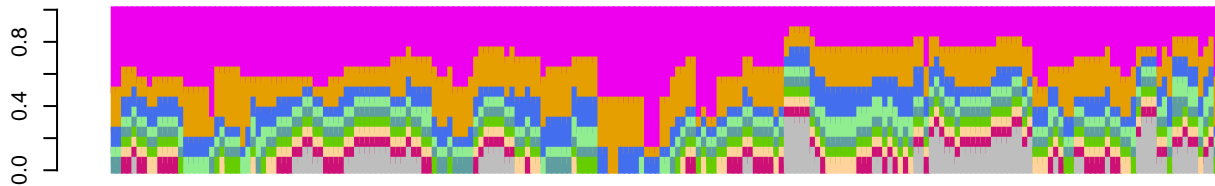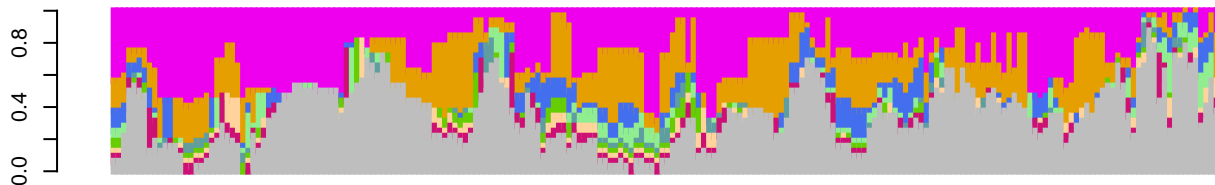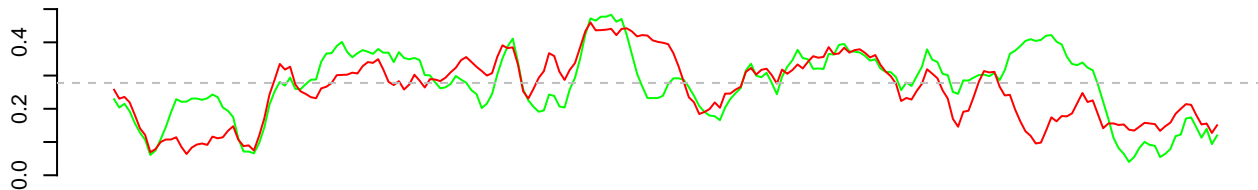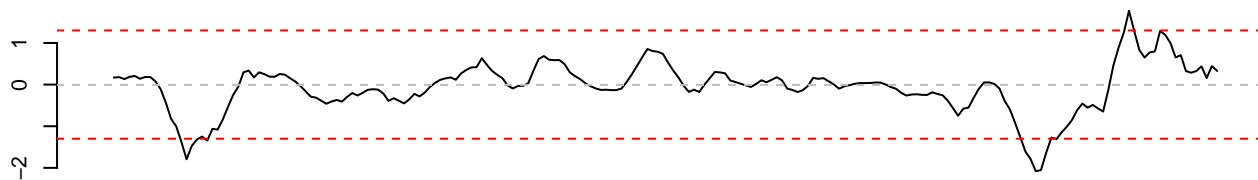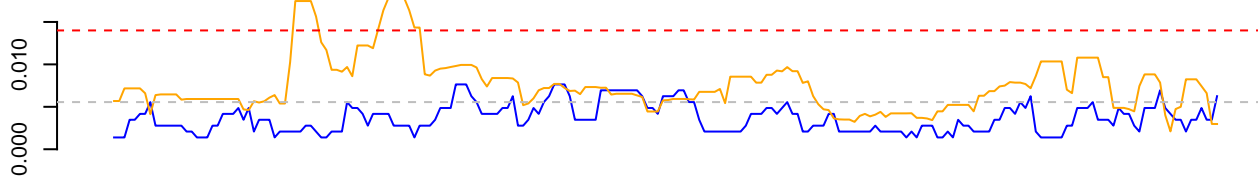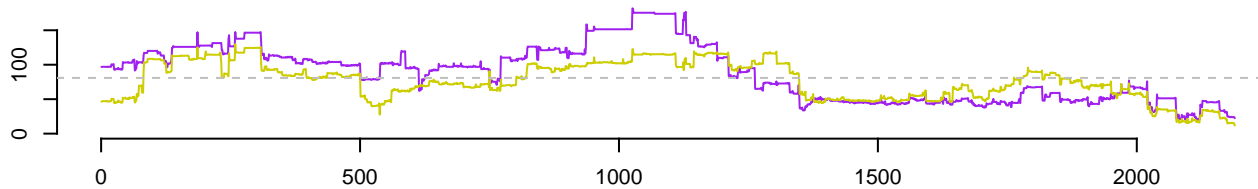

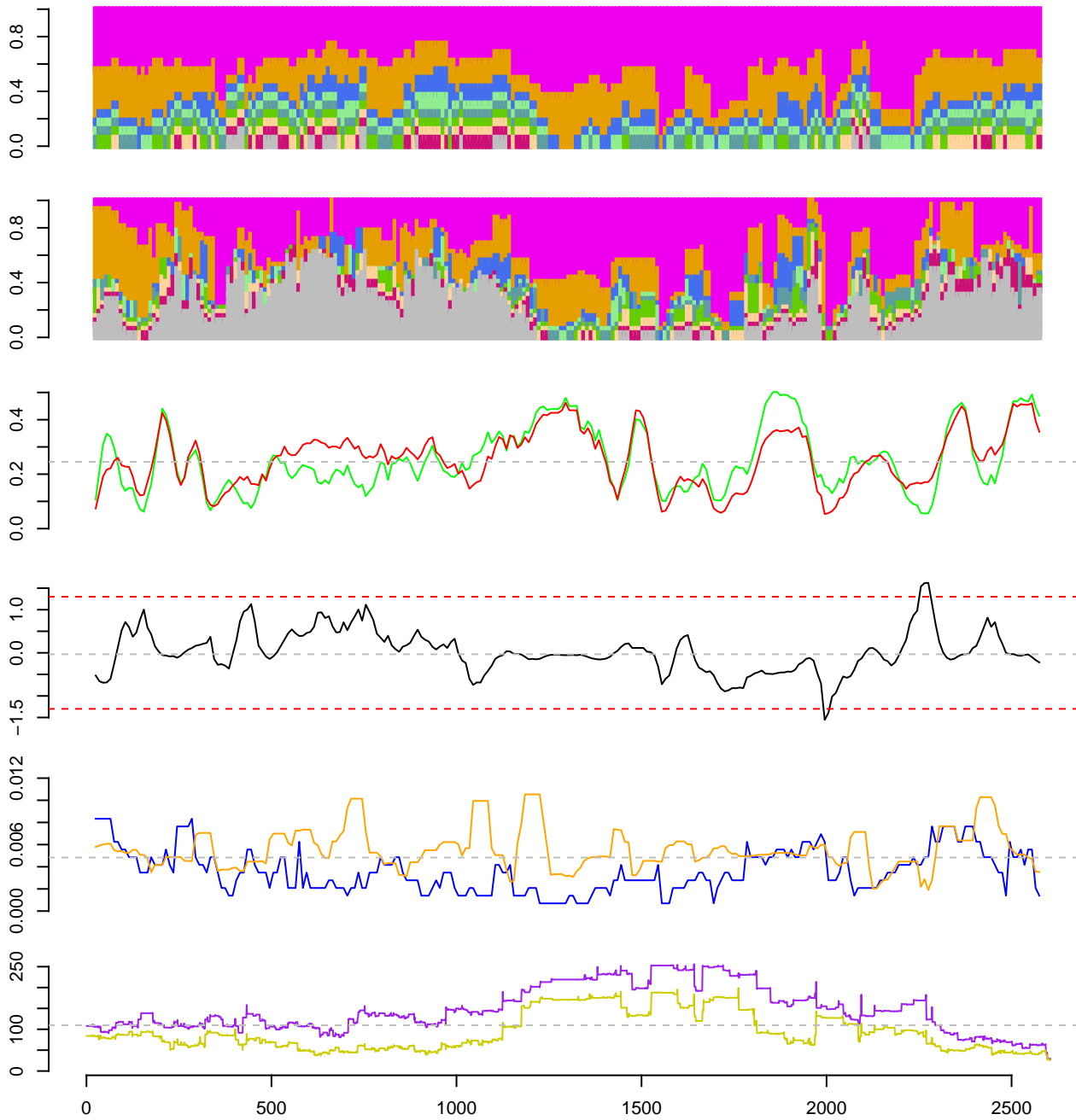

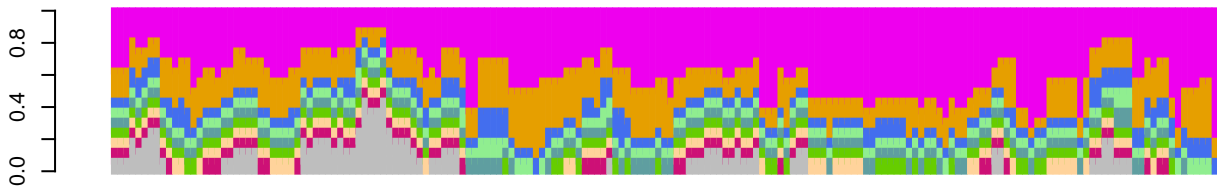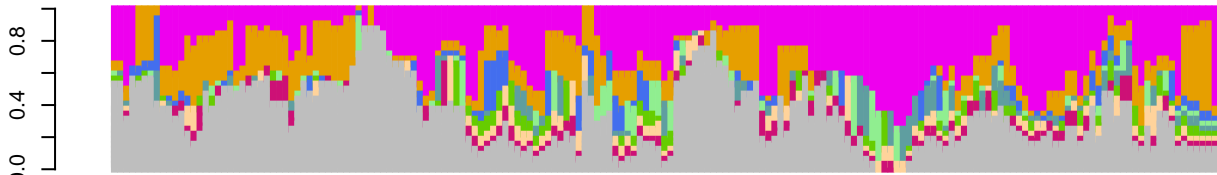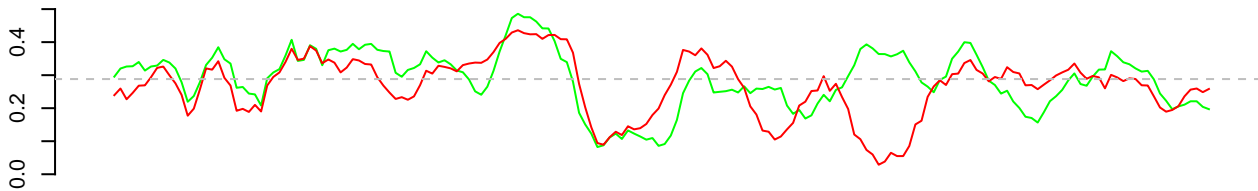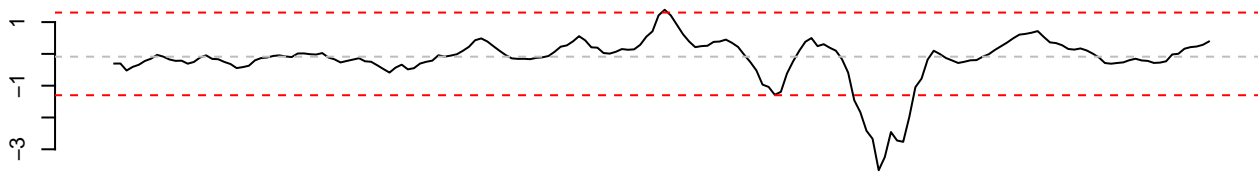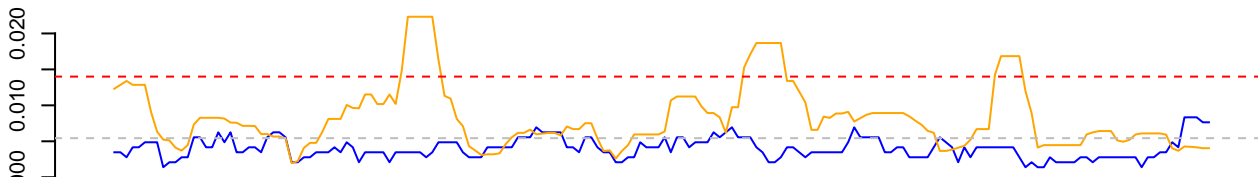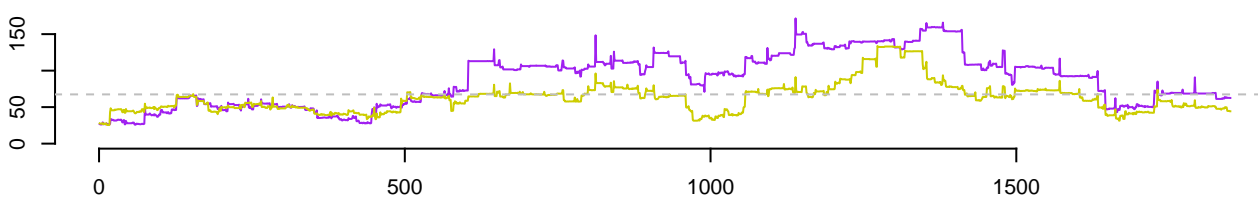

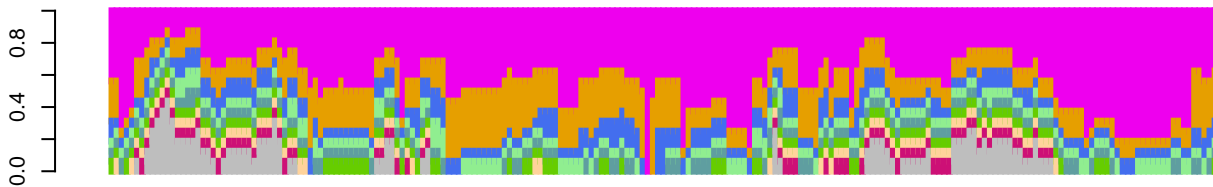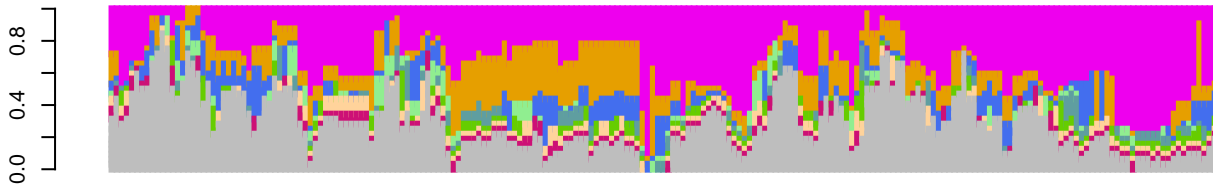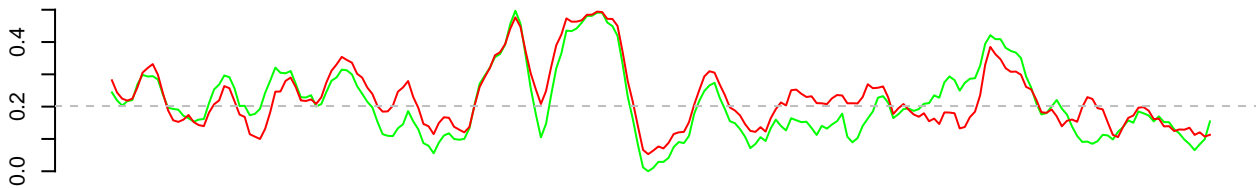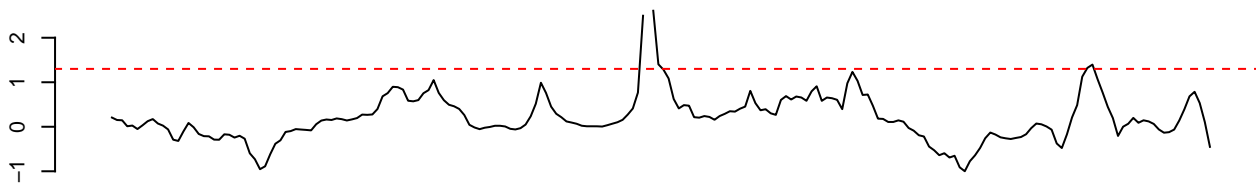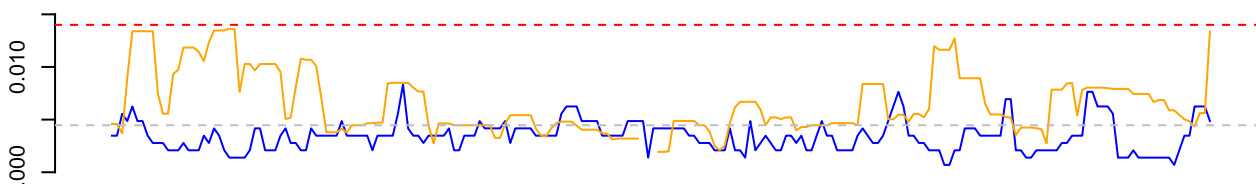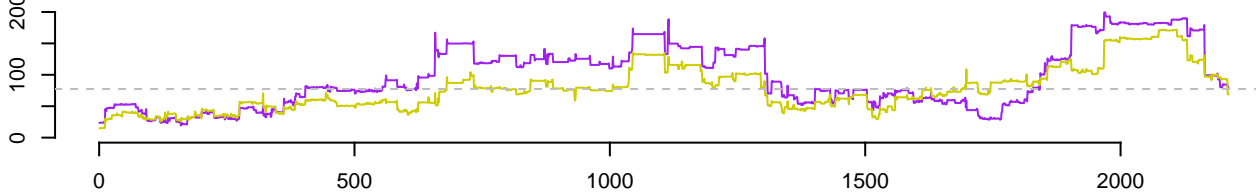

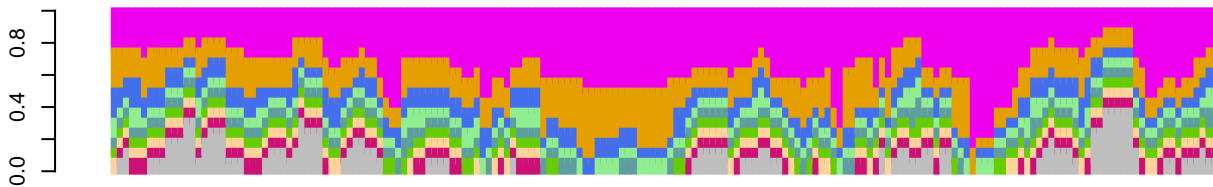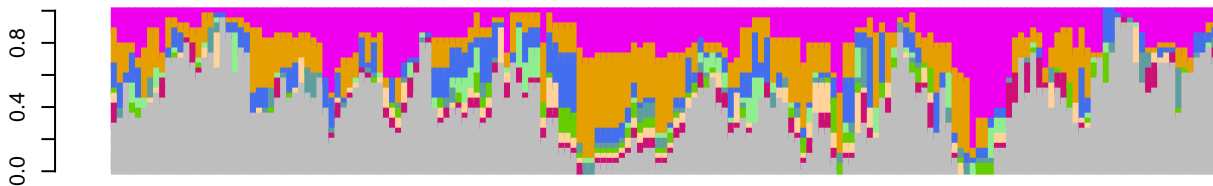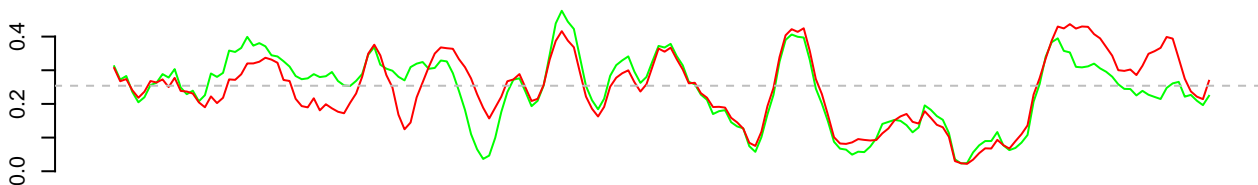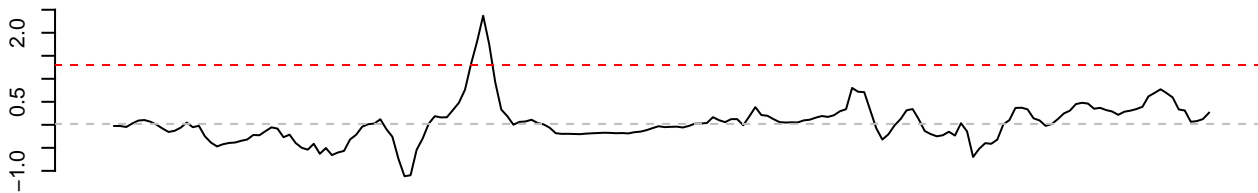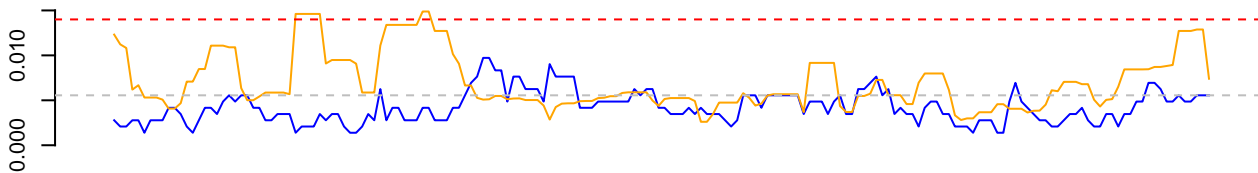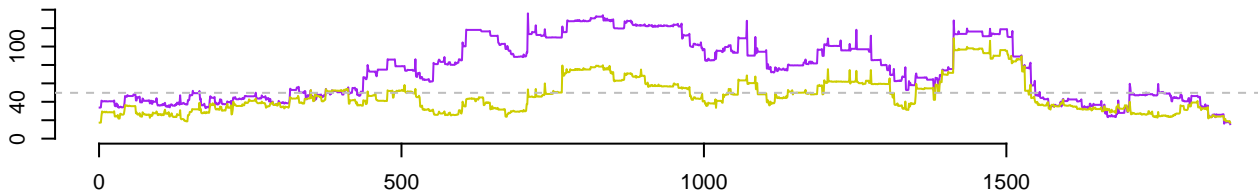

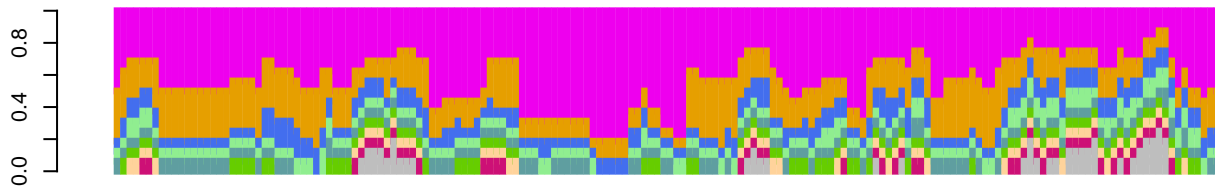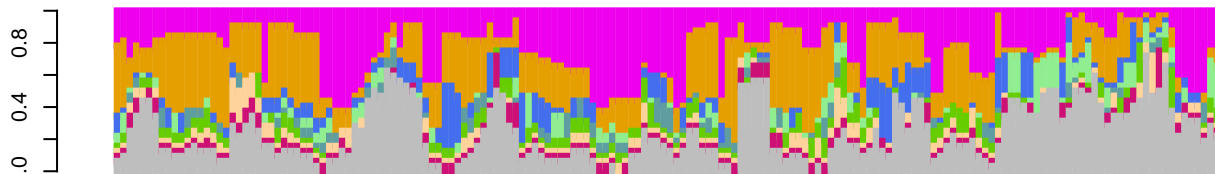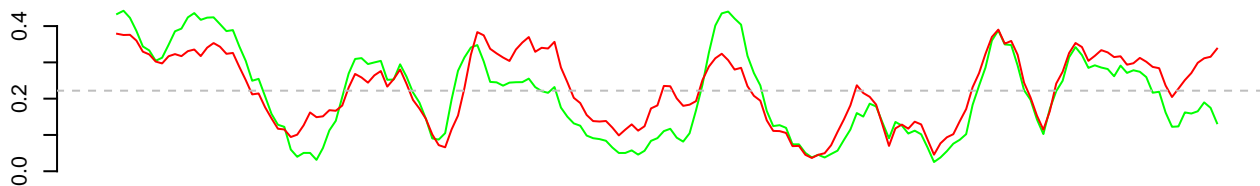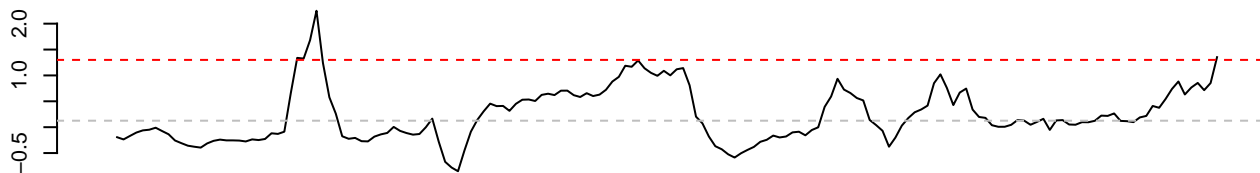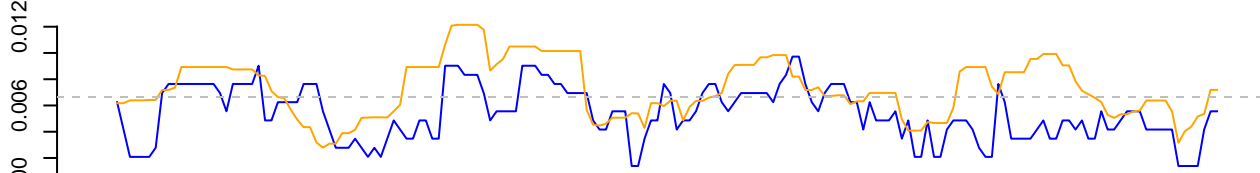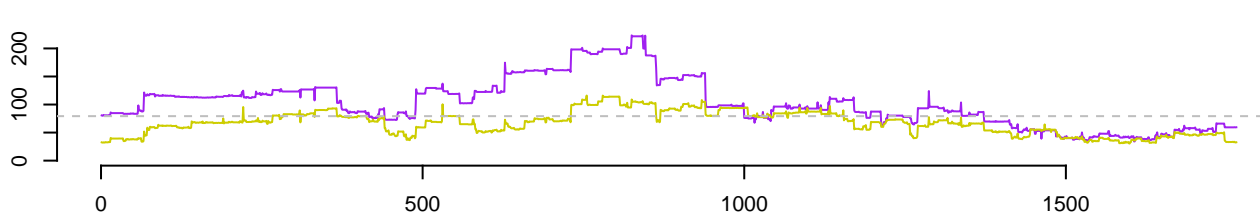

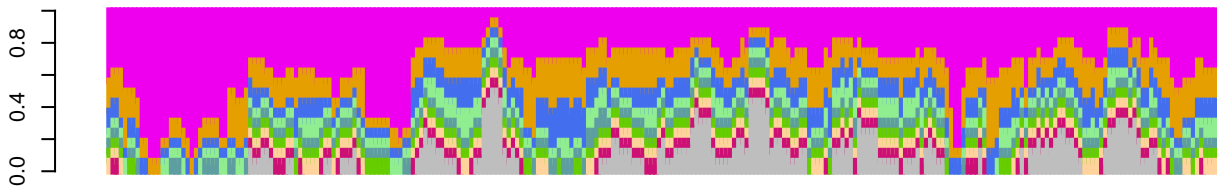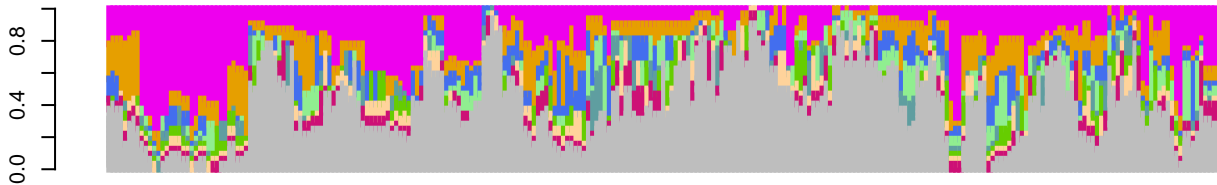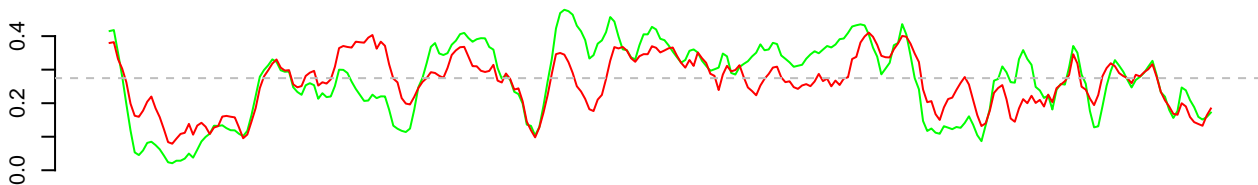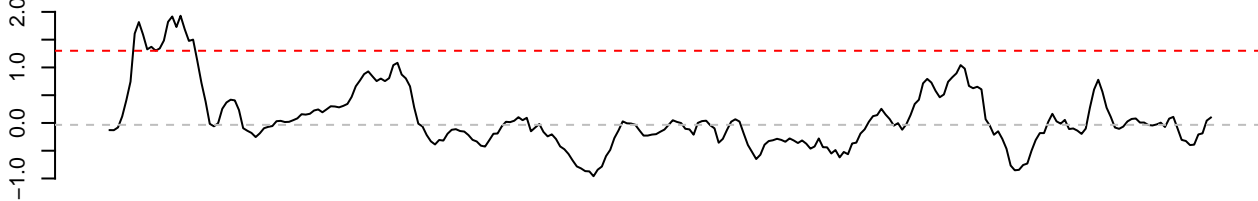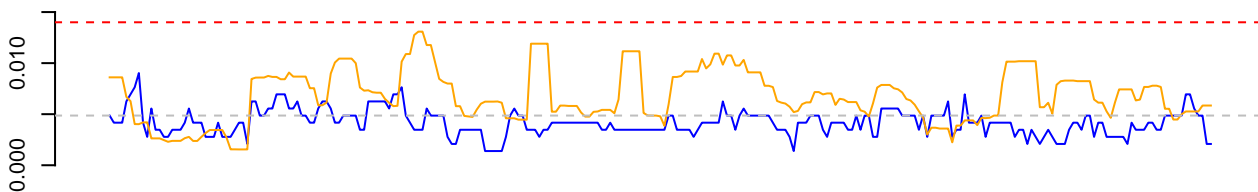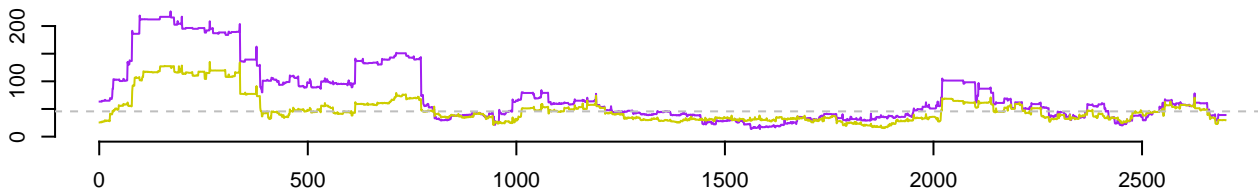

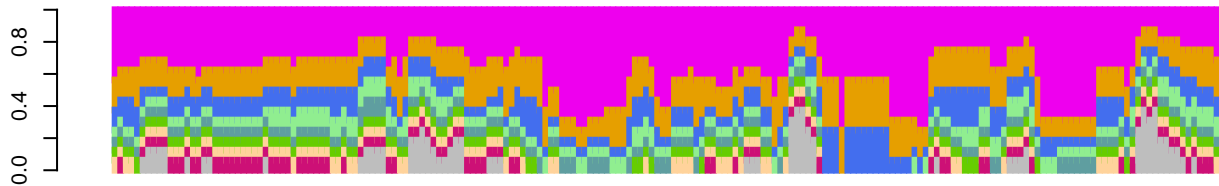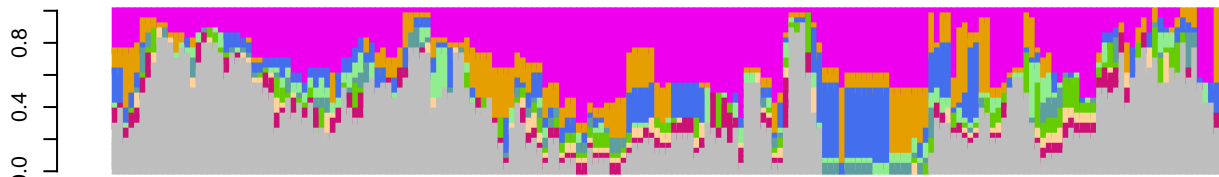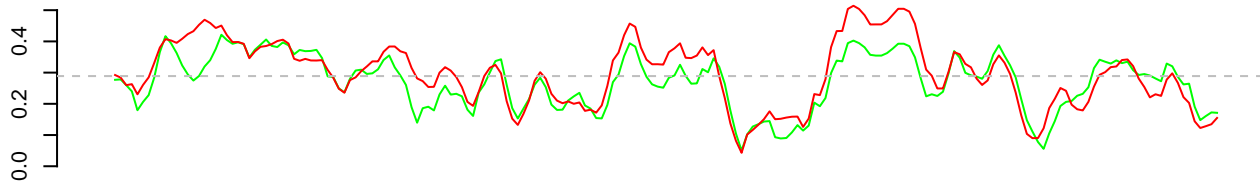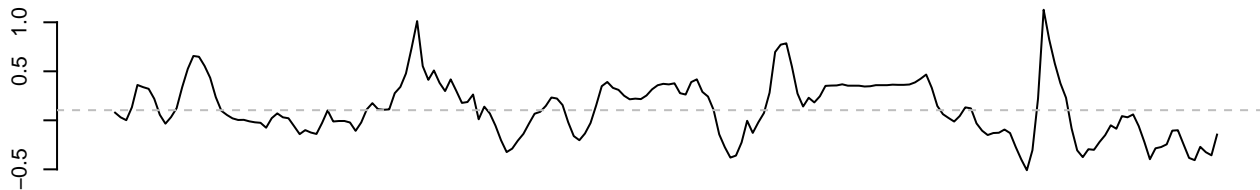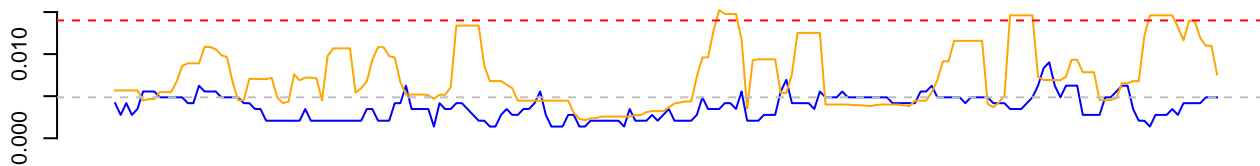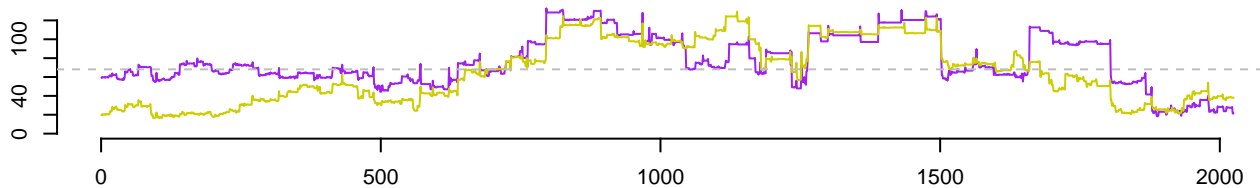

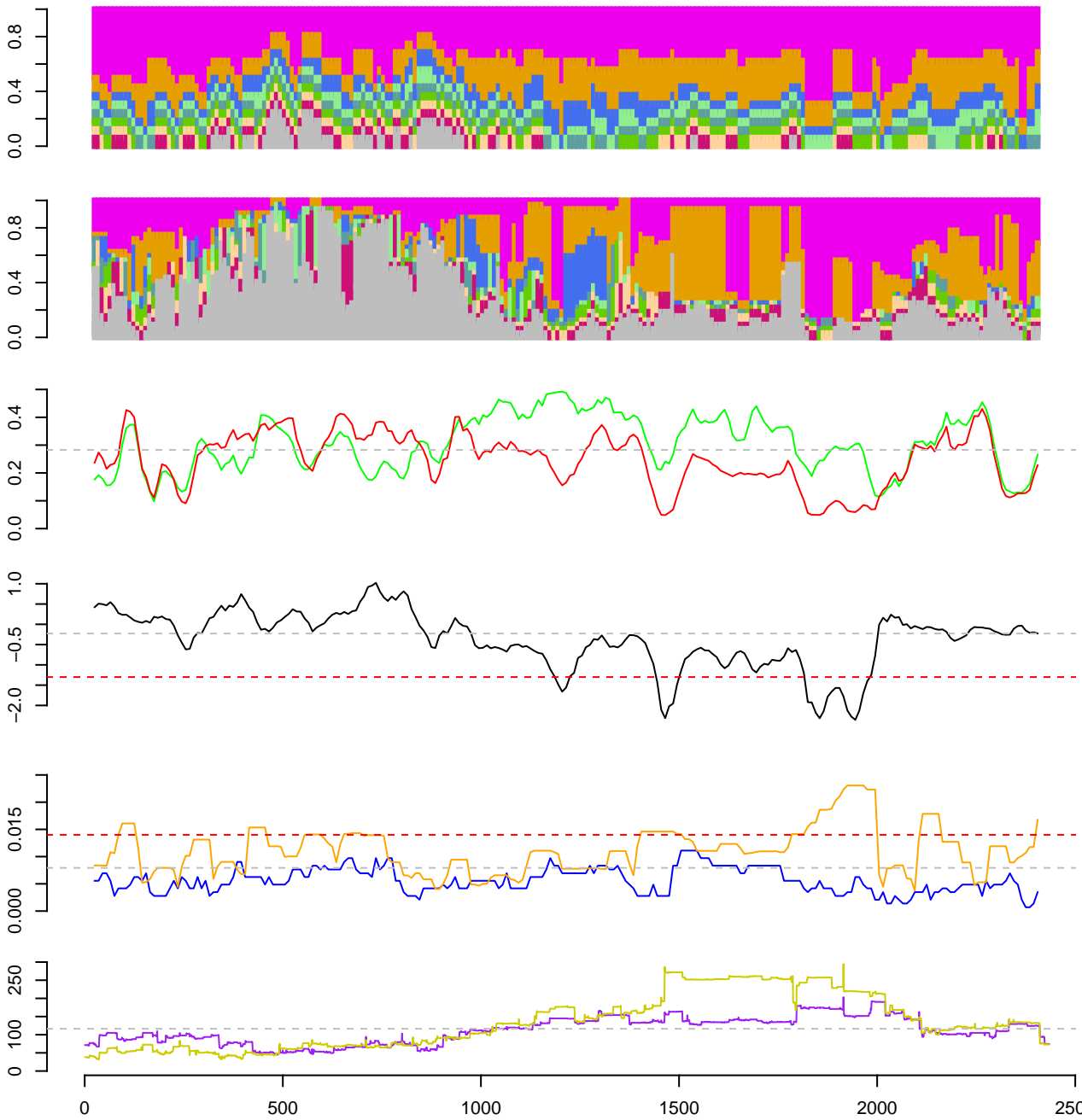

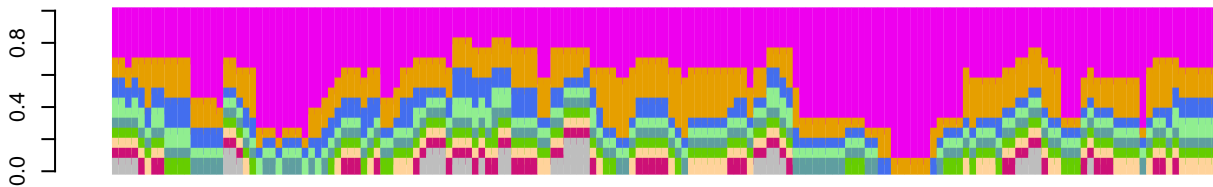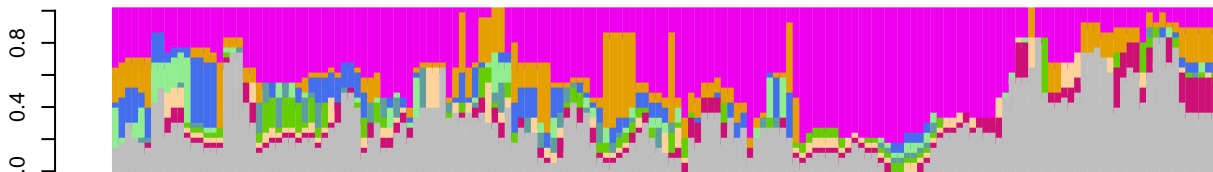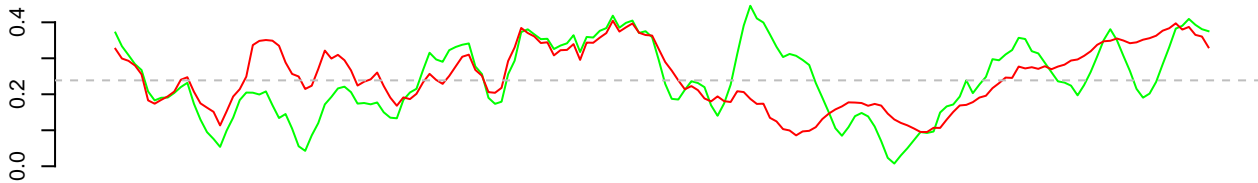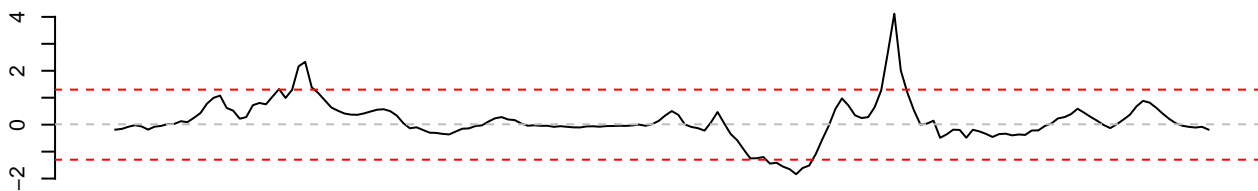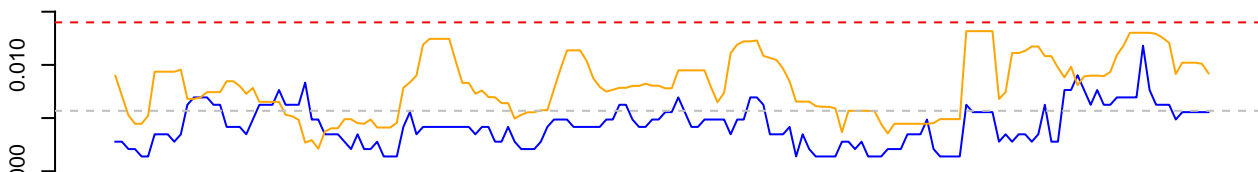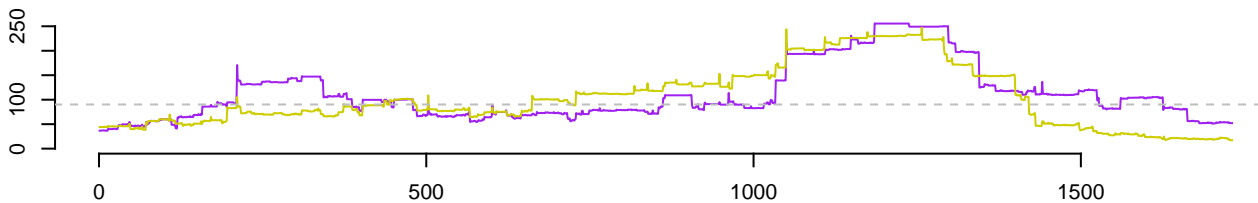

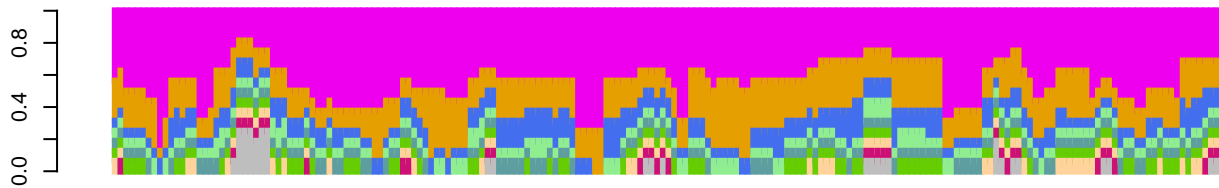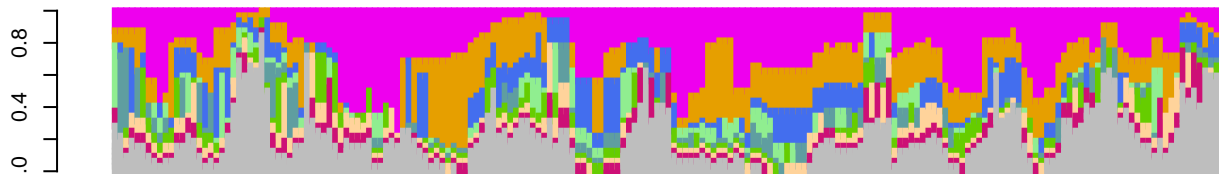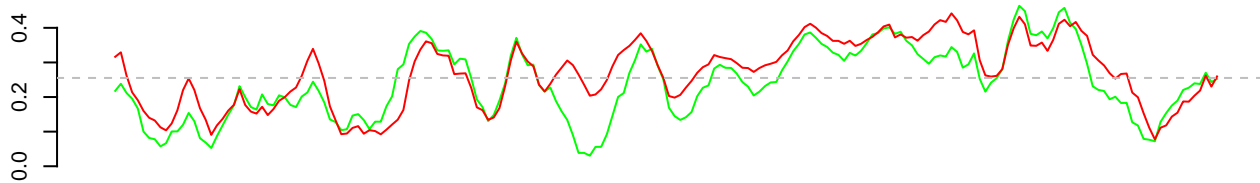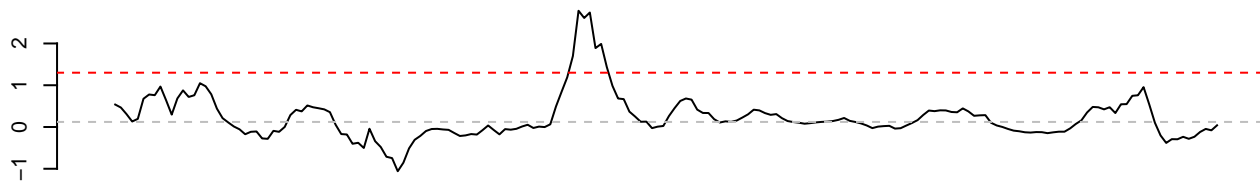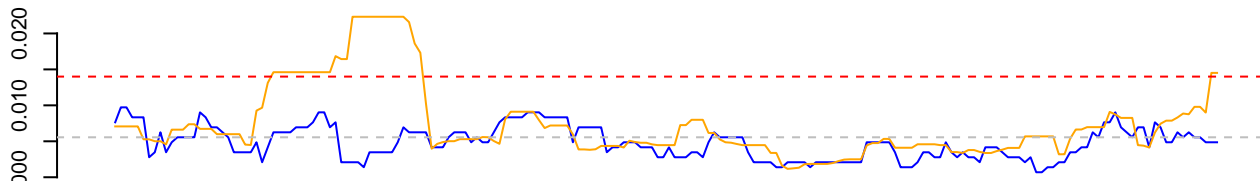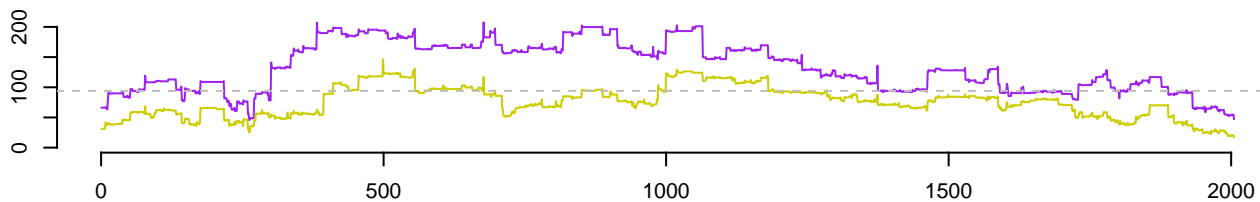

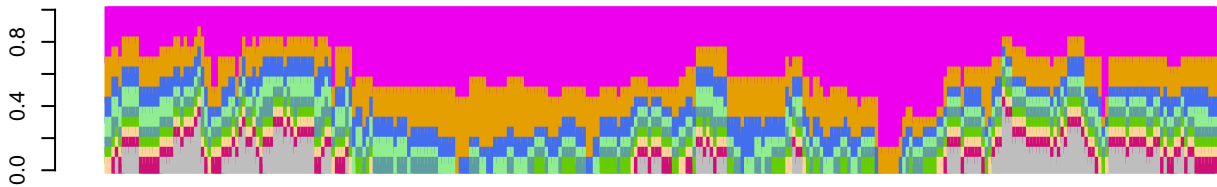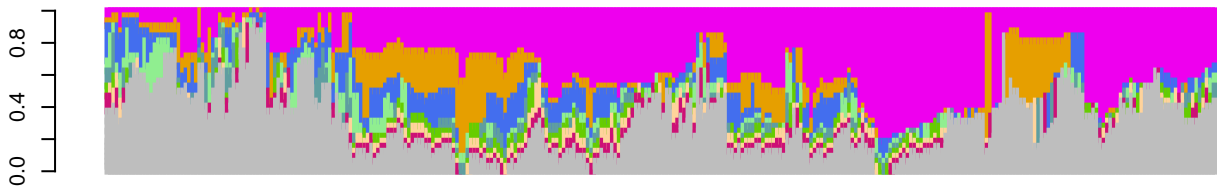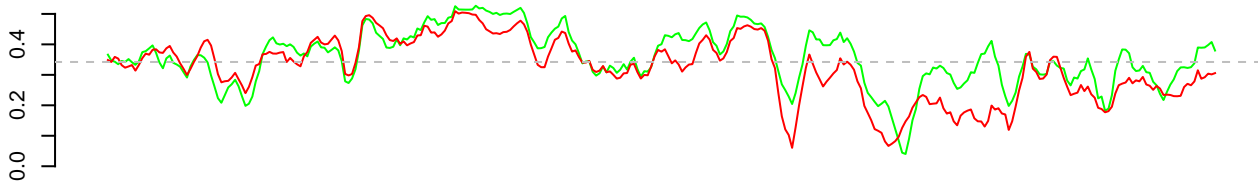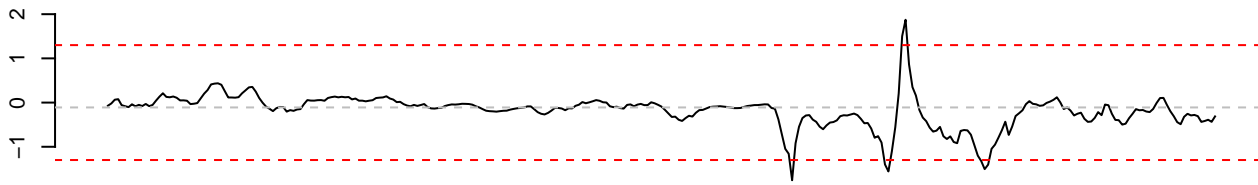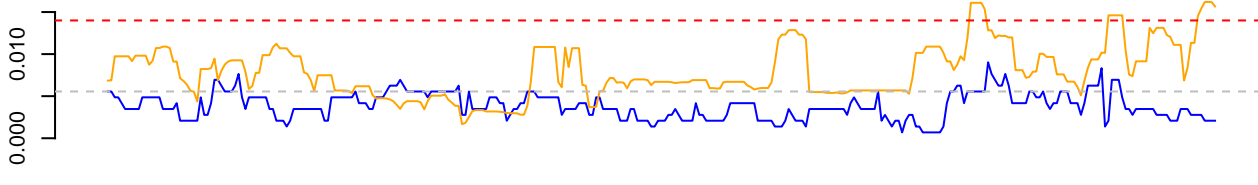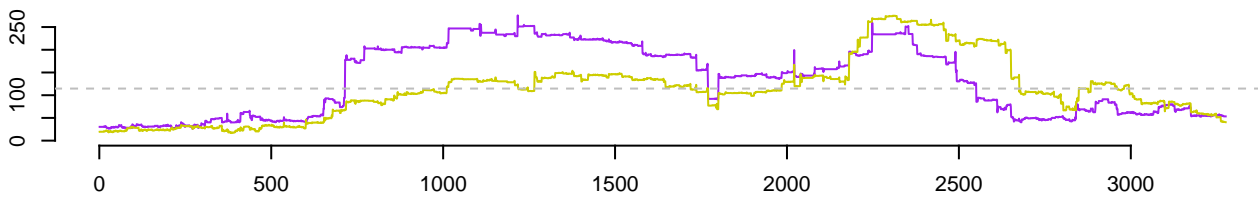

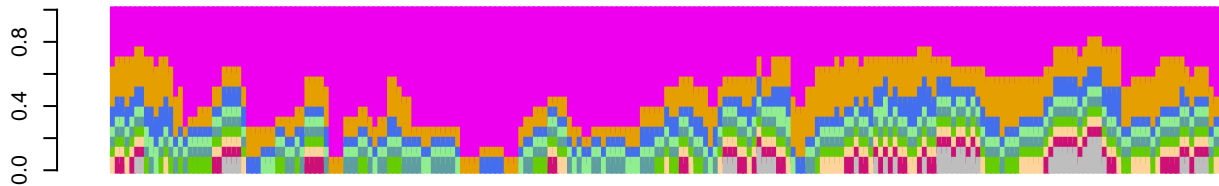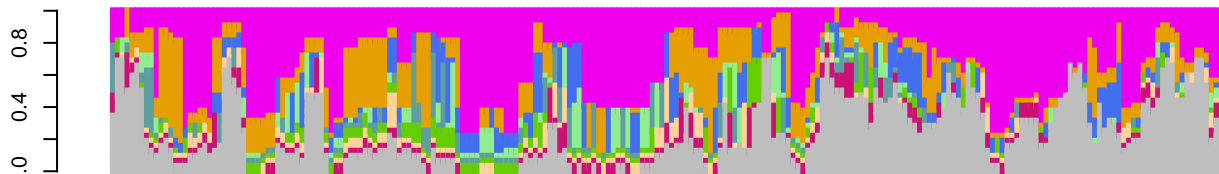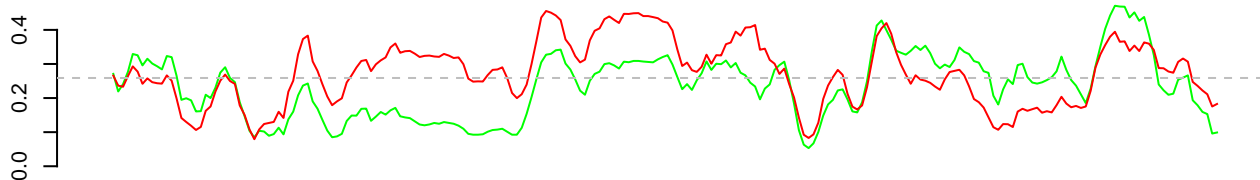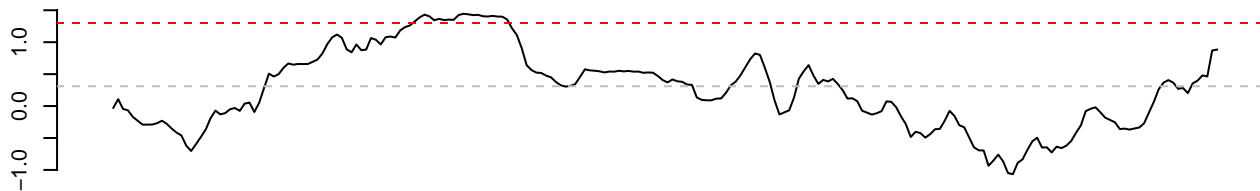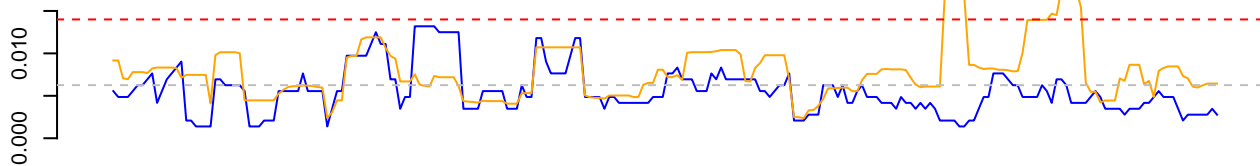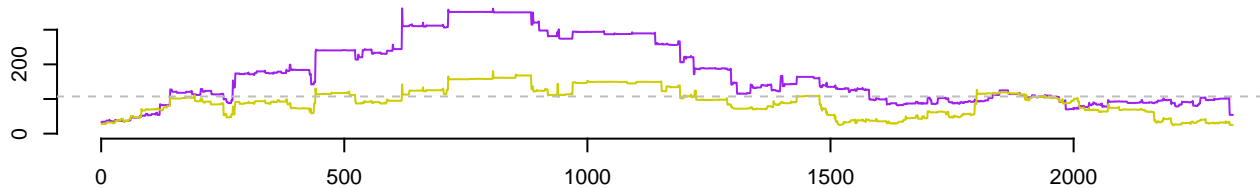

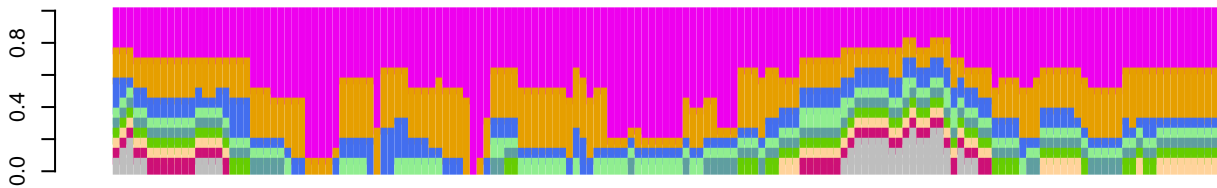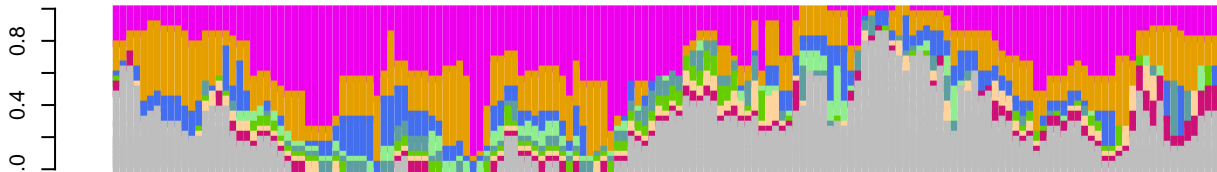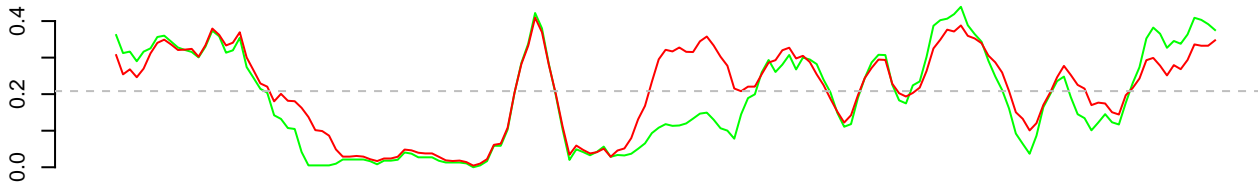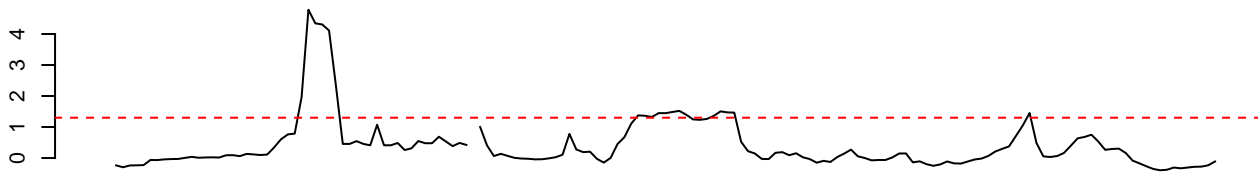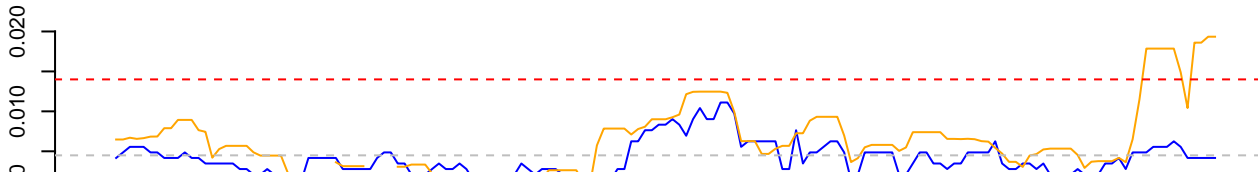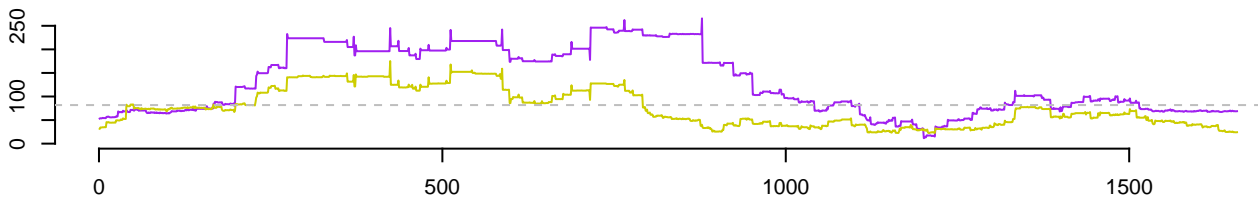

Supplement: Supplemental Material [file supp_g3.116.029215_FileS2.zip › MG5_plus.pdf]

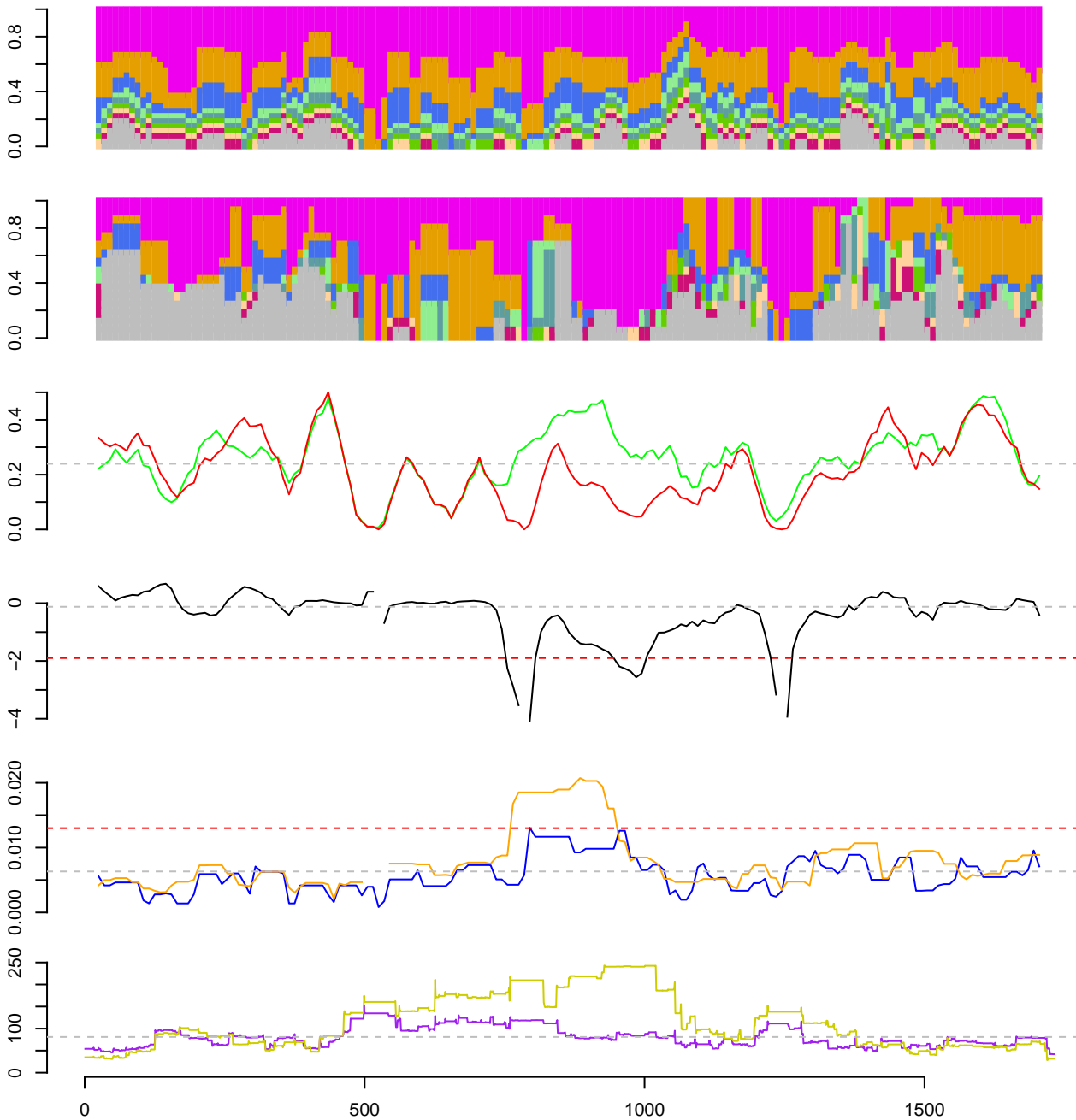

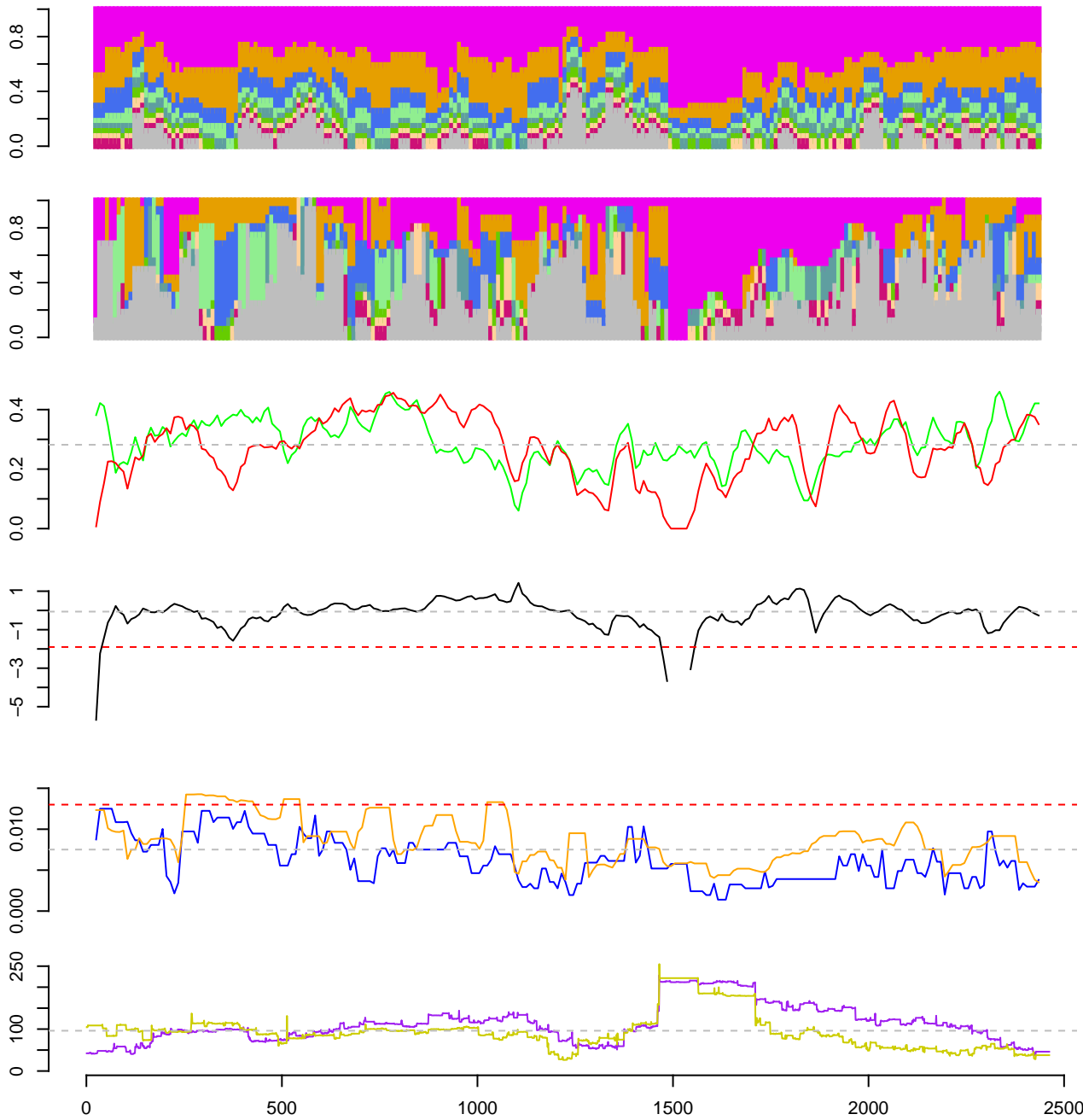

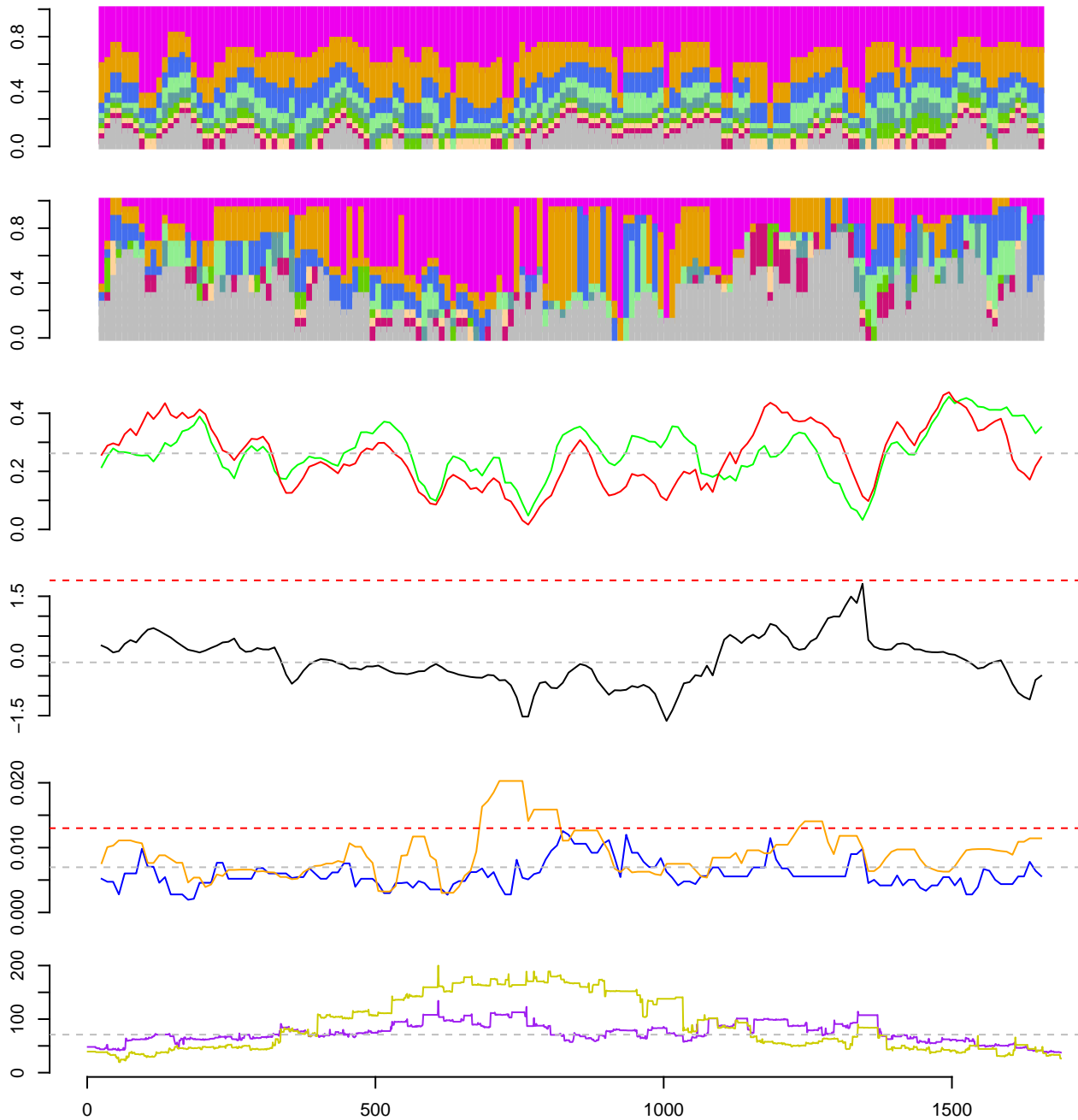

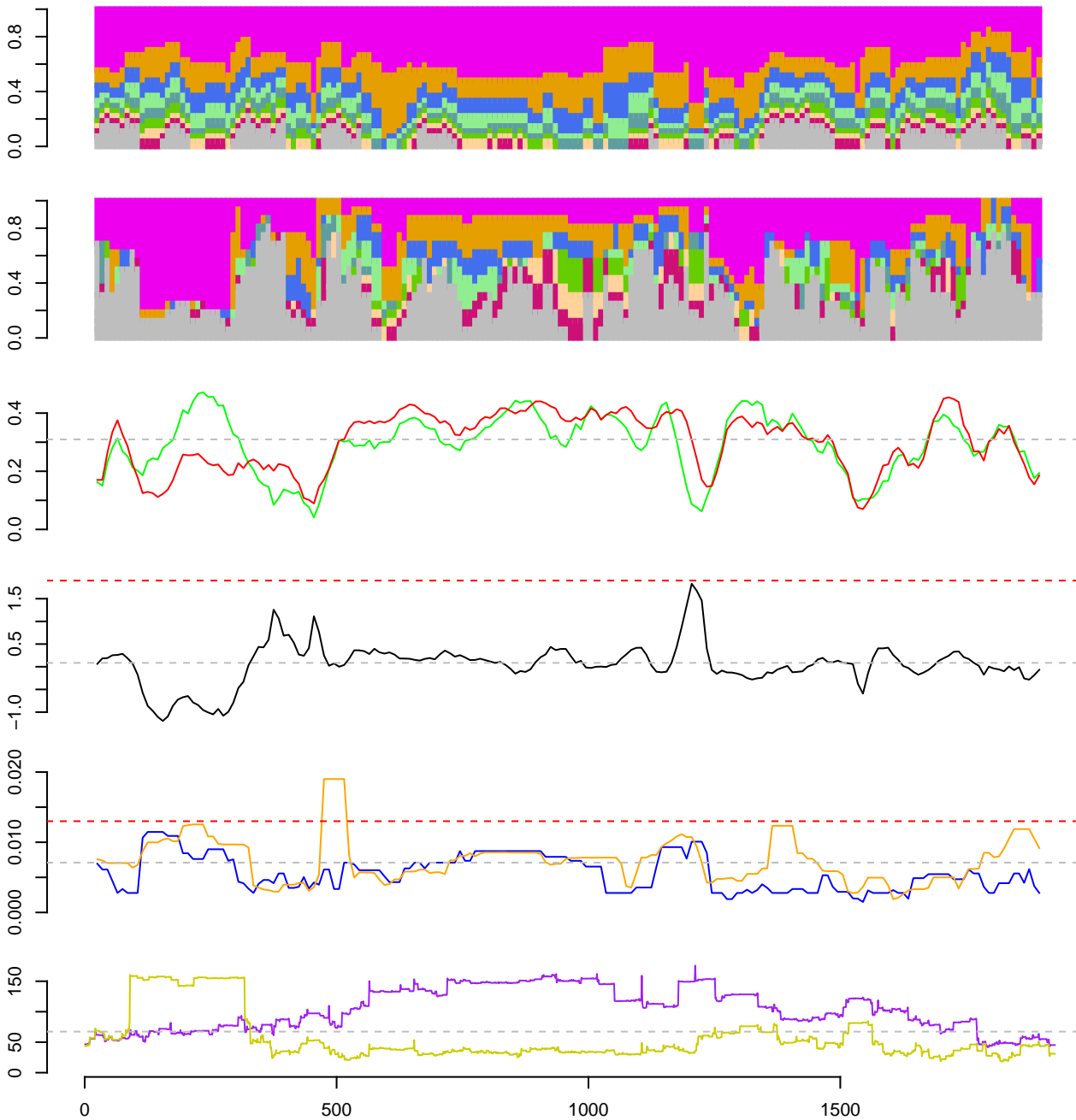

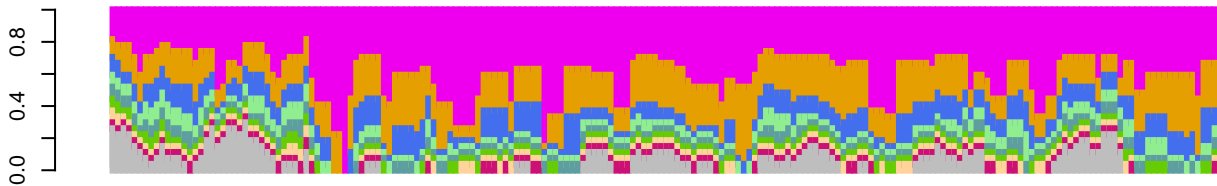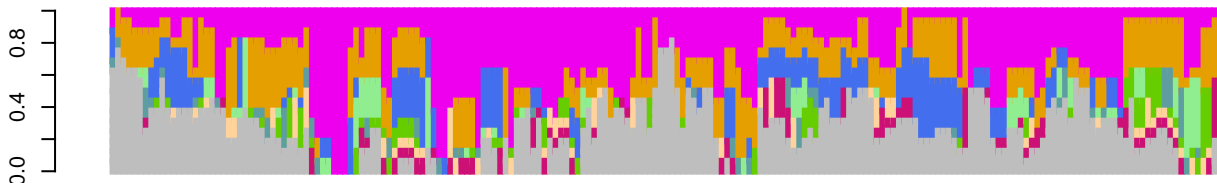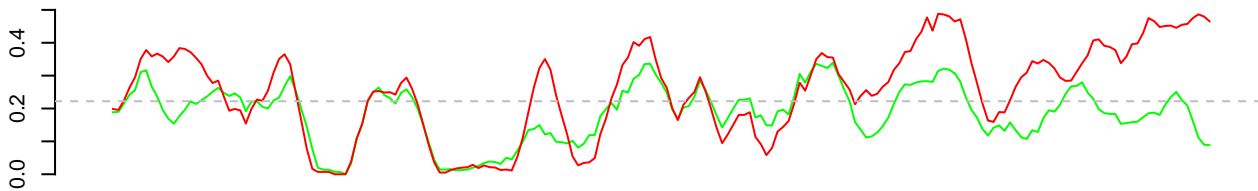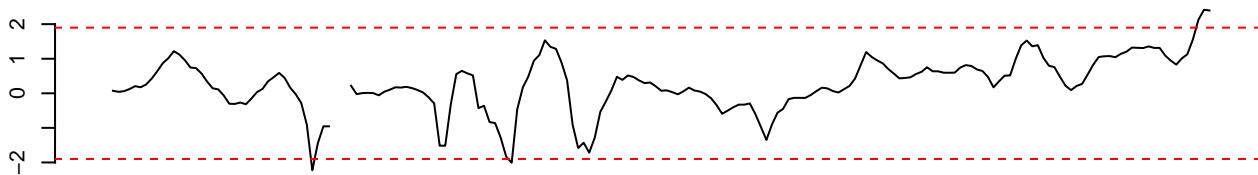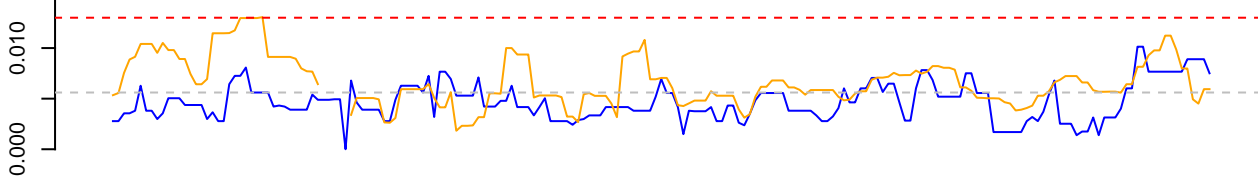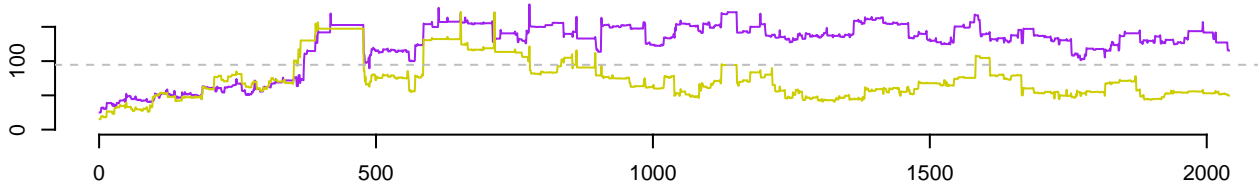

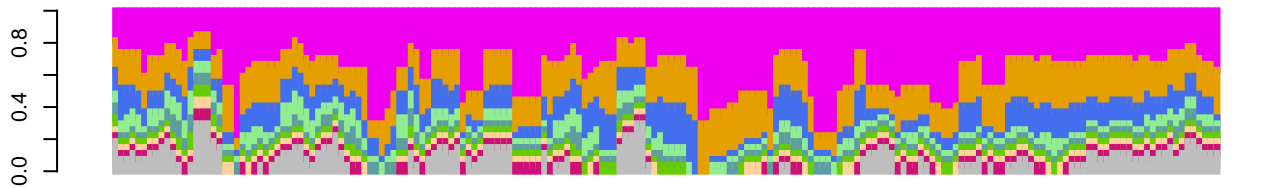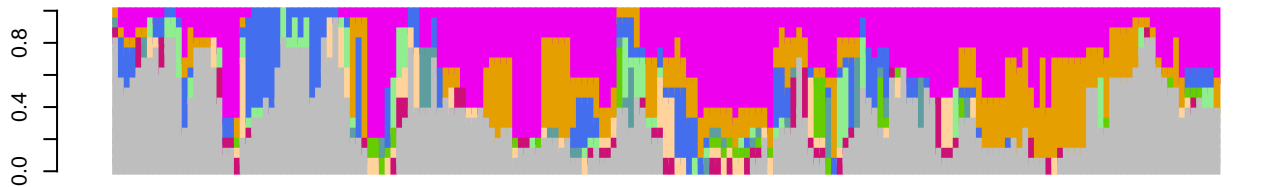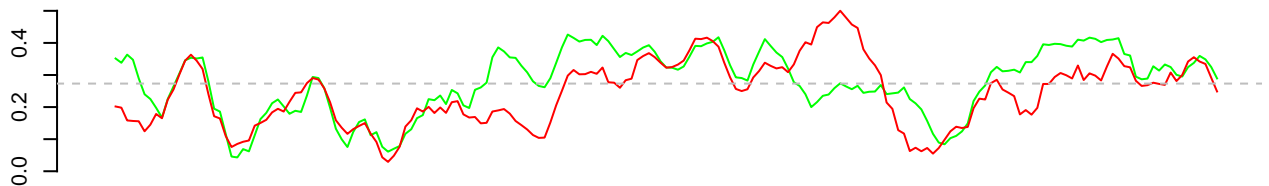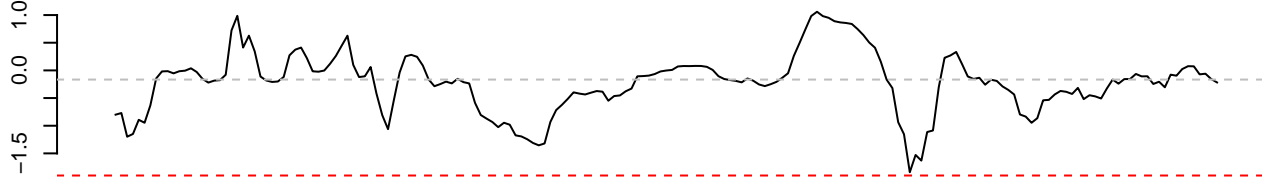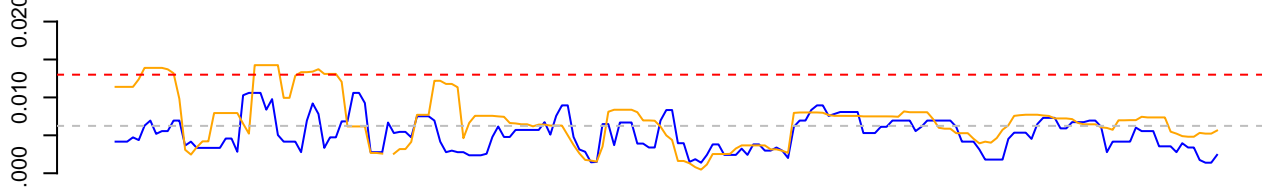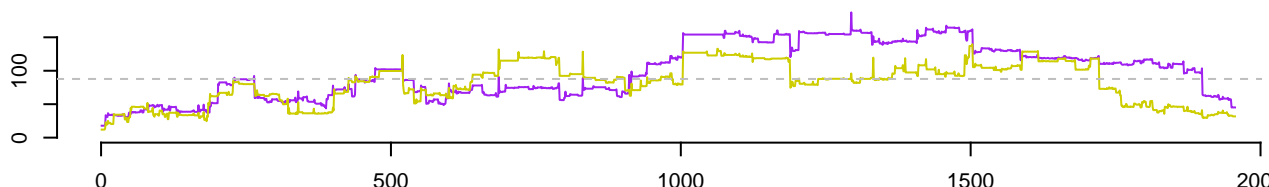

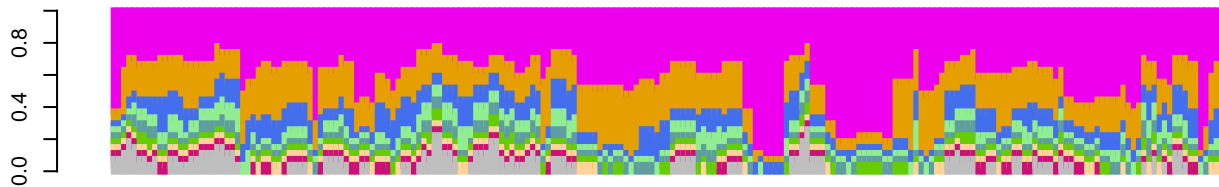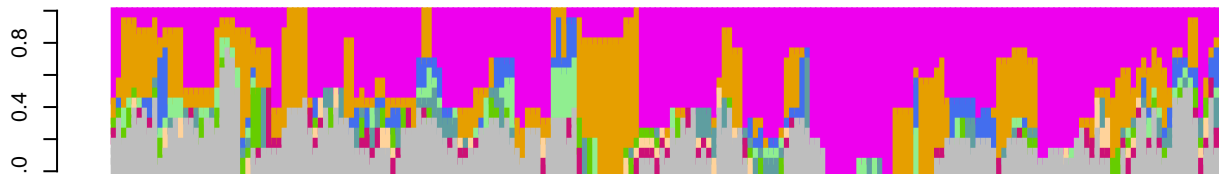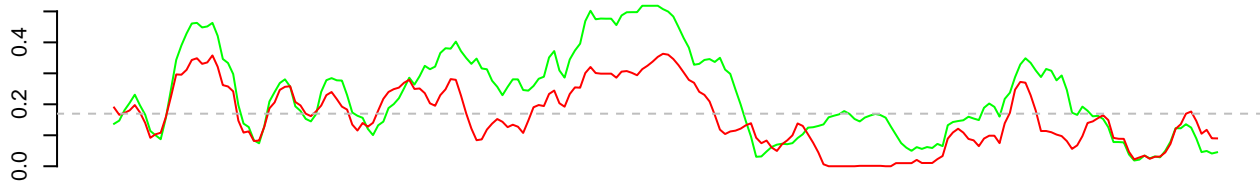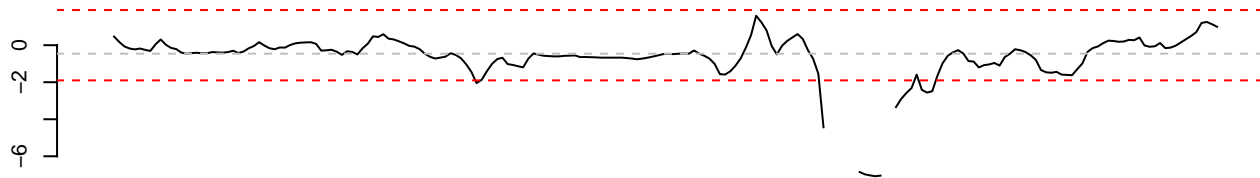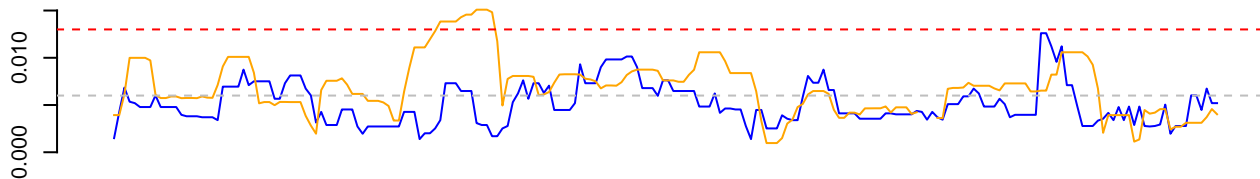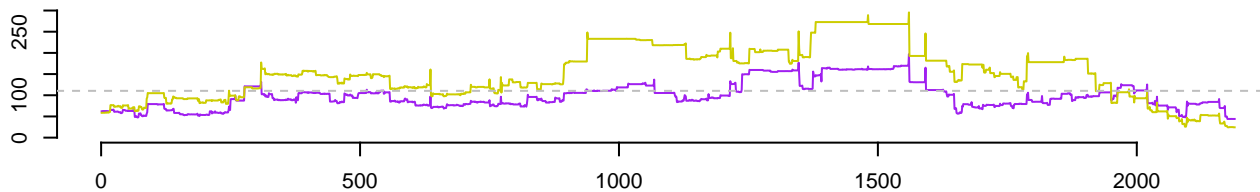

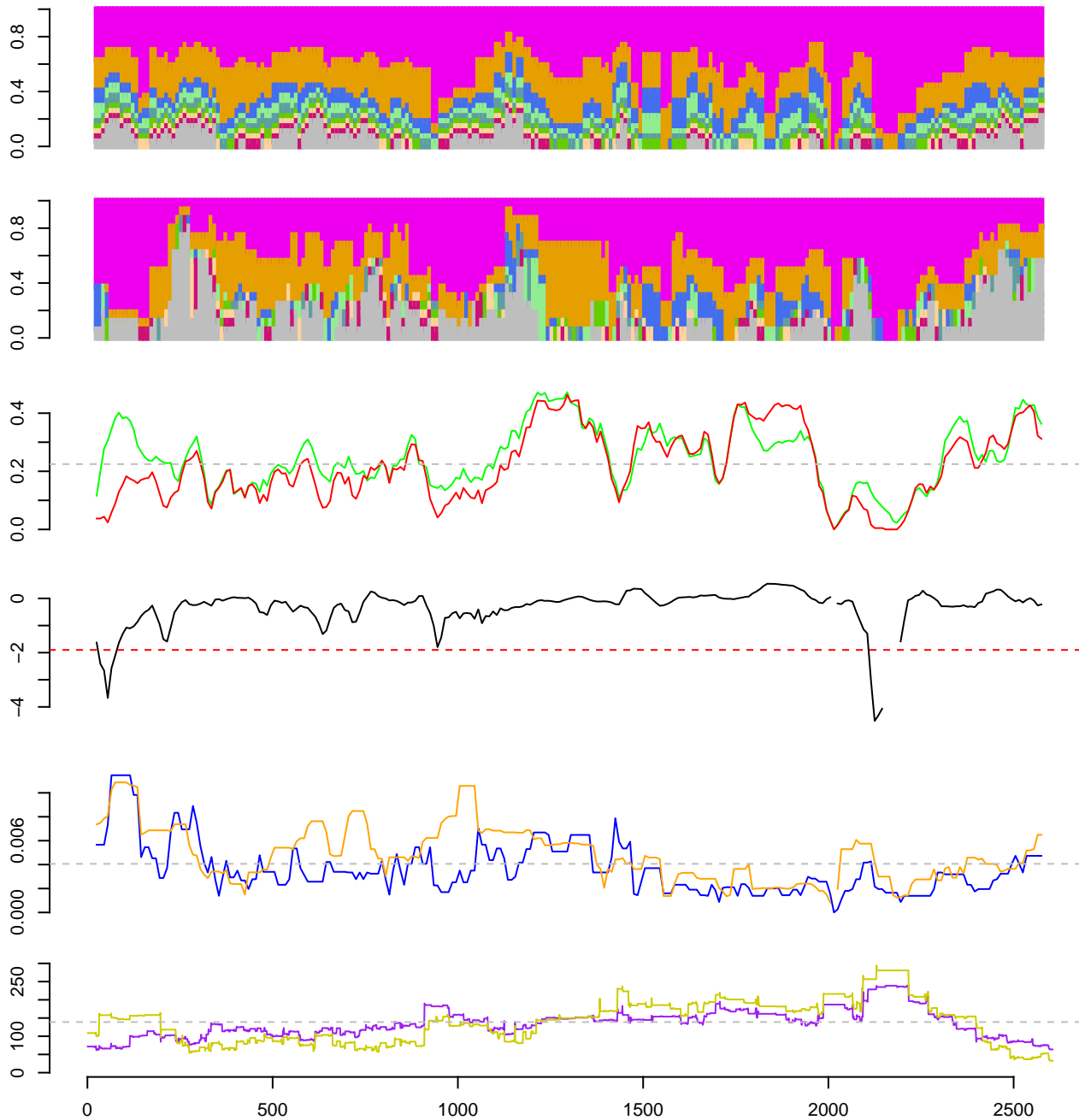

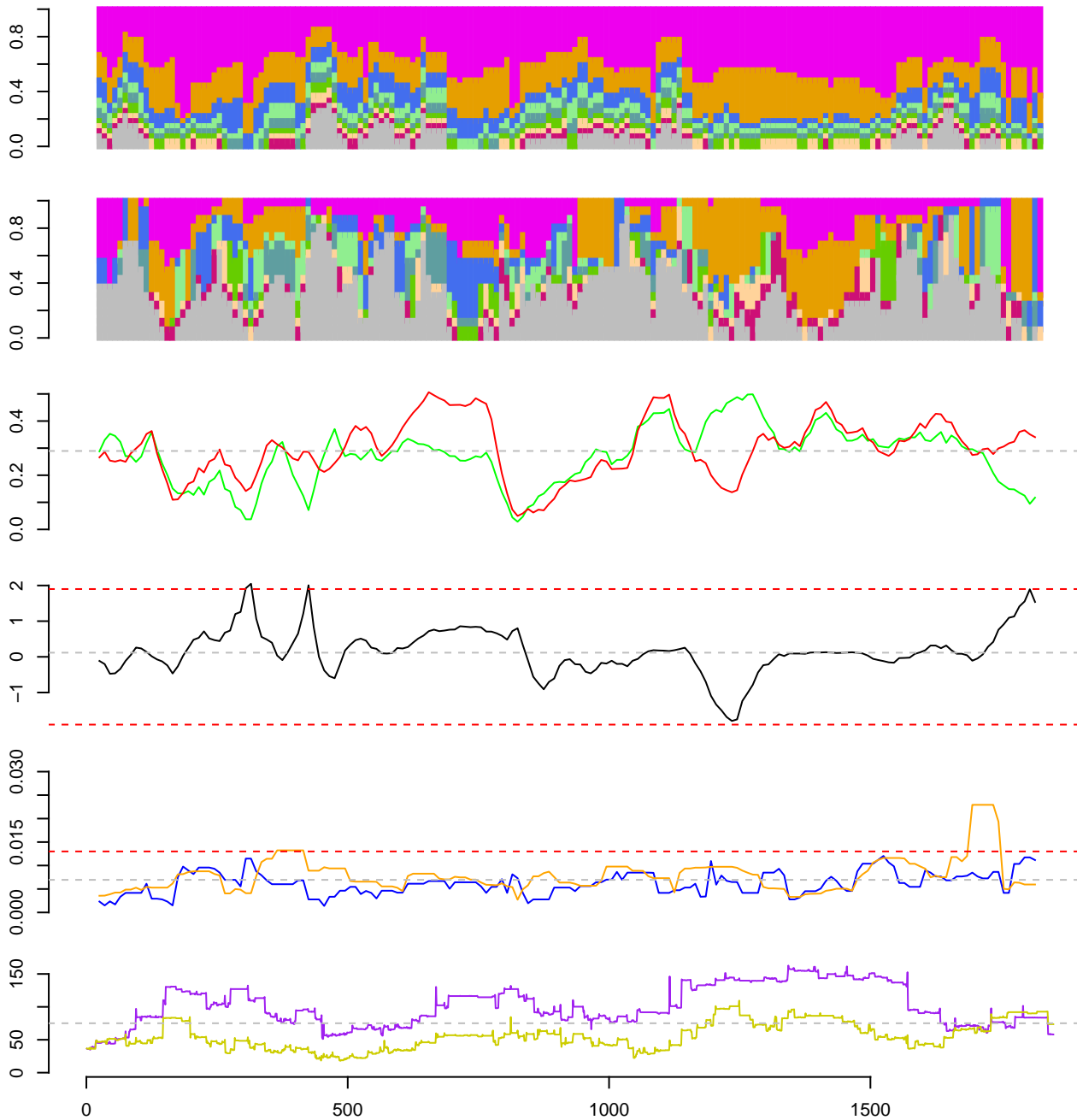

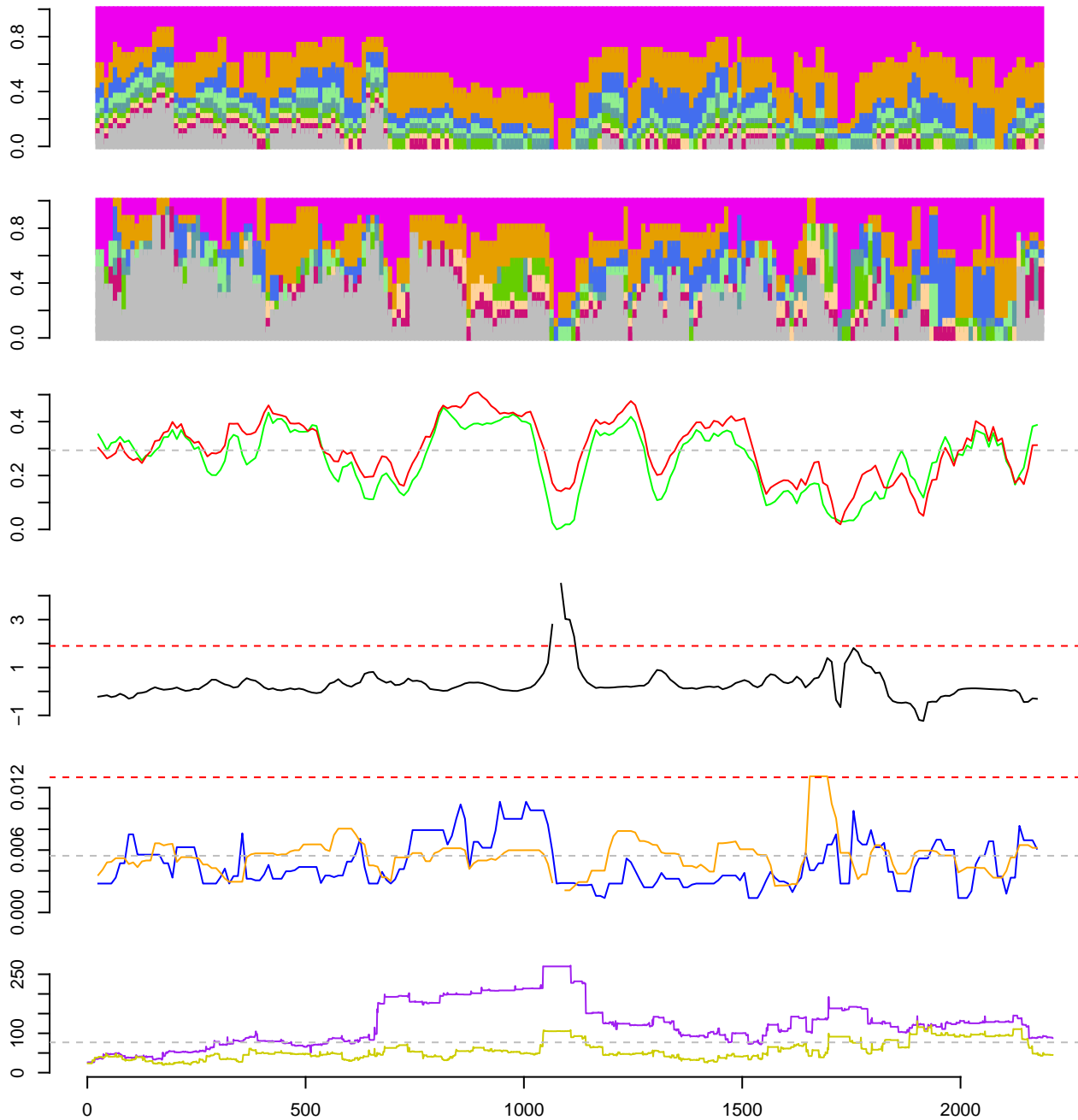

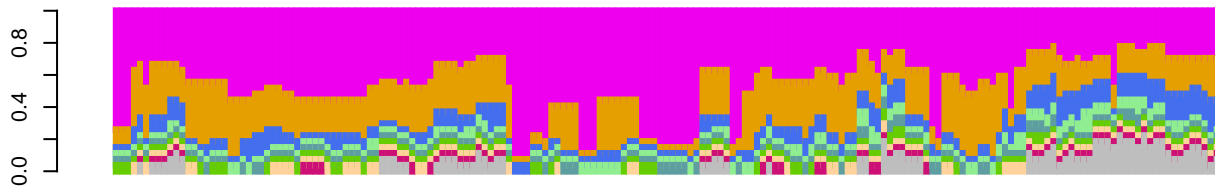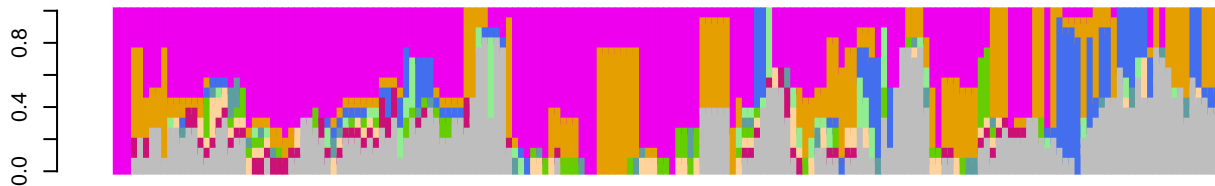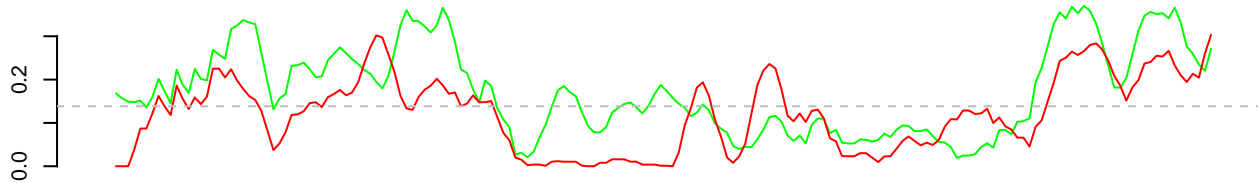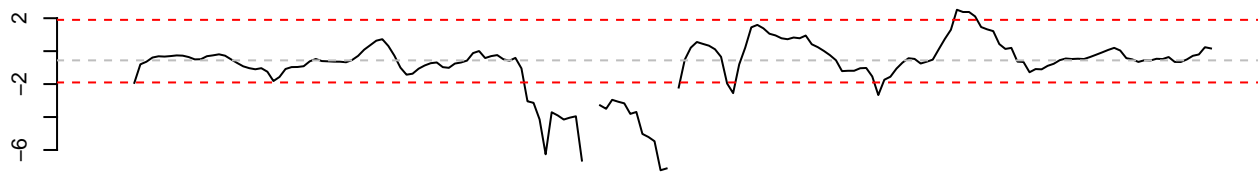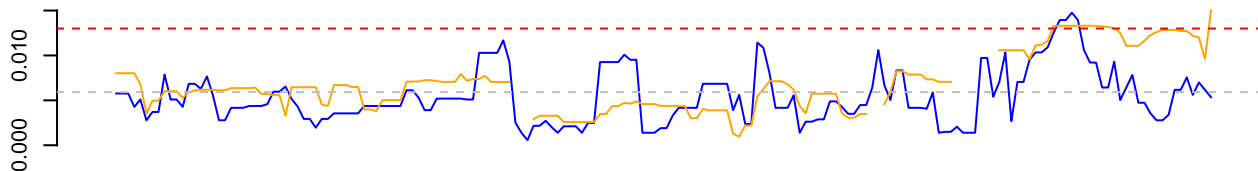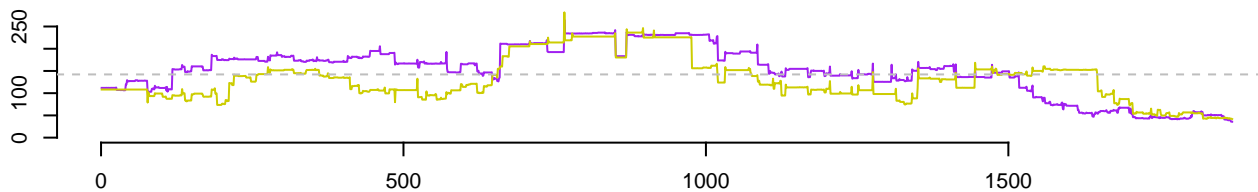

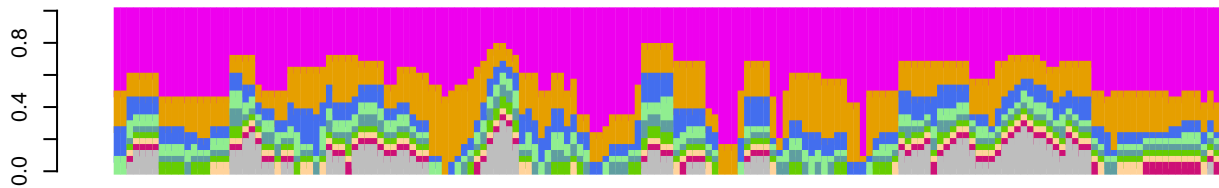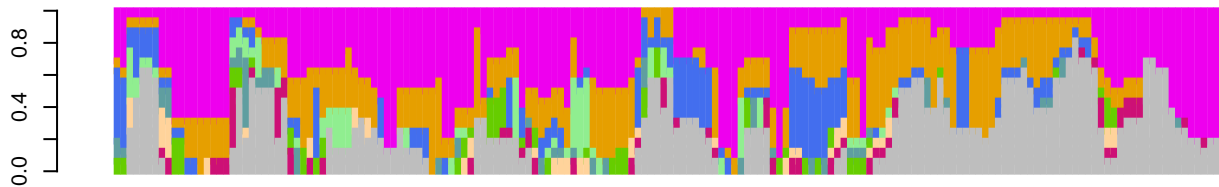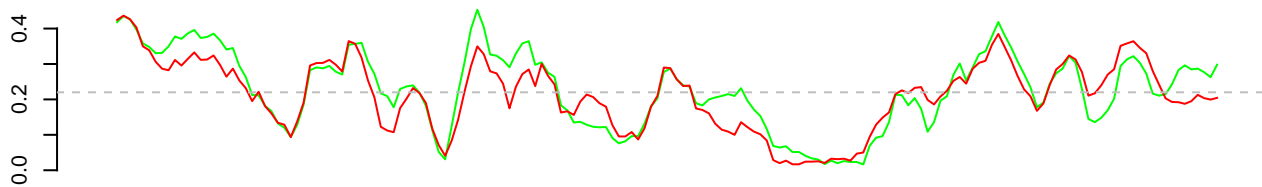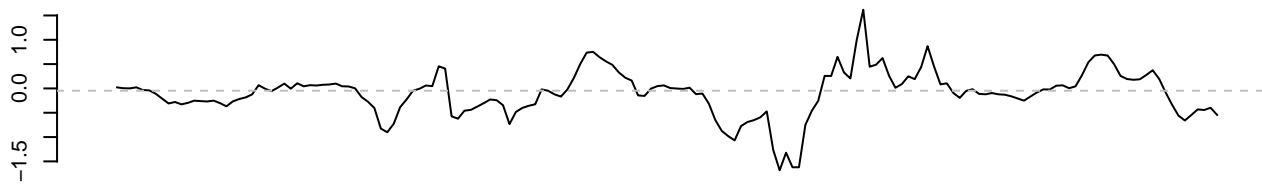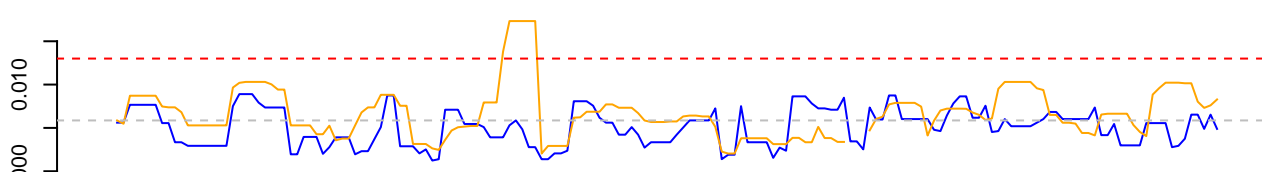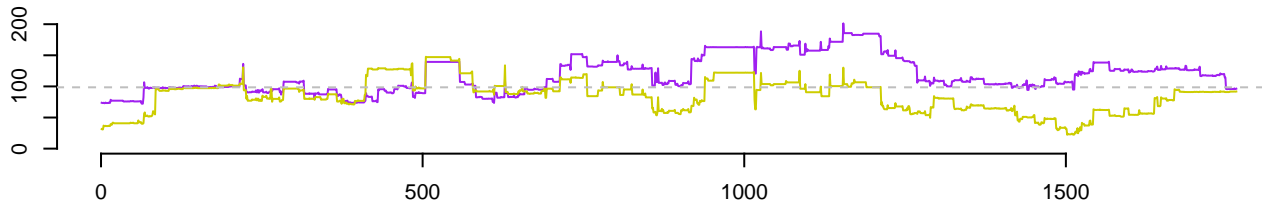

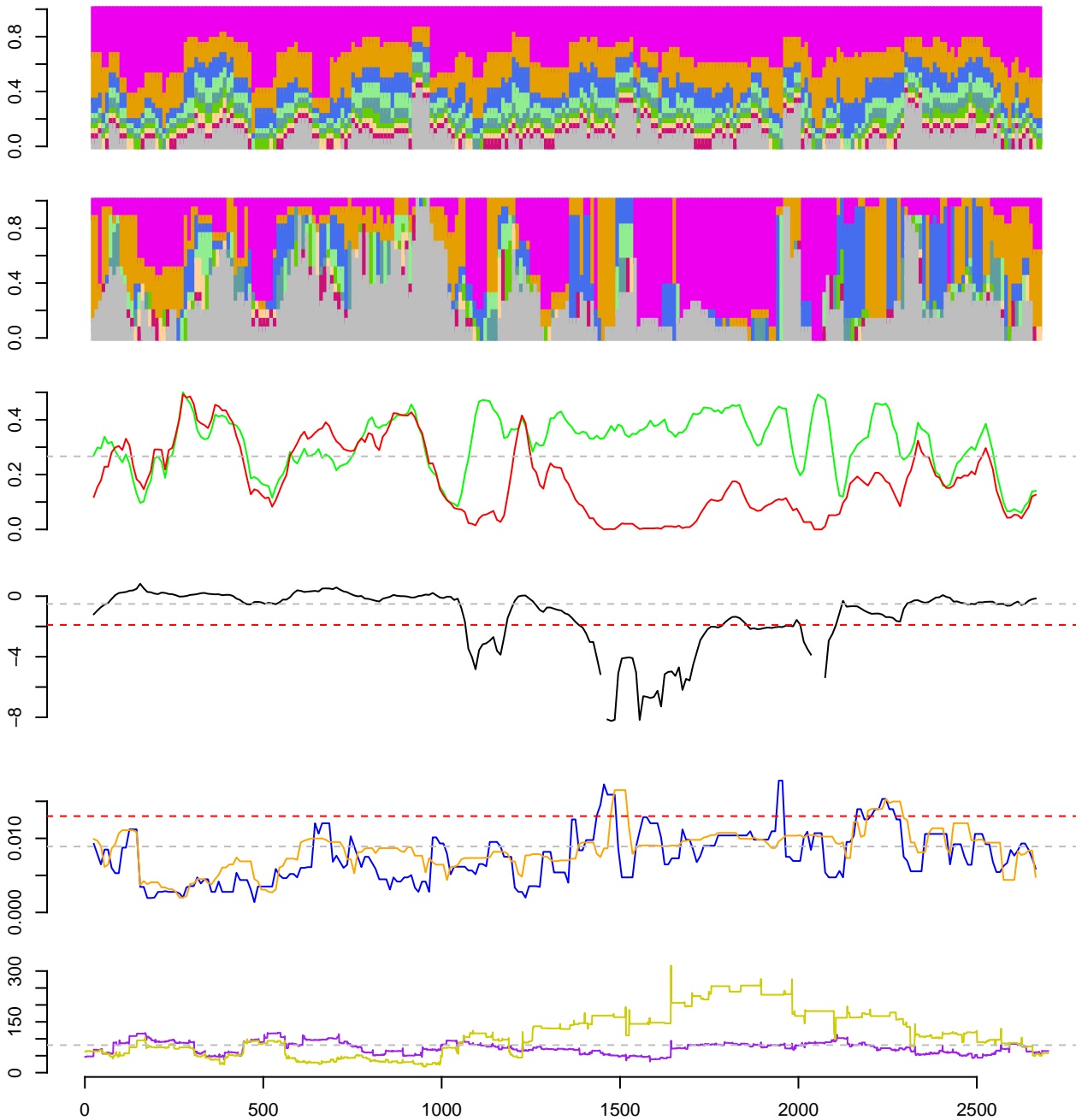

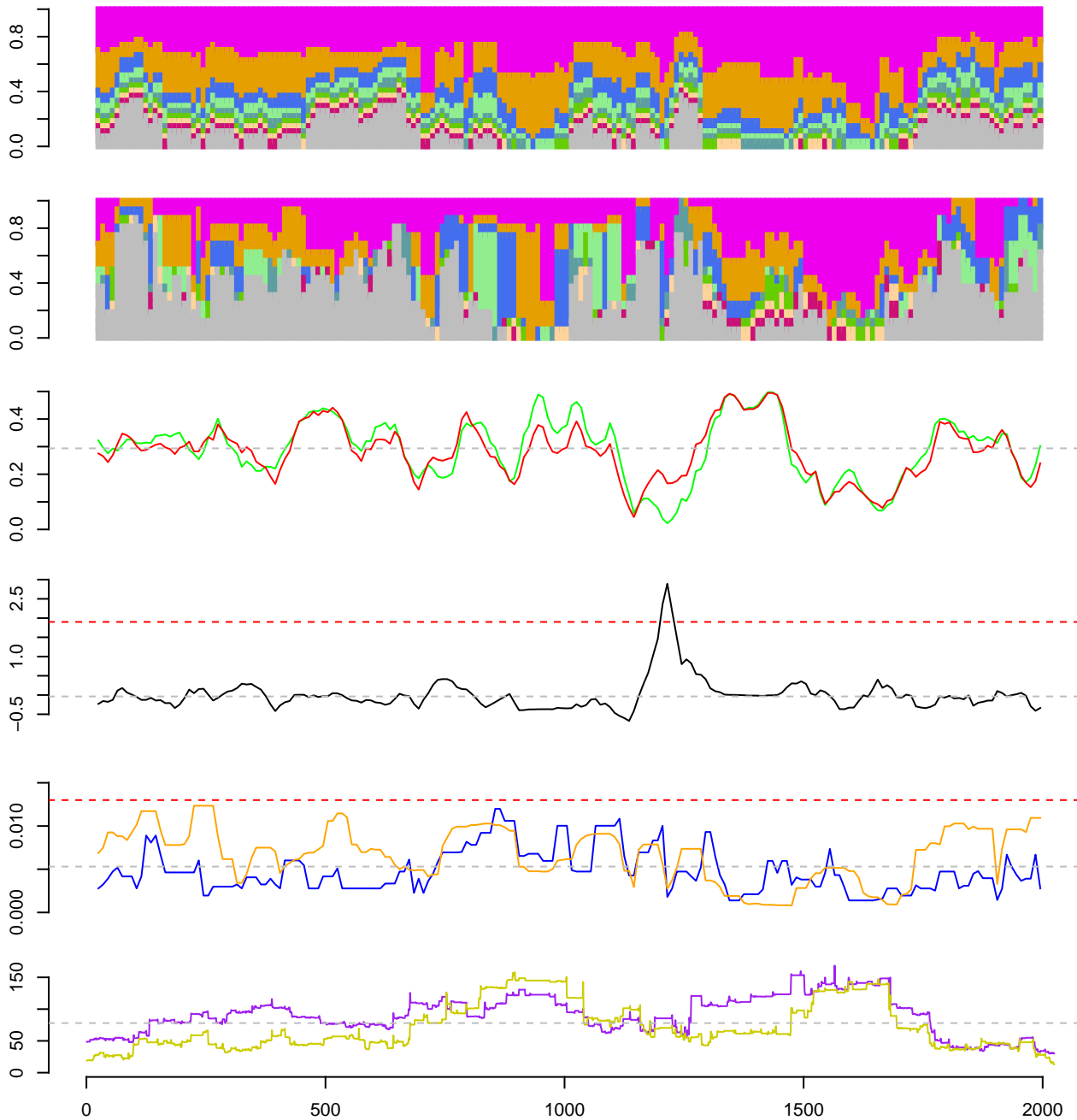

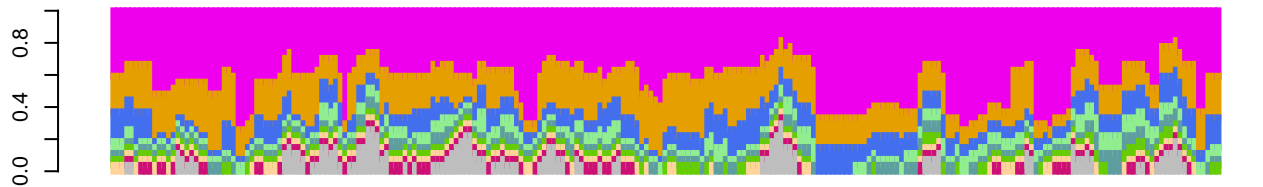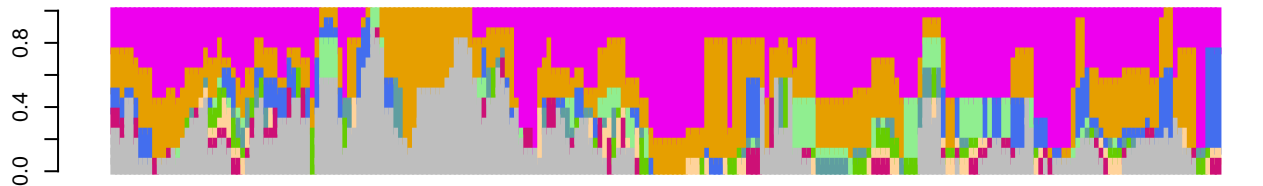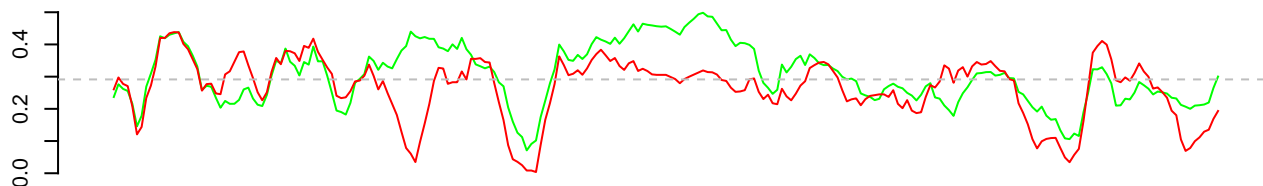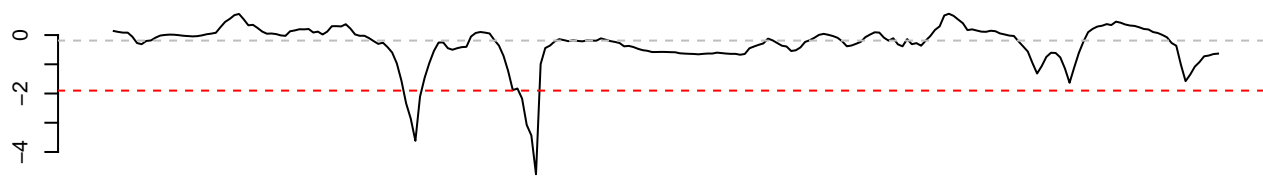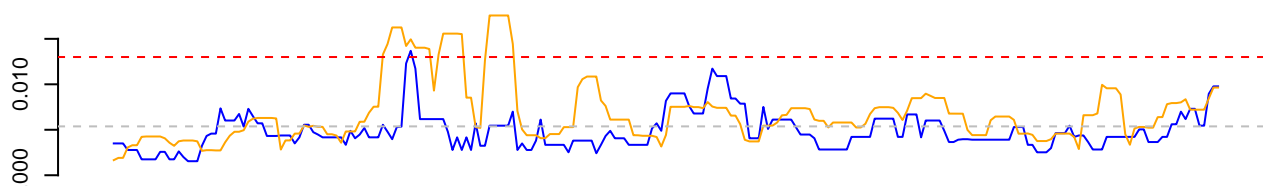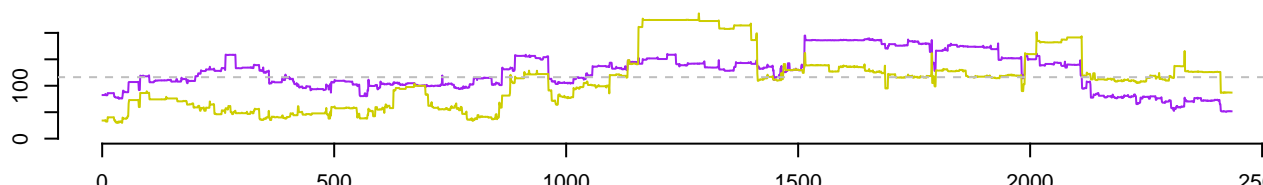

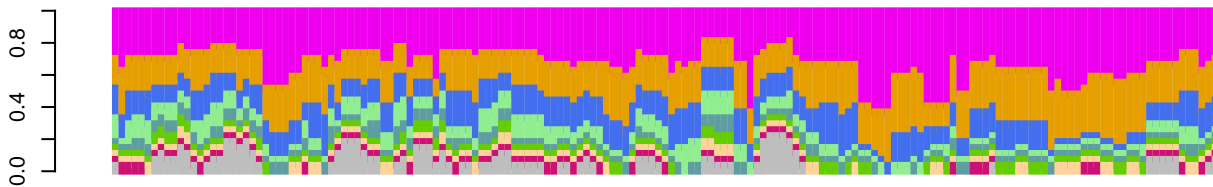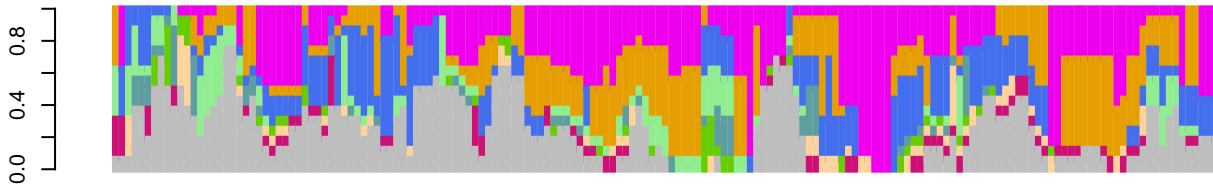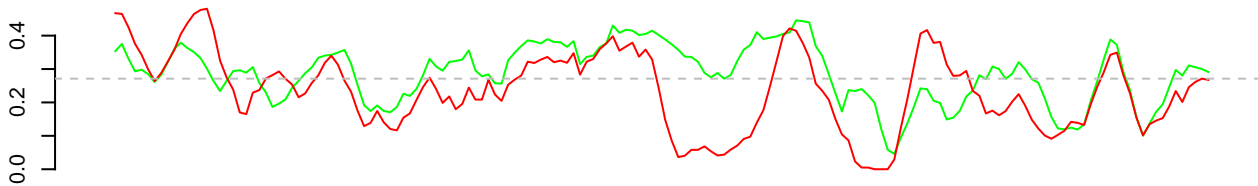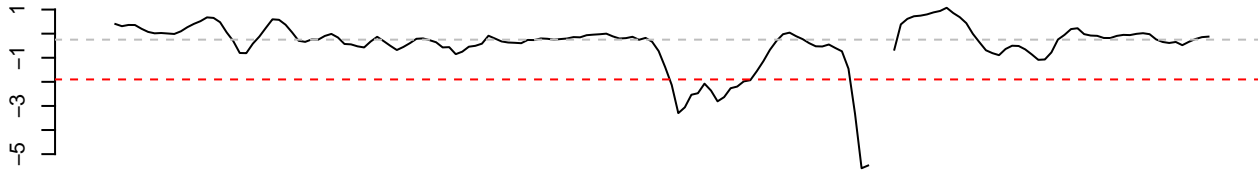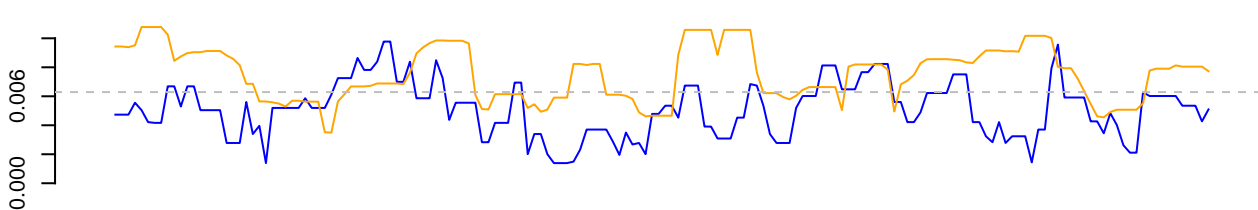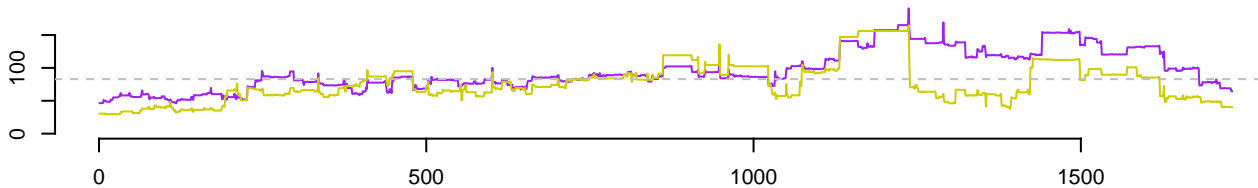

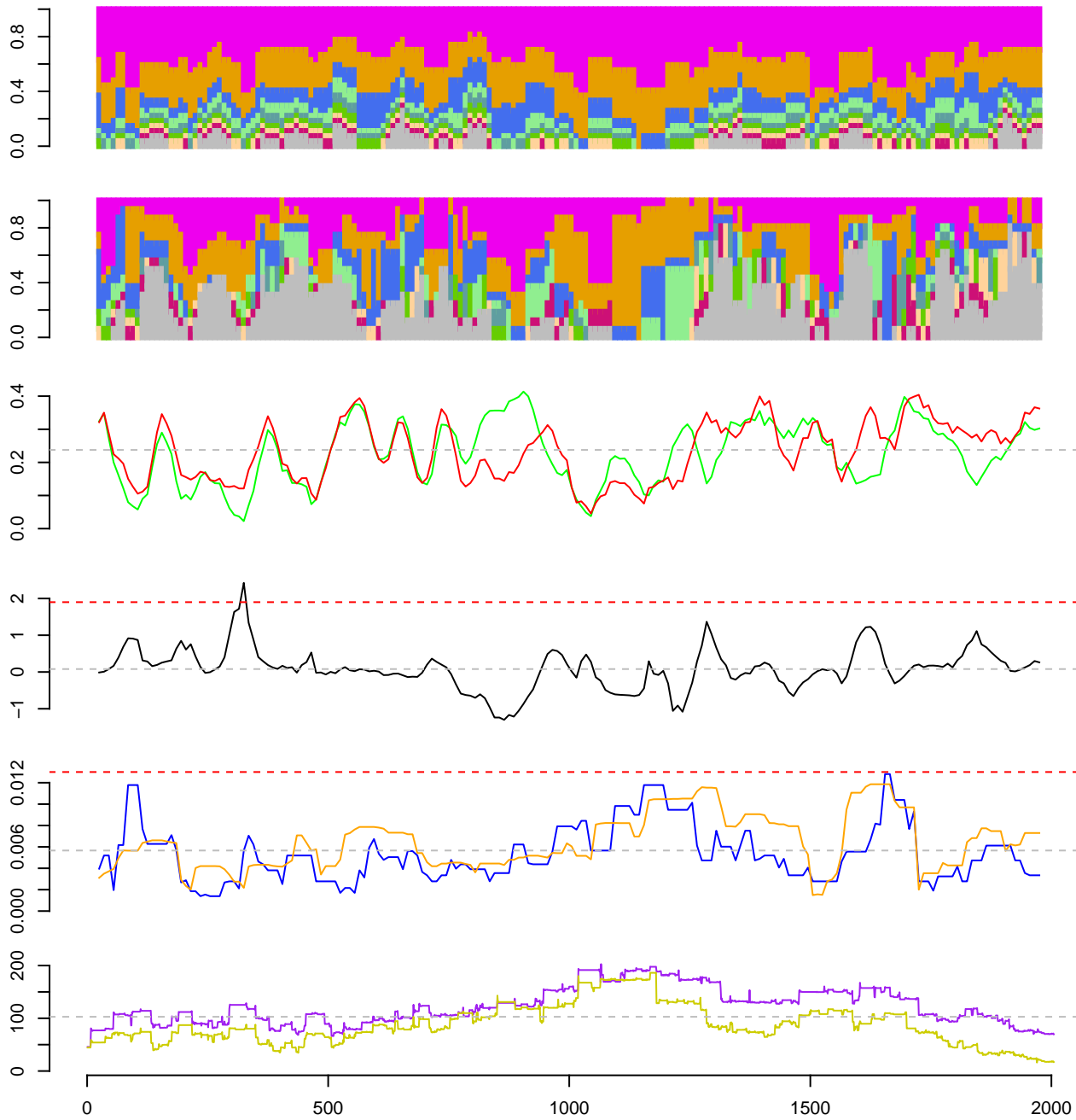

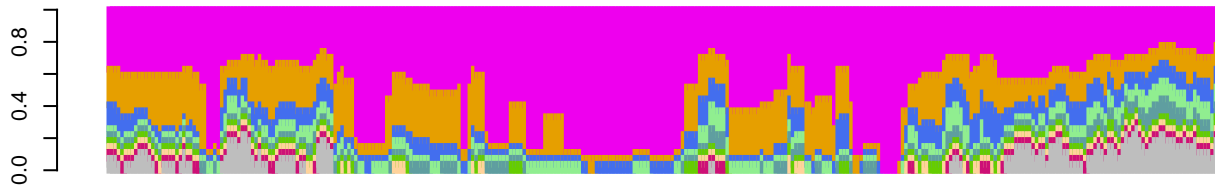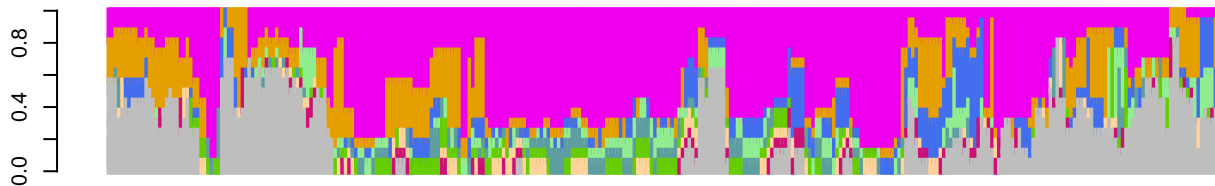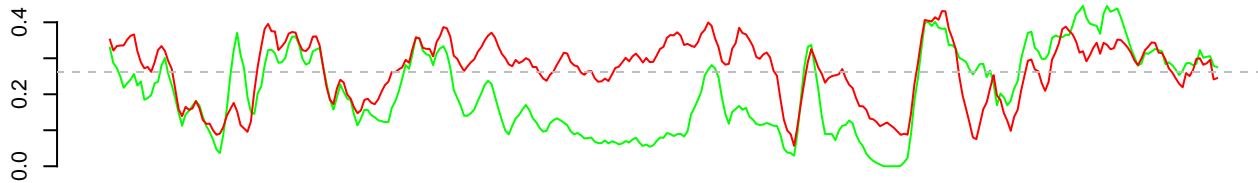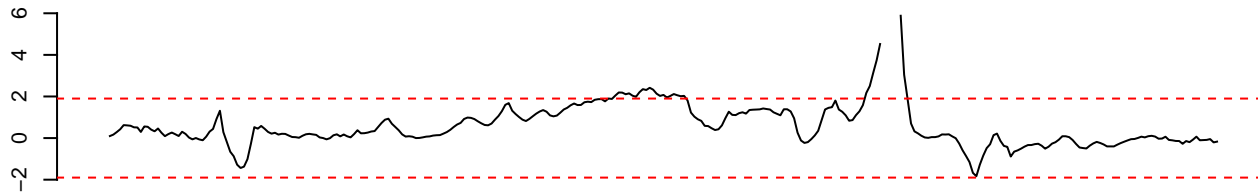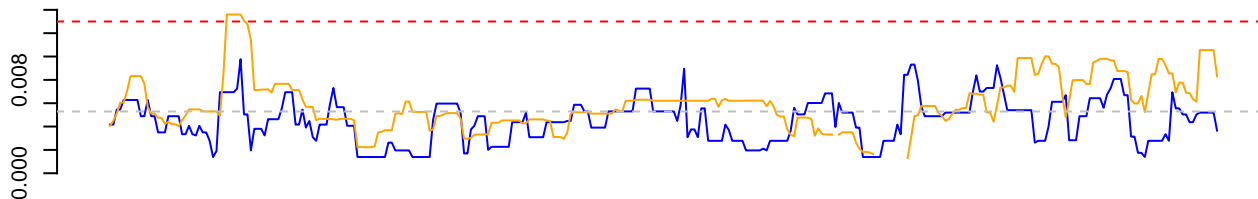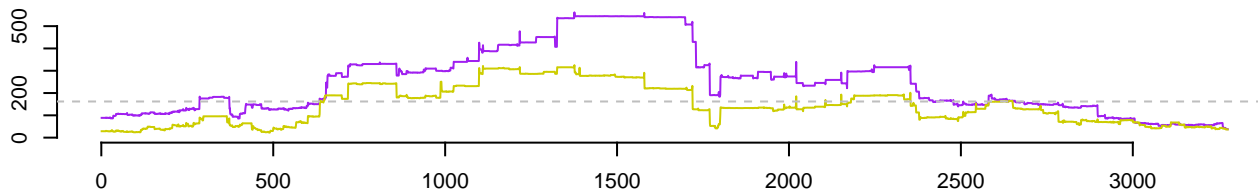

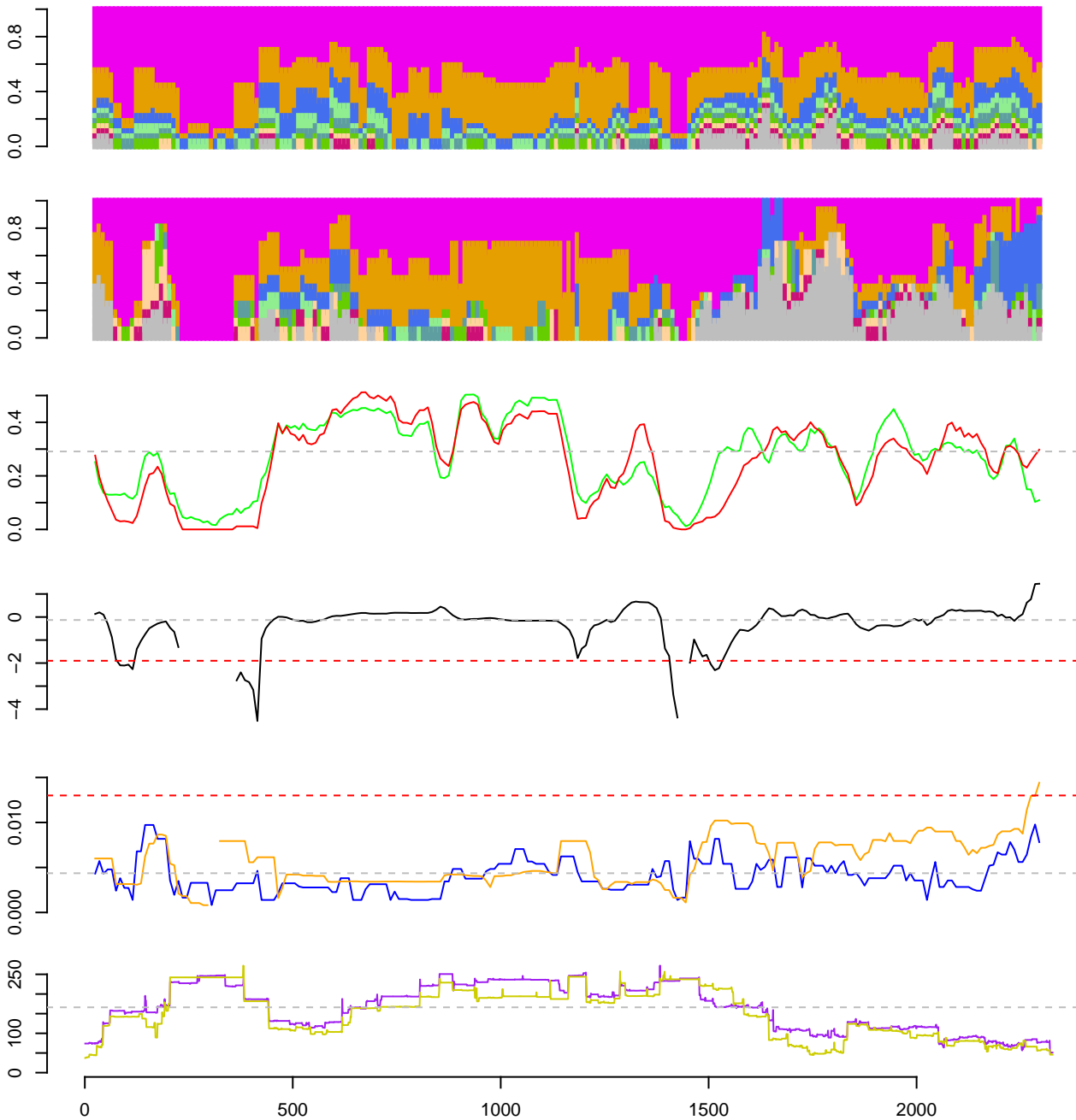

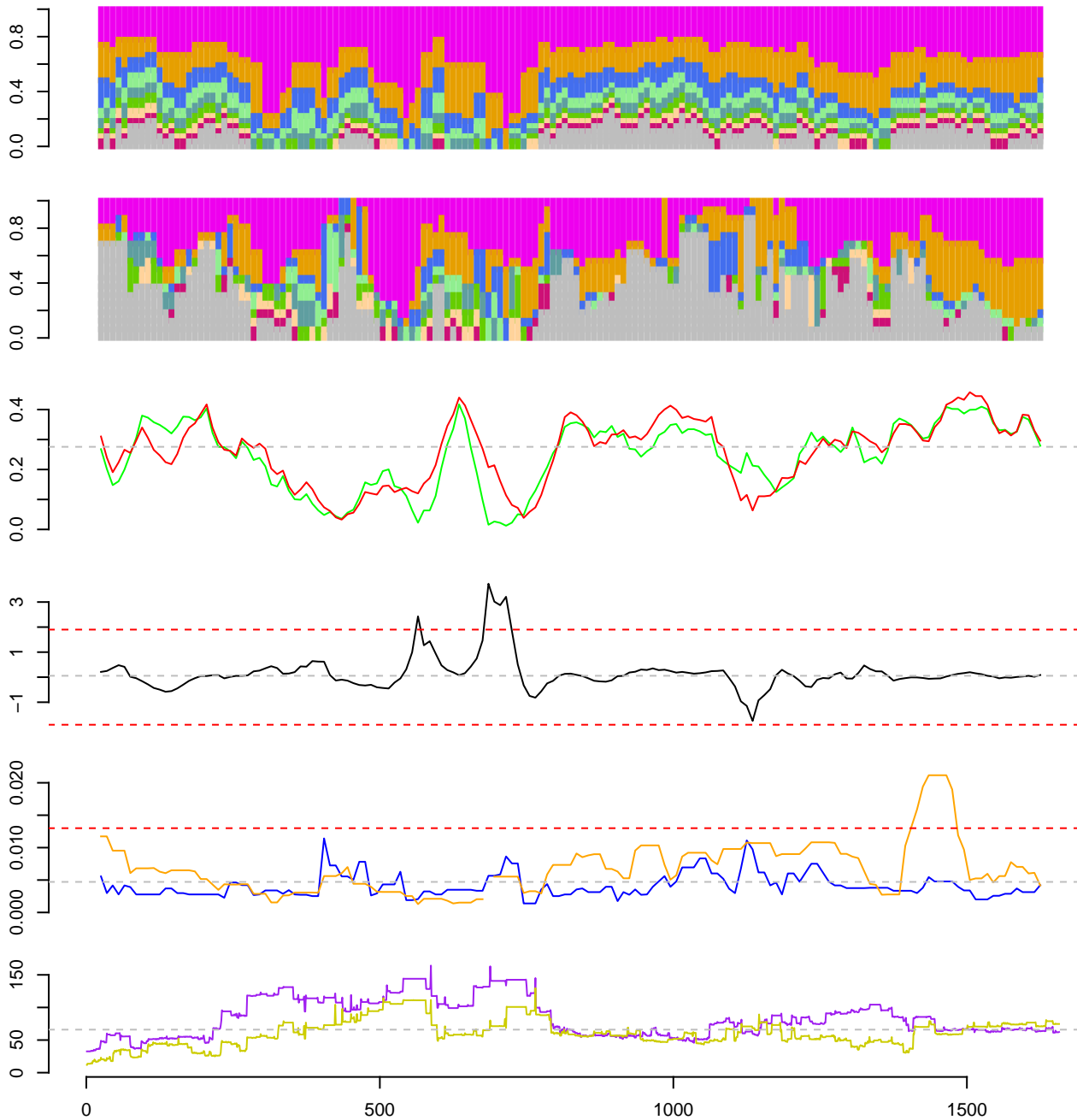

Supplement: Supplemental Material [file supp_g3.116.029215_FileS2.zip › MG0_1.pdf]
