# Supplementary material for: Genomic Signatures of North American Soybean Improvement Inform Diversity Enrichment Strategies and Clarify the Impact of Hybridization
Source: G3 (Bethesda). 2016 Jul 7;6(9):2693–705. doi: 10.1534/g3.116.029215 (PMC5015928; doi:10.1534/g3.116.029215)
Supplement: Supplemental Material [file supp_g3.116.029215_FileS5.zip › histograms/linear.pdf]

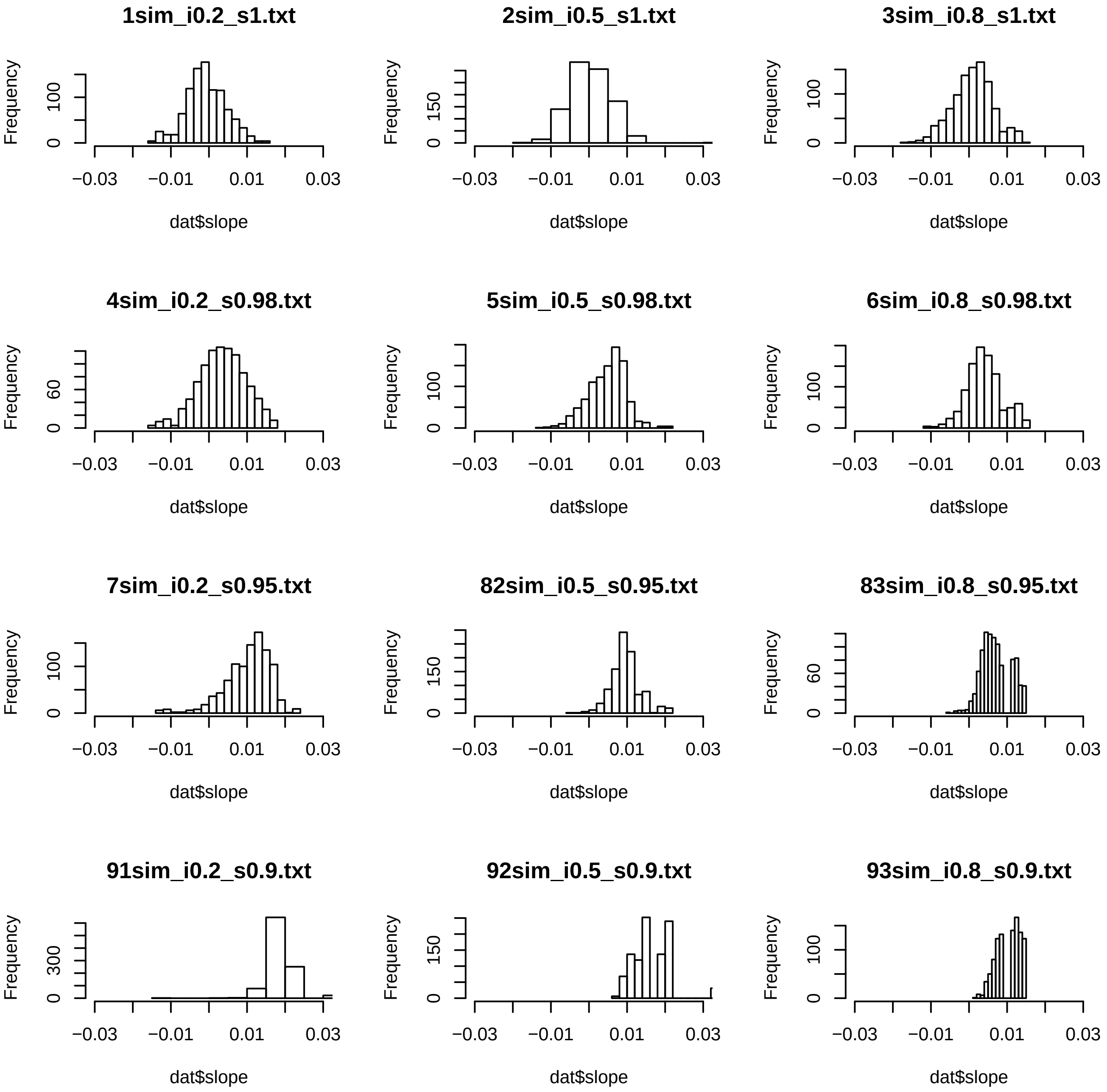
1sim_i0.2_s1.txt
2sim_i0.5_s1.txt
3sim_i0.8_s1.txt
4sim_i0.2_s0.98.txt
5sim_i0.5_s0.98.txt
6sim_i0.8_s0.98.txt
7sim_i0.2_s0.95.txt
82sim_i0.5_s0.95.txt
83sim_i0.8_s0.95.txt
91sim_i0.2_s0.9.txt
92sim_i0.5_s0.9.txt
93sim_i0.8_s0.9.txt
Frequency
dat$slope
−0.03
−0.01
0.01
0.03
0
60
100
150
300
