# Supplementary material for: Genomic Signatures of North American Soybean Improvement Inform Diversity Enrichment Strategies and Clarify the Impact of Hybridization
Source: G3 (Bethesda). 2016 Jul 7;6(9):2693–705. doi: 10.1534/g3.116.029215 (PMC5015928; doi:10.1534/g3.116.029215)
Supplement: Supplemental Material [file supp_g3.116.029215_FileS5.zip › histograms/logistic.pdf]

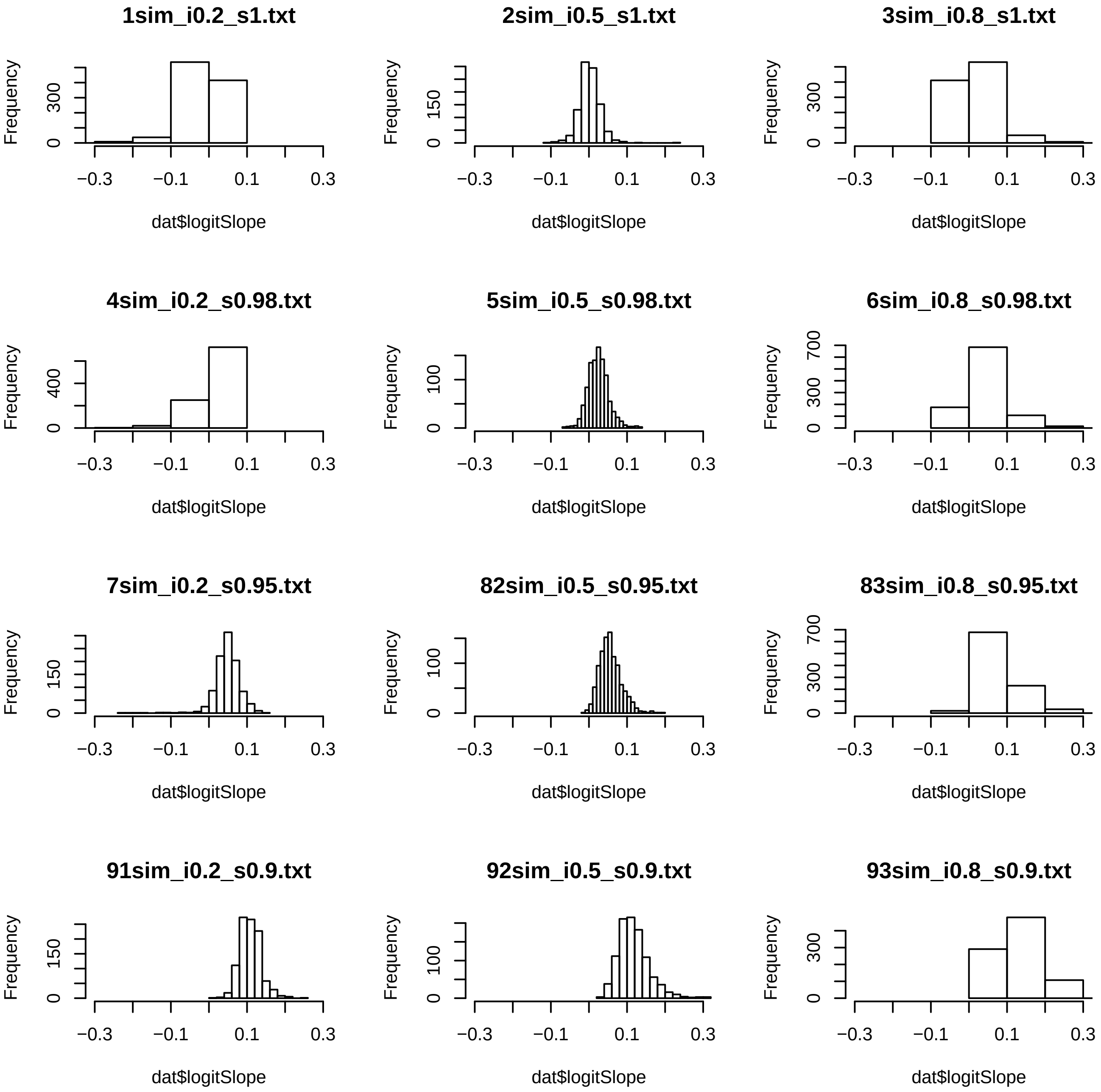
1sim_i0.2_s1.txt
2sim_i0.5_s1.txt
3sim_i0.8_s1.txt
4sim_i0.2_s0.98.txt
5sim_i0.5_s0.98.txt
6sim_i0.8_s0.98.txt
7sim_i0.2_s0.95.txt
82sim_i0.5_s0.95.txt
83sim_i0.8_s0.95.txt
91sim_i0.2_s0.9.txt
92sim_i0.5_s0.9.txt
93sim_i0.8_s0.9.txt
Frequency
dat$logitSlope
−0.3
−0.1
0.1
0.3
0
100
150
300
400
700
