# Supplementary material for: Genomic Signatures of North American Soybean Improvement Inform Diversity Enrichment Strategies and Clarify the Impact of Hybridization
Source: G3 (Bethesda). 2016 Jul 7;6(9):2693–705. doi: 10.1534/g3.116.029215 (PMC5015928; doi:10.1534/g3.116.029215)
Supplement: Supplemental Material [file supp_g3.116.029215_FileS5.zip › histograms/WFABC.pdf]

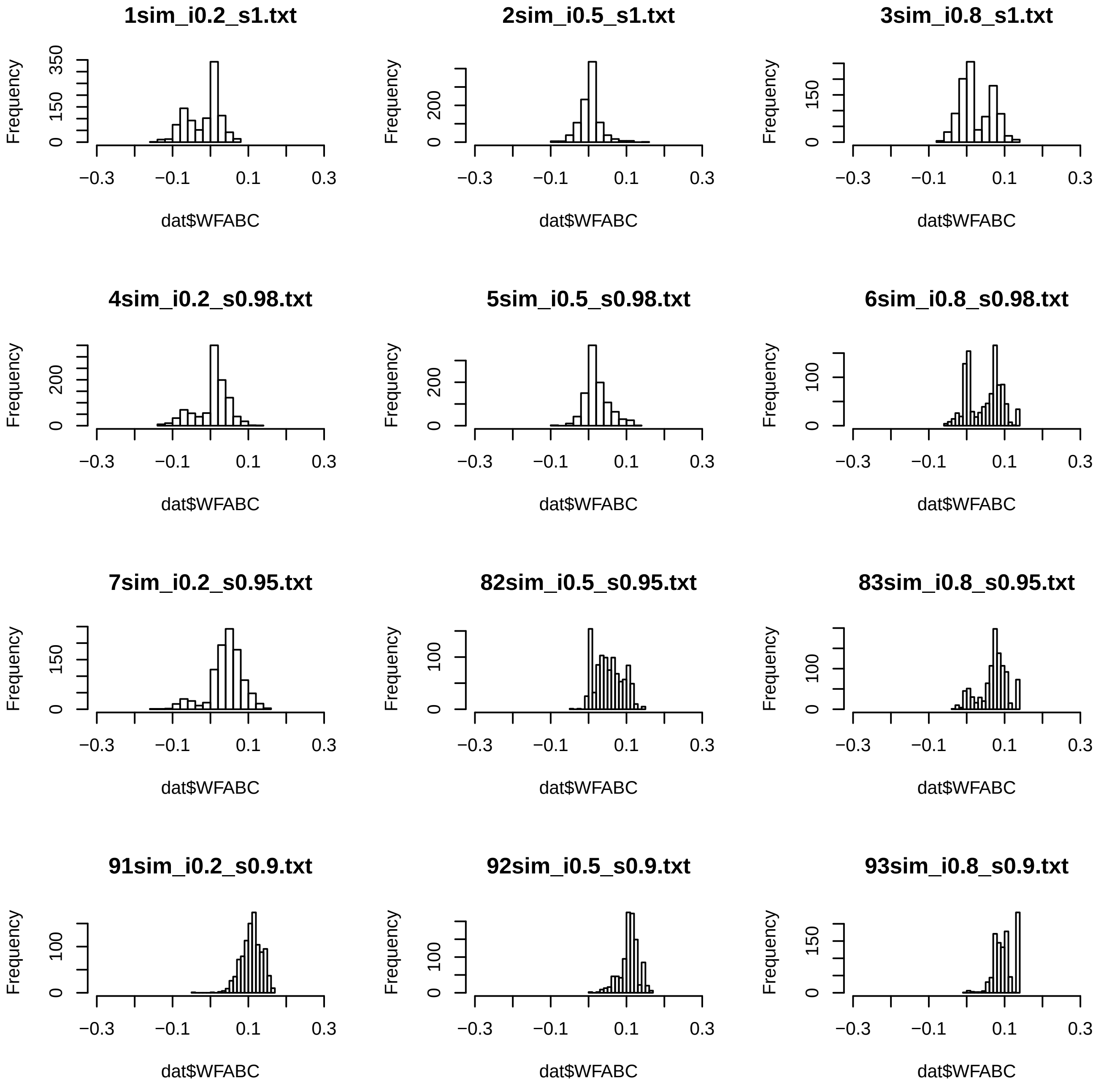
1sim_i0.2_s1.txt
Frequency
0
150
350
−0.3
−0.1
0.1
0.3
dat$WFABC
2sim_i0.5_s1.txt
Frequency
0
200
−0.3
−0.1
0.1
0.3
dat$WFABC
3sim_i0.8_s1.txt
Frequency
0
150
−0.3
−0.1
0.1
0.3
dat$WFABC
4sim_i0.2_s0.98.txt
Frequency
0
200
−0.3
−0.1
0.1
0.3
dat$WFABC
5sim_i0.5_s0.98.txt
Frequency
0
200
−0.3
−0.1
0.1
0.3
dat$WFABC
6sim_i0.8_s0.98.txt
Frequency
0
100
−0.3
−0.1
0.1
0.3
dat$WFABC
7sim_i0.2_s0.95.txt
Frequency
0
150
−0.3
−0.1
0.1
0.3
dat$WFABC
82sim_i0.5_s0.95.txt
Frequency
0
100
−0.3
−0.1
0.1
0.3
dat$WFABC
83sim_i0.8_s0.95.txt
Frequency
0
100
−0.3
−0.1
0.1
0.3
dat$WFABC
91sim_i0.2_s0.9.txt
Frequency
0
100
−0.3
−0.1
0.1
0.3
dat$WFABC
92sim_i0.5_s0.9.txt
Frequency
0
100
−0.3
−0.1
0.1
0.3
dat$WFABC
93sim_i0.8_s0.9.txt
Frequency
0
150
−0.3
−0.1
0.1
0.3
dat$WFABC
